# Sustainability and Integrated Energy Recovery Scheme for Health Care Waste Management for Megacity Dhaka: Through Material Flow and Life Cycle Assessment

**DOI:** 10.1002/gch2.70172

**Published:** 2026-10-31

**Authors:** Mahmudul Hasan Sajid, S. M. Akimus Salehin, Musfekur Rahman Dihan, Rayhan Bin Masud, Rumana Afrin, Md. Shahinoor Islam, Md. Mujibur Rahman

**Affiliations:** ^1^ Department of Chemical Engineering Bangladesh University of Engineering and Technology Dhaka Bangladesh; ^2^ Department of Dyes and Chemical Engineering Bangladesh University of Textiles Dhaka Bangladesh; ^3^ Department of Chemical Engineering Dhaka University of Engineering and Technology Gazipur Bangladesh; ^4^ Department of Civil Engineering United International University (UIU) Dhaka Bangladesh

**Keywords:** circular economy, health care waste, life cycle assessment (LCA), material flow analysis, sustainability, waste valorization

## Abstract

A sustainable healthcare waste (HCW) management strategy for Dhaka integrating material flow analysis and comparative life cycle assessment is proposed. The current scenario (SC1), heavily reliant on landfilling, low recycling, and incineration without energy integration, is compared with the proposed alternative scenario (SC2), with higher material recycling, waste‐to‐energy conversion, and heat integration. Using gate‐to‐grave life‐cycle assessment across eighteen ReCiPe 2016 midpoint (H) impact categories, the proposed SC2 outperforms SC1 in ten categories, notably acidification potential by 96%, land‐use potential by 97%, climate change impacts by 78%, and marine eutrophication by 44%, while PM formation potential falls by 20.499 kg PM_2.5_‐eq and energy resource depletion becomes a net credit through 20572 kWh/day of recovered electricity. However, trade‐offs are observed in the toxicity, eutrophication, and photochemical oxidant formation categories, notably carcinogenic human toxicity (∼3.8x increase), marine ecotoxicity (∼2.3x increase), freshwater eutrophication (∼97% increase), and photochemical oxidant formation potential (∼3x increase). The proposed scheme also supports Bangladesh's drive toward SDG 7 and 13 outright, and toward SDG 3, 6, 11, and 14 once the toxicity and oxidant formation burdens are controlled. Our findings can provide a substantial quantitative framework for improving existing systems to achieve sustainability with a circular economy strategy.

## Graphical abstract

LCA and MFA are used to optimize treatment strategies of medical waste management in Dhaka city. The results reveal that waste‐to‐energy recovery, plastic recycling, and improved segregation methods followed by advanced flue gas treatment, optimized incineration conditions, and residue management systems can meaningfully improve current baseline scenario across multiple environmental impacts.

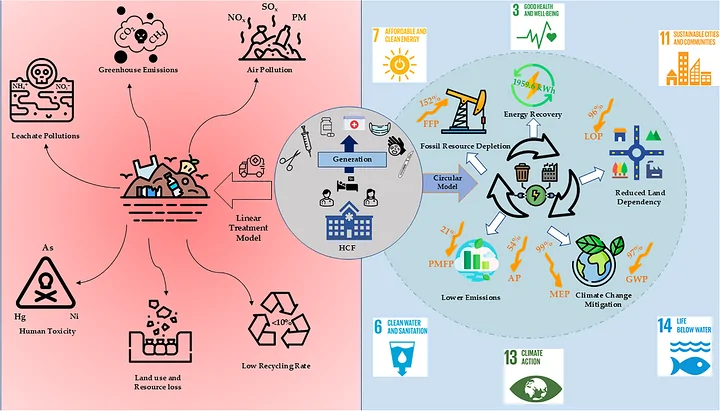

## 1 Introduction

Health care waste (HCW), also known as medical waste (MW), falls into a broad category that includes all types of waste generated in healthcare facilities like hospitals, clinics, laboratories, and research institutions [1, 2]. The World Health Organization (WHO) categorizes 85% of the generated HCW as general, non‐hazardous waste similar to municipal solid waste (MSW), while the remaining 15% being considered hazardous since it is either infectious, toxic, carcinogenic, or radioactive [3, 4]. HCW is highly heterogeneous; plastics of different grades constitute around 35–40% of total waste, with textiles (10–20%), paper and cardboard (10–15%), glass waste (4–7%), and sharps (1–3%). The hazardous fraction of HCW includes sharps, infectious, pathological waste, pharmaceutical, chemical, cytotoxic, and radioactive waste, all requiring special segregation, management, treatment, and disposal due to their significant threat to the environment and humans if disposed of otherwise [4]. In addition to the heterogeneity, the generation of HCW directly depends on population, different socioeconomic parameters, as well as on the scale and specialization of health‐care services provided, and the proportion of single‐use devices among the medical equipment used [4, 5]. In developed countries like the United States and Canada, the HCW generation is 8.4 kg and 8.2 kg/bed/day, respectively, whereas the developing nations generate 0.1–2.6 kg/bed/day of HCW [3, 6]. The wide use of single‐use disposable medical equipment to prevent infections and innovations in diagnostic and therapeutic methods also increases the generation of HCW [5] and as stated by Kaposi et al. [7] the extent of HCW continues to grow by about 2–3% annually, further intensified by the effects of COVID‐19 outbreaks.

Improper management of HCW poses a growing environmental and public health concern, particularly in the developing countries. Globally, only one‐third of healthcare centers offer adequate waste management services, and this is even worse in low and middle‐income countries, where three out of four centers do not provide such services [3, 4]. The situation was further worsened by the COVID‐19 pandemic, leading to a surge in global HCW, with developing countries experiencing increases of up to 425% [8]. Apart from the amount of HCW produced, the risks associated with improper handling of HCW are very severe. Open burning and low‐temperature incineration of HCW generate carcinogens, such as dioxins, furans, and particulates, into the air, and burying HCW in landfills leads to hazardous leachates that pollute groundwater and soil [1, 3]. Lack of adequate legislation and regulation, ineffective waste segregation practices, poor technical knowledge, and limited financial resources pose barriers to proper HCW management in developing countries [9]. Thus, it is no surprise that only 44% of hospitals in the Southeast Asian region practice segregation, safe disposal, and appropriate treatment methods for HCWs [9].

Bangladesh, as one of the world's most densely populated nations with a rapidly expanding healthcare infrastructure, faces an increasing challenge in HCW management. The HCW generation rate in Bangladesh is reported to vary from 1 to 2.6 kg/bed/day, with the maximum amount being produced in Dhaka, with a growth rate of 3% per year [3]. The megacity Dhaka also holds almost all of the country's most specialized hospitals and thus is burdened with a large amount of HCW, approximately 50 tons/day, and 26% of it is hazardous waste, which mostly (79% of the total HCW) ends up in the landfill without any treatment and mixes with the MSW [1, 9]. PRISM Bangladesh, the principal HCW processing and treatment facility in Dhaka, collects partially segregated HCW from healthcare facilities and uses chemical disinfection, autoclaving, incineration, and landfilling to manage them. The facility doesn't have any energy recovery scheme, and only 10% of the disinfected plastics are recycled. However, their capacity is lower and inadequate for sustainable energy and materials recovery for HCW with proper environmental risk mitigation [1, 3]. Such a linear approach of HCW management fails to leverage the opportunity for material recovery [3] and also emits large quantities of greenhouse gases due to methane emissions, leading to soil degradation, and facilitates the contamination of groundwater sources through leachate migration [4].

Given the nexus between rising healthcare waste production, energy shortages, and increased environmental stress in Bangladesh, a combination of energy efficiency, resource recovery, and plastic recycling in the HCWs management system is thus highly sought. Adopting a waste‐to‐energy (WtE) scheme in HCW treatment facilities, although it could be on a smaller scale to fuel its own energy requirement in powering and autoclaving, can contribute to Bangladesh's drive to energy independence and push for renewable energy sources [10]. This is significant, as the current contribution of renewable sources to the national energy supply is only about 4.6%, and Bangladesh aims to increase it to 30% by 2041 through Renewable Energy Policy, 2025 [11]. Energy‐efficient incineration, which is already known to be effective at reducing greenhouse gas emissions and land requirements compared to conventional incineration, provides another way to improve HCWs treatment [12]. Lastly, but no less importantly, there is the question of plastic recovery. As of now, about 35 percent of HCWs consist of plastic waste, which, after sterilization, is recyclable mechanically [13]. A life‐cycle assessment of medical device plastic packaging in South Korea in 2024 showed that shifting from incineration of plastics to separate mechanical recycling contributed to a decrease in the overall emissions of greenhouse gases within the sector by 9%, as burning of plastics (Incineration) contributed to doubling its environmental burden [14]. A comparison of pyrolysis to incineration has also been made specifically in relation to plastic waste generated through health care applications. Through a life cycle analysis of dialysis plastic waste from Italy performed in 2026, it was found that pyrolysis with 10 percent residual incineration generated a climate credit of −0.230 kg CO_2_e/kg of waste treated versus +1.996 kg CO_2_e/kg of waste under incineration [15]. Yet in Bangladesh, the current recycling rate at PRISM stands below 10% of recoverable plastic, representing a massive loss of secondary material value and a direct contributor to microplastic pollution through landfill disposal [3].

In addition to energy and material recovery, to ensure sustainable and circular economy (CE) based HCW management in Bangladesh, it is necessary to apply a systems‐level analysis to assess the environmental implications of different treatment options, including incineration, autoclaving, chemical disinfection, wastewater treatment, and landfilling. Thus, comparative Life cycle assessment (LCA) is required for understanding the benefits of the proposed scenarios, given that LCA assesses the environmental impacts associated with each phase of a product's lifespan, including its extraction, manufacturing, transport, use, and disposal [3]. Material flow analysis (MFA) tracks quantitative flows of waste from its generation site to its destination and serves as a supplement to LCA, in addition to its role in identifying the dominant waste streams, treatment pathways, and resource recovery opportunities. Thus, the conjecture from the LCA‐MFA approach becomes a robust decision support mechanism in shifting HCW management toward the circular economy [16]. HCWs Valorization Alternatives LCA in Latvia in 2025 showed that while energy recovery was the most favorable in terms of environmental impact, material recycling into construction materials and consumer goods like reinforced asphalt, reinforced cement, refuse‐derived fuel, syngas, acoustic tiles, and hydroponic systems was more consistent with the principles of the circular economy, and landfilling always proved to have the worst results in all scenarios considered [17].

The use of LCA for HCW treatment has seen substantial increases over the past few decades, mostly carried out on the comparison between incineration and non‐incineration methods for the treatment of HCWs, and has identified the environmental benefits associated with energy recovery from medical waste incinerators [18]. The LCA‐based approach for HCW disinfection processes, as a sole unit of treatment, including steam sterilization and chemical disinfection were also analyzed [19]. Soares et al. (2013) [20] provided one of the more comprehensive analyses of LCA studies, which compared the environmental performance of microwave disinfection, autoclave disinfection, and lime disinfection technologies, finding the former to be most eco‐efficient for small volume HCW generators. Studies conducted in Pakistan and China found that HCW treatment via incineration, energy recovery, and segregation performed much better than conventional methods across all categories [18, 21]. Nematollahi et al. (2024) [22] developed mathematical models for infectious and non‐infectious medical waste in the city of Isfahan in Iran and found that chemical disinfection of infectious waste produced significantly lower global warming potential (GWP) than pyrolysis did (859.9 vs. 1483.2 kg CO_2_ eq/ton), and composting with material recovery reduced ecotoxicity for non‐infectious waste. However, the scenario that had the most incineration (70%) performed better than landfilling or composting scenarios in other categories, it exhibited higher levels of ecotoxicity than the scenario with 60% incineration [22]. The cause of this increased toxicity was cadmium and nickel that were emitted through the incineration by‐products and this finding highlighted the importance of incinerator waste management strategy [22]. Specifically, Deepak et al. (2022) [12] performed a pioneering research on the environmental impacts of waste management in the Garhwal division of Uttarakhand using LCA by means of SimaPro 9.1.0.4 and assessing the impacts based on the CML‐IA method, wherein the comparison was made between the base scenario of waste incineration and the innovative scenario integrating energy recovery, autoclave‐shredder, chemical disinfection, and effluent treatment processes. The results showed that the innovative scenario performed better than the base one in all 11 impact categories, and the greatest improvements were achieved for global warming potential (9.72%), human toxicity (10.85%), eutrophication (11.38%), freshwater aquatic ecotoxicity (12.54%), and marine aquatic ecotoxicity (13.31%). In recent times, a comparative LCA study of Istanbul's medical waste system conducted an analysis of five competitive scenarios and found that scenarios involving increased incineration had a greater impact on the environment than scenarios involving waste segregation and reduction because of the excess bottom ash, specifically in the category of freshwater aquatic toxicity and human health toxicity [23]. However, the limitations of these studies lie in their geographic focus on specific divisions and their use of the functional unit of 1 ton of waste. The study in Istanbul is comparable to Dhaka as a megacity, but they selected only six impact categories to compare and did not show any sensitivity analysis to prove the robustness of their findings [23]. Moreover, the studies did not consider performing the LCA based on total waste generation across an entire city or impact of the increased recycling of plastic materials, which are important in urban settings like Dhaka. Furthermore, existing studies have focused on electricity production from waste, overlooking the opportunity of proposing a heat recovery scheme. While these studies propose several alternative scenarios for sustainable HCWs management, the linkage between Sustainable Development Goals (SDGs) and environment‐friendly schemes has received less attention explicitly. Bangladesh has limited thorough studies on HCW management, while most focus only on quantifying and classifying HCWs in single regional hospitals. Alam and Mosharraf (2020) [24] conducted a preliminary LCA in Chittagong city to examine global warming potential, human toxicity, freshwater ecotoxicity, and terrestrial ecotoxicity of HCWs across three scenarios. Dihan et al. (2023) [3] subsequently provided a comprehensive review of the HCW generation scenario, COVID‐19 impacts, and CE frameworks applicable to Bangladesh, establishing the need for quantitative LCA‐based sustainability assessment but stopping short of conducting an integrated LCA. A comparative review of recent literature on HCWs‐related LCA has been provided in the Supporting Information document (Table S1). However, an integrated MFA‐LCA study of HCWs management in Dhaka city has not yet been conducted. The lack of such studies poses a major barrier to the formation of evidence‐based policies for sustainable HCW management in Bangladesh. The addition of an integrated WtE scheme for energy (both heat and electricity) recovery, waste plastic characterization, and enhanced recycling to adapt the CE framework for HCW management in Bangladesh can provide a significant advancement in the country's policy‐making for sustainable HCW management.

Given the foregoing discussion, in this article, we propose, for the first time in Bangladesh, a sustainable HCW management facility that integrates a WtE and enhanced plastic recycling scheme with current treatment practices and a holistic MFA‐LCA approach to analyze the associated environmental benefits. To do so, we first calculate the total medical waste generation in a day in Dhaka City using empirical equations and collected number of hospital beds and based on that functional unit, we conduct the LCA. In total, eighteen impact categories, acidification potential, climate change, freshwater ecotoxicity, marine ecotoxicity, terrestrial ecotoxicity, nonrenewable fossil fuel potential, freshwater eutrophication, marine eutrophication, human toxicity: carcinogenic, human toxicity: non‐carcinogenic, ionising radiation potential, minerals resources, ozone depletion, land use potential, particulate matter formation, water consumption potential, photochemical oxidant formation: humans and photochemical oxidant formation: ecosystems are assessed for current and proposed scenarios. In addition, the hotspot analysis identifies the major environmental contributors in the existing and proposed schemes, as well as potential areas for future improvements. Furthermore, the sensitivity analysis is conducted for SC2 relative to SC1 based on five key parameters to assess the robustness of comparative results. Finally, the associated Sustainable Development Goals (SDGs) are linked to the proposed schemes to address the existing gap between HCW management and sustainability. We believe this study will enable policy‐making to shift from the currently practiced linear HCW management to a circular‐economy‐based approach that prioritizes energy and resource recovery as well as appropriate residue treatment while serving as a starting point for further research to achieve sustainability in HCW management in Bangladesh.

## 2 Materials and Methodology

### 2.1 Data Collection, Source, and Facility Location

Multiple field visits were conducted at PRISM Bangladesh in Dhaka to collect primary data for the study. Detailed information on waste quantity, segregation practices, incineration, autoclaving, chemical disinfection, shredding, and recycling was collected. In addition, the quantity of plastic waste, the polymer type, and the recovery method were collected from the published literature. From the PRISM, HCW plastic samples, including syringes, tubes, catheters, ampoules, and incinerator bottom ash, were collected and analyzed to classify the generated plastic wastes. The PRISM approach for medical waste treatment which constructs our baseline scenario (SC1) is provided in Figure S1. The other data obtained from PRISM and the literature are presented in Section S3 (Field Survey and literature data) in Figure S3, Tables S8 and S9.

To quantify the HCW generation rate in Dhaka city, the following empirical linear regression models (Equations (1), (2), (3)) were used. These models were developed by Minoglou et al., based on four specific socioeconomic parameters [6].

(1) MWGR=0.014×LE+0.31×CE

(2) MWGR=1.5×HDI+0.29×CE

(3) MWGR=0.13×MYS+0.278×CE

where MWGR = medical waste generation rate (kg/bed/day), LE = Life expectancy (years), CE = CO_2_ emission (Tons/per capita/year), HDI = Human Development Index, and MYS = mean years of schooling (years).

To characterize and compute the composition of the HCW plastics, following ASTM D6044 for heterogeneous waste, a random sample of recyclable, non‐infectious HCW was collected from the PRISM facility. The collected sample was segregated and analyzed manually to determine the type of plastic present in the sample, following

(4) $$\begin{array}{ccc} & & \mathrm{Plastic}\;\mathrm{wt}.\;\mathrm{percentage}\\ & & =\frac{\mathrm{Measured}\;\mathrm{weight}\;\mathrm{of}\;\mathrm{the}\;\mathrm{particular}\;\mathrm{plastic}}{\mathrm{Total}\;\mathrm{weight}\;\mathrm{of}\;\mathrm{the}\;\mathrm{collected}\;\mathrm{sample}}\times 100\% \end{array}$$

The maximum recyclable quantity was measured (Equation 5) using the reprocessing potential [25] (Table S10), where the maximum recyclable plastic quantity = Quantity of particular plastic × reprocessing potential of that plastic.

(5) $$\begin{array}{ccc} & & \mathrm{Maximum}\;\mathrm{recyclable}\;\mathrm{percentage}\\ & & =\frac{\sum \;\mathrm{Maximum}\;\mathrm{recyclable}\;\mathrm{plastic}\;\mathrm{of}\;\mathrm{each}\;\mathrm{type}}{\mathrm{Total}\;\mathrm{collected}\;\mathrm{plastic}\;\mathrm{weight}}\times 100\% \end{array}$$

### 2.2 Material Flow Analysis (MFA)

Material Flow Analysis (MFA) is used to quantify and track the generation, transportation, transformation, and endpoint of materials within the system boundary for a specified period. MFA is particularly important for waste management studies for identifying dominant waste streams, treatment pathways, and resource recovery opportunities [16]. For this study, a material flow analysis was conducted to map the physical pathways of medical waste from healthcare facilities to its final treatment or disposal. Two separate MFA diagrams were developed to represent (i) the current overall medical waste flow in Dhaka city and (ii) the current waste treatment operations carried out by PRISM (the base case). MFA was established by combining field survey data with characterization data from the literature, and the information is demonstrated using process block diagrams in Section S6 of the Supporting Information (Figures S6 and S5). This MFA approach provides a quantitative representation through the Sankey diagram to identify key waste streams, treatment paths, and material recovery options, and ensures that the LCA boundaries are properly defined and that the important waste streams are accurately included in the assessment.

### 2.3 Life Cycle Assessment (LCA)

OpenLCA software (v2.6.2) and the Ecoinvent v3.9.1 database (cut‐off system model, unit process) were used to perform the LCA, with the ReCiPe 2016 Midpoint (H) impact assessment method. The study followed ISO 14040:2006 for establishing LCA principles and frameworks, defining goals, scope, and result interpretation, and ISO 14044:2006 standards for detailing LCA procedures, covering inventory analysis, impact assessment, and interpretation. These standards ensure consistency and transparency in conducting LCA studies.

#### 2.3.1 Goal and Scope of the Study and the Functional Unit

The goal was to evaluate the best scheme in terms of environmental performance and impacts between the current and proposed HCW schemes in Dhaka city, through a “gate‐to‐grave” approach that covers waste generation at healthcare facilities, transportation, treatment, and final disposal or recycling. The baseline scenario (SC1) represents the existing system consisting of conventional collection systems, autoclaving, chemical disinfection, and landfill disposal. Meanwhile, the alternative scenario (SC2) was developed using an integrated system of improved segregation and waste‐to‐energy (WtE) recovery from incineration for electricity and steam generation, and the steam is subsequently used for autoclaving.

The functional unit was defined by ISO 14044 as the “quantified performance of a product system for use as a reference unit” [26]. For the analysis, the functional unit was 46.32 tons of HCW, the daily generation capacity of Dhaka city.

#### 2.3.2 System Boundaries

##### 2.3.2.1 Baseline scenario (SC1)

The baseline scenario (SC1) represents the current linear waste management model in Dhaka city, in which 46.32 tons of medical waste undergo a sequential treatment process comprising segregation, landfilling, autoclaving, incineration, and chemical disinfection, as depicted in Figure 1. General waste is landfilled directly without material or energy recovery. Of the fraction collected by PRISM, infectious waste is autoclaved and pathological waste is sent directly to the incinerator. Following autoclaving, only the sterilised infectious waste is incinerated, while the residual autoclaved sharps and infectious plastics are open dumped. The non‐infectious plastic fraction undergoes chemical disinfection, after which only the recyclable plastics are shredded and recycled, and the remaining non‐recyclable plastics are open dumped. The thermal energy from the high‐temperature flue gas remains unutilized, and the incineration residue is landfilled without cement stabilization or other pretreatment. Wastewater generated from these processes is treated at the Effluent Treatment Plant (ETP) to meet regulatory discharge standards. Ancillary processes also include a boiler, employed to generate the steam required for autoclaving sterilized waste. A detailed process block diagram of the baseline system, including all intermediate flows, is provided in Figure S5.

**FIGURE 1:**
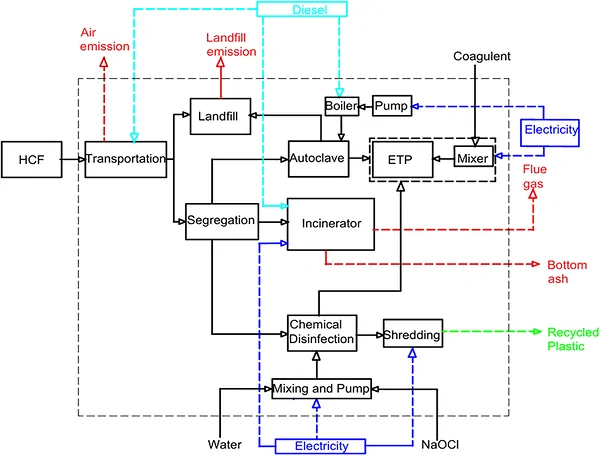
The currently practiced linear HCW management scenario in Dhaka city, base scenario (SC1).

##### 2.3.2.2 Alternative Circular economy scenario (SC2)

The alternative scenario (SC2) introduces an integrated waste management system that optimizes resource recovery and energy utilization, shifting from the linear model of SC1. This scenario considers the maximized recycling potential of non‐infectious plastic waste. Moreover, in this scheme (Figure 2), the compostable general waste is co‐incinerated with non‐recyclable hazardous HCW in a single incinerator. The thermal energy from the flue gas of this incinerator is used in a waste‐to‐energy (WtE) cycle, generating steam for the autoclave and producing electricity. The high‐temperature flue gas is first used to generate superheated steam through a steam producer. The exhaust flue gas from a controlled point source then leaves the system boundary through a scrubber, decreasing the CO_2_ and SO_x_ emissions. Superheated steam is used to generate electricity in steam turbines, and the low‐pressure steam is used in the autoclave, eliminating the need for a boiler in the SC1. Non‐recyclable residual plastic arising from autoclaving and chemical disinfection is retained on the same open‐dumping pathway as in SC1. The incineration residue is stabilized with cement before landfilling to improve leachate management. The corresponding process block diagram for the alternative scenario is given in Figure S6.

**FIGURE 2:**
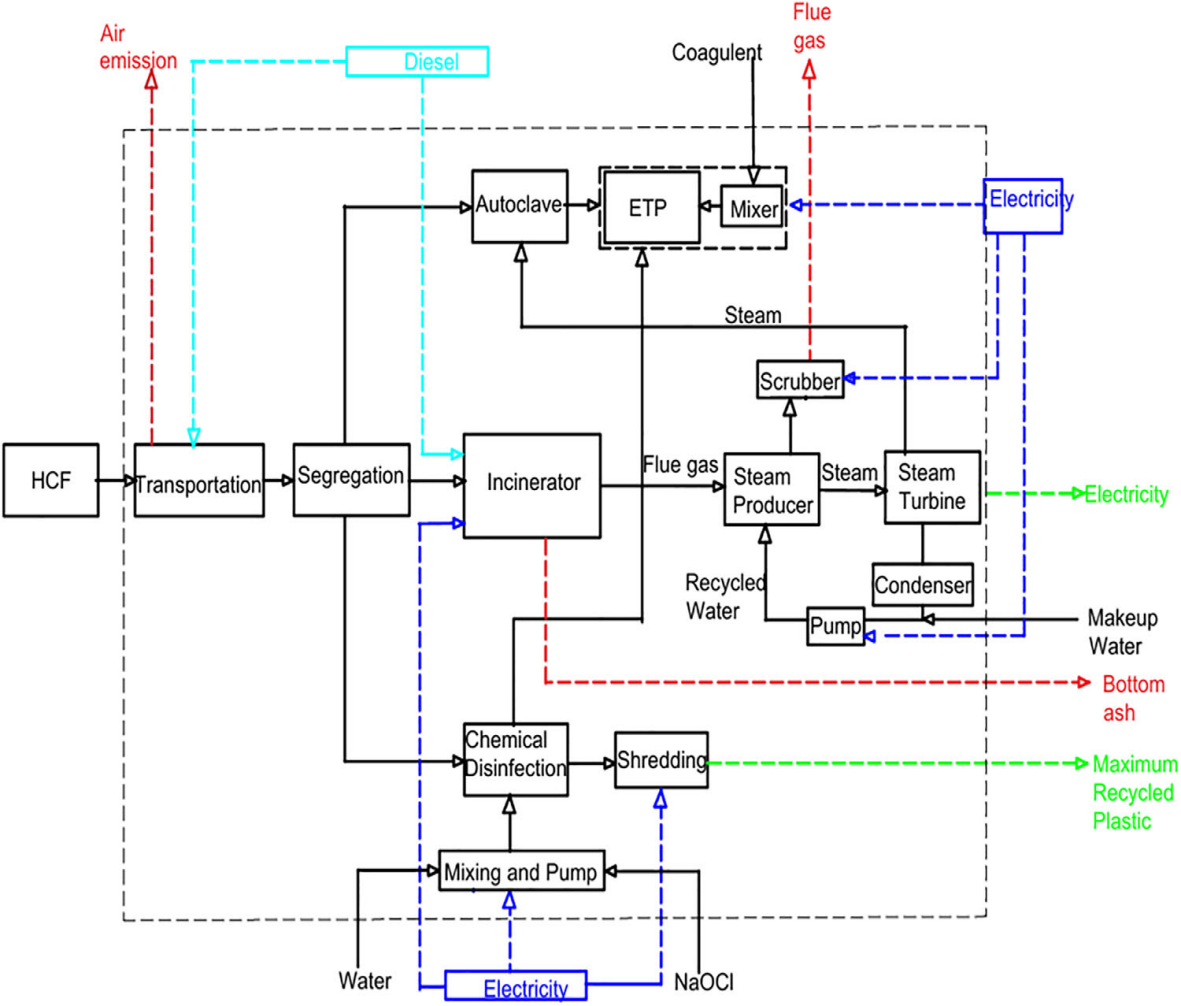
The system boundary for the proposed integrated energy and resource recovery scheme (SC2) for HCW management.

The life cycle inventory (LCI) data supporting this study are provided in Section S2, in Tables S2–S7 and Figure S2. Detailed calculations of electricity recovery in SC2 are provided in sub section S4.2 (Table S13), while the estimation of methane generation from landfill is detailed in sub section S4.3, with the corresponding calculations presented in Tables S14 and S15 and Figure S4.

#### 2.3.3 Life Cycle Impact Assessment and Interpretation

The life cycle impact assessment (LCIA) evaluated the environmental impacts of the proposed HCW management system by converting life cycle inventory results into defined impact categories. The inventory analysis can be grouped into midpoint and endpoint damages. Midpoint indicators assess environmental impacts earlier in the cause–effect chain using specific impact categories, whereas endpoint indicators aggregate multiple midpoint impacts to evaluate their overall damage to areas such as human health, ecosystems, and resources [27]. The midpoint impact categories were chosen, since they help to keep track of process‐level specifics and provide better associations between emissions and their environmental impacts. The ReCiPe 2016 Midpoint (Hierarchist) method was used in openLCA v2.6.2 software, which utilizes the Ecoinvent v3.9.1 database, to assess 18 midpoint categories of environmental impacts including climate change, acidification, eutrophication, toxicity, resource, land use, ozone depletion, particulate matter, photochemical oxidant formation, and water consumption. Process level contribution trees were used in the interpretation stage to identify hotspots and ReCiPe 2016 World (2010) H normalization to rank the impact categories based on the relative significance.

A one‐factor‐at‐a‐time sensitivity analysis was performed to test the reliability of the results obtained in the comparison of the base case and alternative cases. Five critical parameters selected using hotspot analysis, gross electricity recovery, amount of incineration residue, NaOCl amount, plastic recycling amount, and transportation distance, were varied to find their effect on all 18 impact categories. In general, all parameters were varied in the range of ±20%, except for plastic recycling, which was varied to be decreased by 20% and 40% since it is already at the maximum value in the alternative scenario. This approach enabled the key parameters that influenced each impact category to be identified and verified whether the comparative results were consistent throughout the range tested, enabling the foundation of the limitations and recommendations presented in the concluding sections of this paper.

## 3 Results and Discussions

### 3.1 Estimation of HCW Generation From the Empirical Model

The three empirical models (Equations (1), (2), (3)) using socioeconomic parameters for Bangladesh projected increases in the HCW generation rate over the last 15 years. In 2025, the estimated generation rates are 1.25, 1.22, and 1.06 kg/bed/day for models 1, 2, and 3, respectively (Figure 3; Table S11). The lowest growth rate of 15.6%, estimated by Model 1, is attributable to the comparatively lower increase in life expectancy at birth (LE) of 6.5%, even though this model predicted a substantial growth (99%) in CO_2_ emissions per capita. The results reflect an improved, but inherently waste‐intensive, healthcare infrastructure compared to models 2 and 3. These models demonstrated a ∼2x growth rate compared to model 1, with 30.5% and 35.3%, respectively, suggesting that socio‐economic development (HDI), education‐driven healthcare awareness (MYS), and advanced medical apparatus and disposables produced by industries (CE) synergistically impact MWGR. The outputs also indicate that medical waste generation is shaped not only by bed capacity but also by demographic transition, technological intensity, and consumption behavior.

**FIGURE 3:**
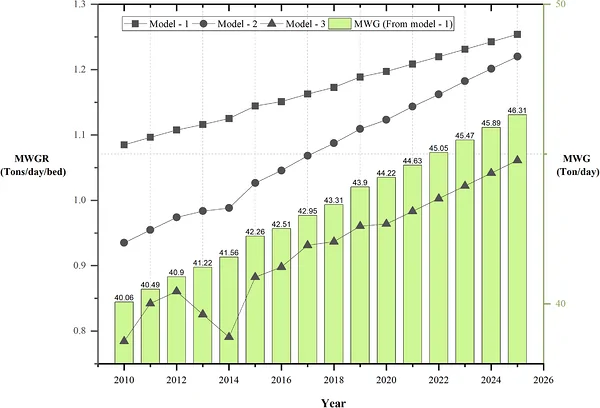
Estimated medical waste generation rate (kg/bed/day) from 2010 to 2025 from three empirical regression models (Eq‐1 to Eq‐3). The bar plots show the predicted HCW (tons/day) for the model Equation (1).

Based on the predictions, Dhaka city is estimated to generate 46.32, 45.05, and 39.18 tons/day in 2025 across 36,933 hospital beds in public and private healthcare facilities under DNCC and DSCC (Table S12). However, the field visit in PRISM confirmed that the actual HCW generation exceeds 45–50 tons/day, with approximately 80% as general waste and 20% as hazardous waste, aligning with the projections from model equation 1. Therefore, the estimated HCW generation based on model Equation 1 (46.32 tons/day) was selected as the functional unit for the LCA. The close agreement between the projected HCW generation rate and the factual data from PRISM strengthens the reliability of the inventory basis and provides an optimum platform for environmental analysis for real‐life operational conditions.

### 3.2 Material and Energy Flow Analysis of the HCW

The detailed Material Flow Analysis (MFA) in Figures 4 and 5 provided a quantitative assessment of the generation, treatment, and final deposition of HCW in Dhaka. 79% (36.6 tons/day) of the total 46.32 tons of medical waste generated per day were classified as general waste and subjected to direct landfilling, as illustrated in Figure 4, whereas only 21% undergo formal treatment through PRISM. This is consistent with the common scenario of the developing countries, where landfill dumping accounts for >70% of the waste generated [28]. In addition, 72.5% of total plastic waste and 50.4% of textile waste are dumped in landfills, potentially leading to microplastic generation and hazardous leachate formation [29]. Though the hazardous waste undergoes various treatment methods such as autoclaving, chemical disinfection, incineration, and shredding, approximately 37.9% of the hazardous waste (0.94 tons) was sent to open dumping, along with 2.024 tons of non‐recyclable plastic waste. Separately, 1.1 tons of residue after incineration was disposed of in landfill without pre‐treatment (Figure 5). Together, in addition to the 36.6 tons of general waste, a total amount of 40.7 tons/day, or 88% of the total produced waste, was destined for landfills or open dumping. The PRISM process flow block diagram for Scenario SC1 is provided in Section S6, Figure S5. This scenario indicates that the current approach to waste treatment is highly dependent upon landfilling at each stage of waste processing and less on energy or material recovery. The MFA further highlights the critically low rate of plastic recycling in the current scenario, with only 0.4171 tons recycled from the 3.386 tons produced daily. Polypropylene (PP) has the highest recyclability potential among plastic waste streams, accounting for 191 kg recycled daily. The low recycling rate suggests limitations in segregation, contamination control, and plastic collection systems. Energy Flow Analysis, as illustrated in Figure 6, further confirms that transportation alone was responsible for about 94.9% of total operational energy demand, using 30,876 MJ/day of energy through diesel‐driven truck collection. Taking into account diesel use at the incinerator and boiler, diesel‐driven energy sources represented 99.3% of total operational energy (32,314 MJ/day), while electricity‐driven unit processes such as shredding, incineration, autoclaving, effluent treatment, and chemical disinfection contributed a mere 0.7% (212 MJ/day) of energy to the operation. According to Figure 6, medical waste collection and logistics are the biggest energy hotspots for SC1, whereas energy requirements of all downstream processes become relatively insignificant in comparison. Energy flow analysis, combined with material flow analysis, confirms that excessive landfilling, poor material recovery, and energy‐intensive transportation are significant limitations in the baseline scenario, calling for a circular treatment approach.

**FIGURE 4:**
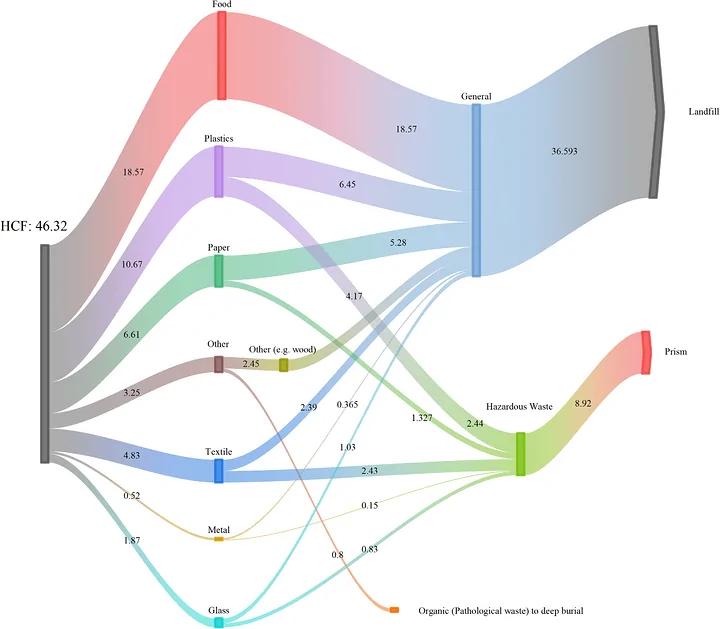
The Sankey diagram of the flow of the generated medical waste in Dhaka city and the end points in ton/day.

**FIGURE 5:**
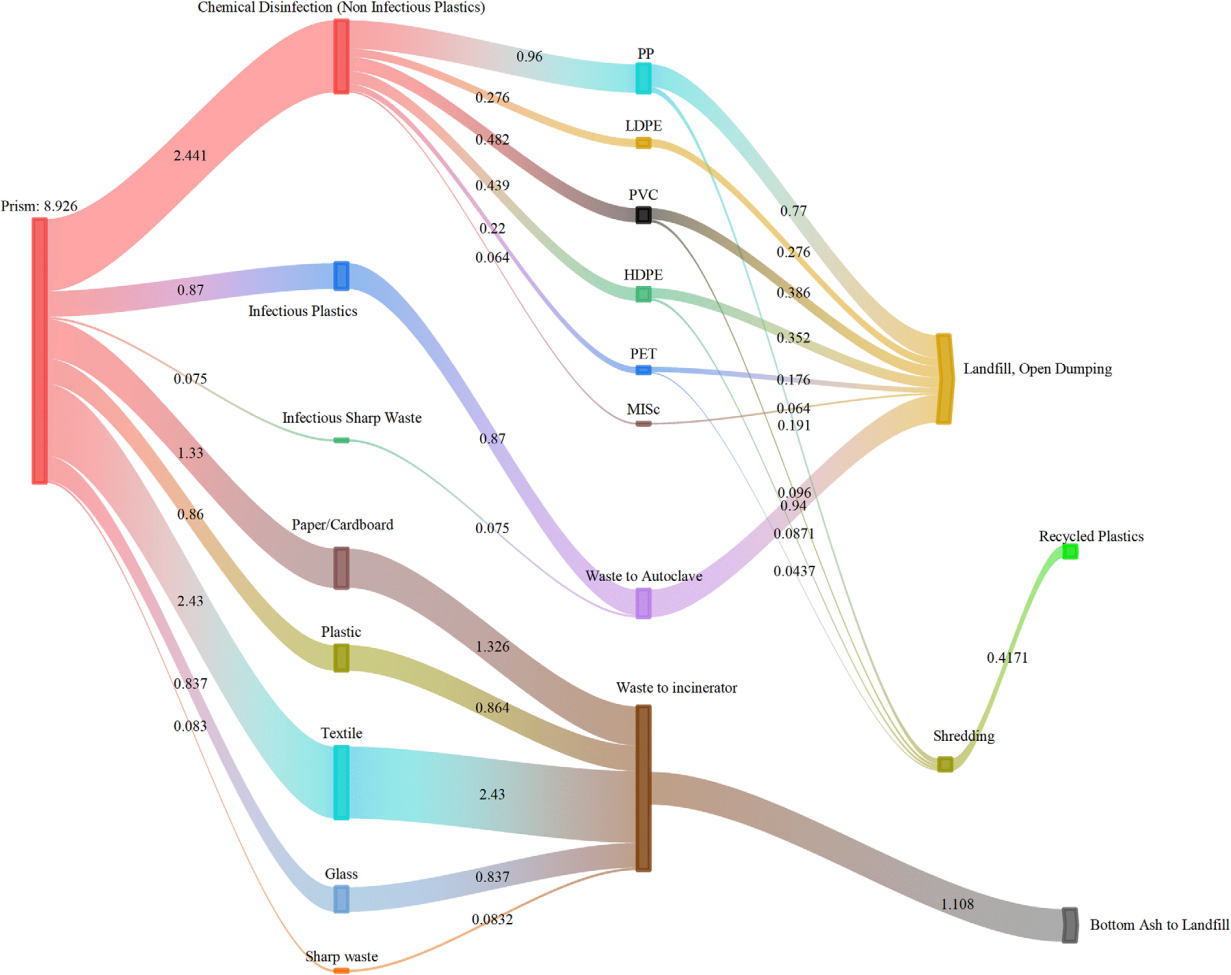
The material flow of the HCW collected by the PRISM and the endpoints of the waste in ton/day.

**FIGURE 6:**
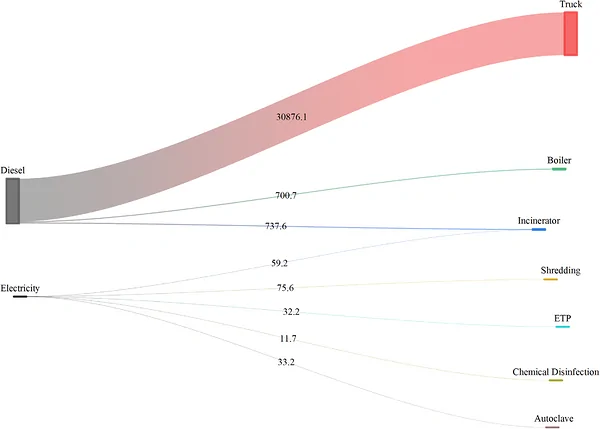
Energy consumption flows for the collected HCW treatment by PRISM in MJ/day.

### 3.3 Comparative Life‐Cycle Impact Assessment

Comparative LCA was performed to compare both scenarios, where 18 impact categories, such as: terrestrial acidification potential (TAP), climate change (GWP), freshwater ecotoxicity (FETP), marine ecotoxicity (METP), terrestrial ecotoxicity (TETP), nonrenewable fossil fuel potential (FFP), freshwater eutrophication (FEP), marine eutrophication (MEP), carcinogenic human toxicity (HTPc), non‐carcinogenic human toxicity (HTPnc), ionising radiation (IRP), land use potential (LOP), surplus ore potential (SOP), ozone depletion (ODP), particulate matter formation (PMFP), photochemical oxidant formation affecting human health (HOFP), photochemical oxidant formation affecting terrestrial ecosystems (EOFP), and water consumption (WCP) were analyzed. Table S16 depicts the comparative life‐cycle impact assessment (LCIA) results for the existing scenario (SC1) and alternative scenario (SC2). The baseline scenario recycled 417.1 kg of plastics (no electricity recovery), while the alternative scenario had an improved recycling potential of 1.53 tons/day with 20572.28 kWh of electricity recovery (Tables S10 and S13). The benefits from plastic recycling and energy recovery were modelled as avoided products, so the impacts of virgin polymer production (PET, HDPE, PVC, and PP) and grid electricity were subtracted from the treatment system's impacts. Figures 7 and 8 demonstrate the comparative LCA results for the two processes across all 18 categories. The alternative scenario performed better than the baseline in six categories, with reductions of 96.1% in TAP, 97.0% in LOP, 82.0% in IRP, 78.3% in GWP, 44.1% in MEP, and 43.1% in SOP. In four further categories (FFP, ODP, PMFP, and WCP), the credits from recycling and energy recovery exceeded the burdens of the system. These gains, however, came at the cost of a consistent deterioration across the toxicity categories. HTPc rose by 275.5%, FETP and METP by roughly 131% each, TETP by 103.8%, and HTPnc by 71.4%, while FEP nearly doubled. Photochemical oxidant formation also increased sharply, by 214.0% for HOFP and 202.5% for EOFP. The results of impact assessment can be found in Table S16. Even though all the 18 impact categories have been shown in Figures 7 and 8, discussions on all of them has been omitted and the following 13 have been discussed due to their relevance to healthcare waste management: TAP, GWP, FETP, METP, TETP, FFP, FEP, MEP, HTPc, HTPnc, LOP, PMFP, and EOFP. The existing LCA based literatures on HCW also aligns with those. Such as Deepak et al. (2022) [12] considered 11 impact categories whereas Nematollahi et al. (2024) [22] considered 10 impact categories and Çetin et al. (2025) [23] considered only six. None of them considered IRP, SOP, ODP, and WCP due to irrelevance. The last one, EOFP, has been discussed in detail, but HOFP has not been discussed separately since both have the same NO_x_ chemistry except for the exposure pathway. The rest of the 4 impact categories IRP, SOP, ODP, and WCP have not been individually discussed since they have very small magnitudes or are predominantly dominated by upstream processes.

**FIGURE 7:**
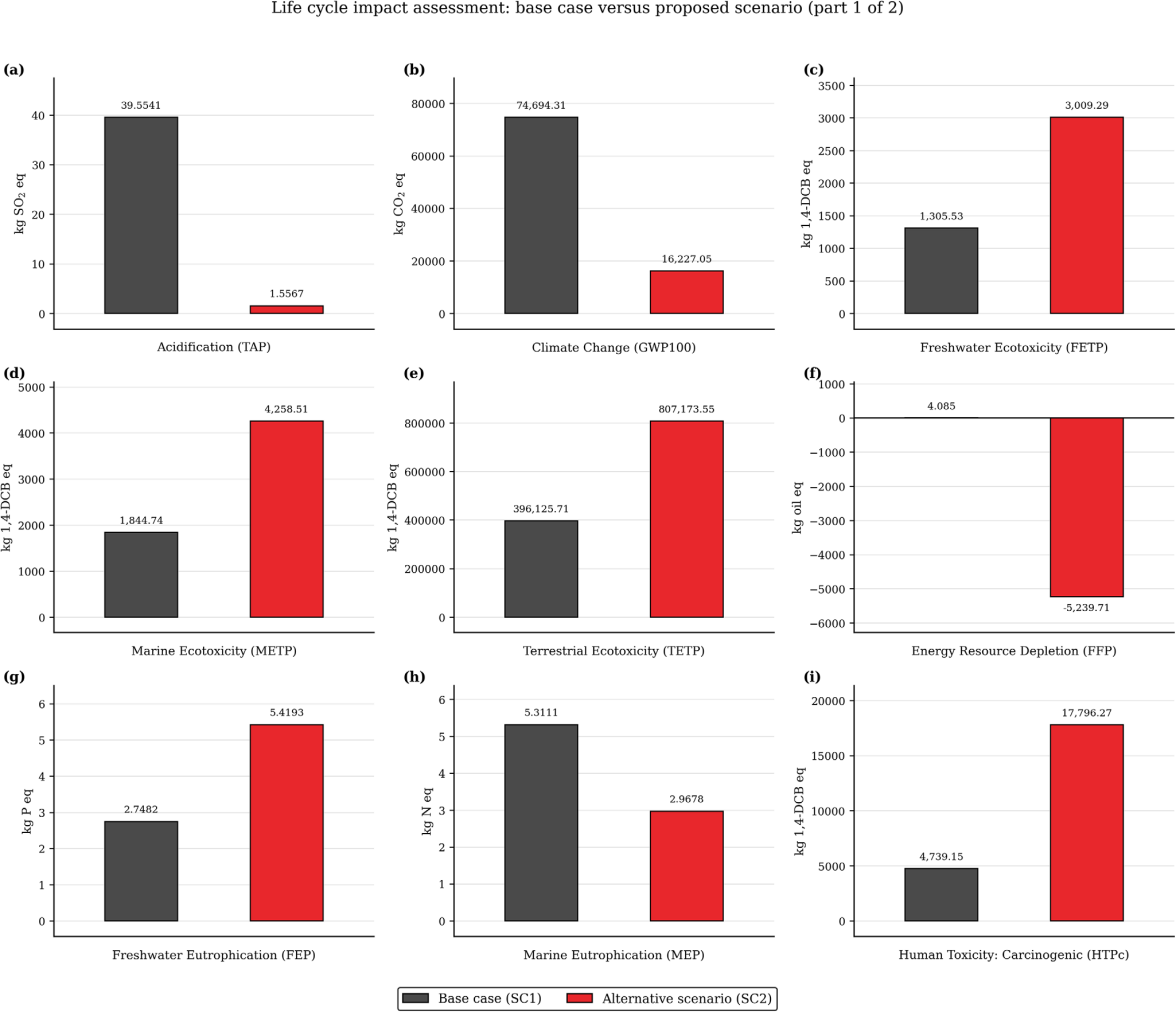
Life cycle impact assessment between the base case and the proposed scenarios for eighteen impact categories (Part 1 of 2).

**FIGURE 8:**
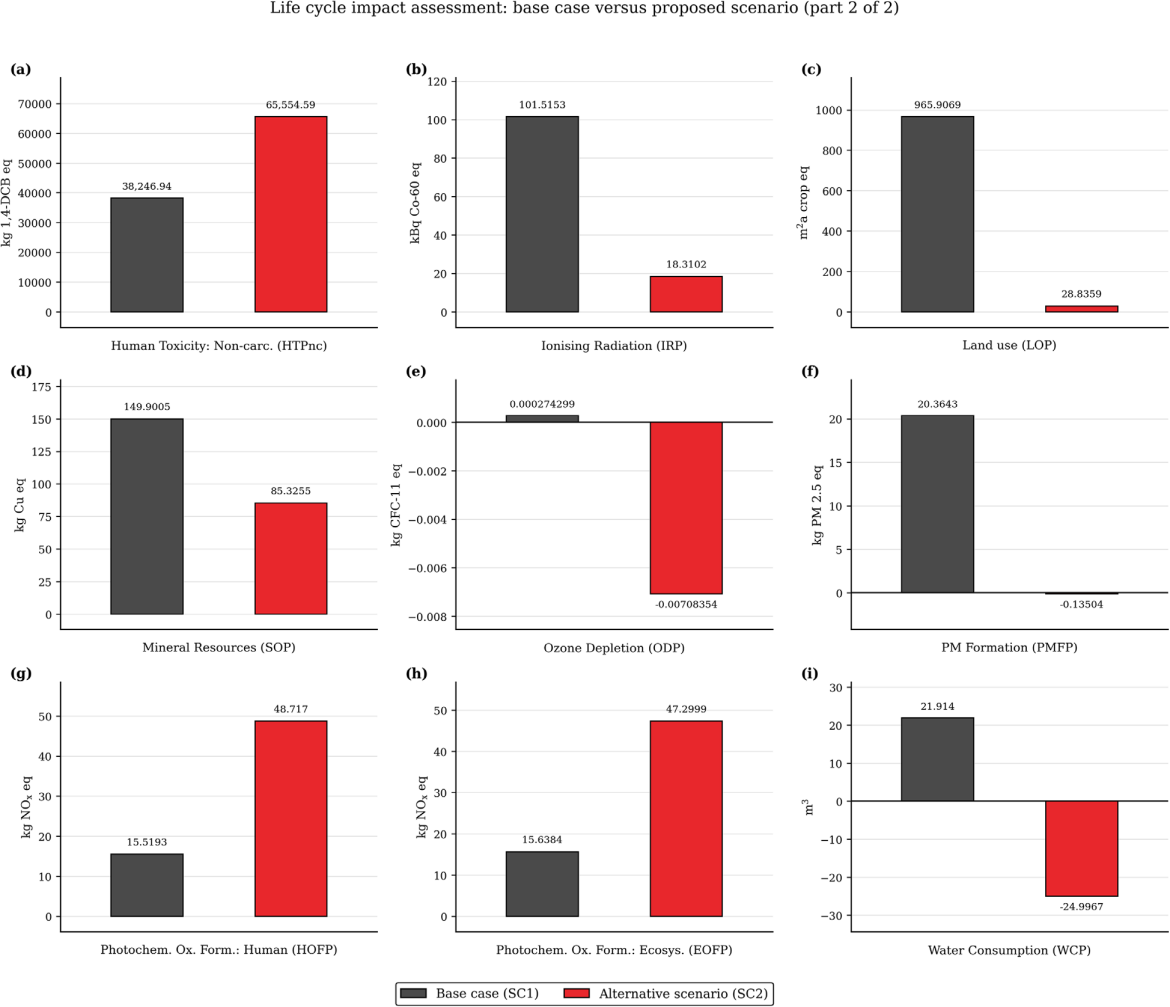
Life cycle impact assessment between the base case and the proposed scenarios for eighteen impact categories (Part 2 of 2).

### 3.4 Interpretation of the LCA Results

#### 3.4.1 Terrestrial Acidification Potential (TAP)

Acidification potential (AP), measured in kg SO_2_ eq, determines the capacity of emissions such as SO_x_, NO_x_, and NH_3_ to cause acid deposition in soil and water ecosystems [30]. TAP decreased from 39.554 kg SO_2_ eq in SC1 to 1.557 kg SO_2_ eq in SC2, a 96.1% reduction (Figure 7a). As the hotspot analysis of the two scenarios showed (Figures 9 and 10), in SC1, landfilling was the dominant source at 25.480 kg SO_2_ eq, 60% of the gross burden, originating entirely from the direct release of 13.00 kg NH_3_ (Characterization Factor, CF = 1.96 kg SO_2_‐eq/kg) during anaerobic degradation, which formed secondary ammonium salts and released H^+^ in soil. Incineration contributed 9.648 kg SO_2_ eq through stack SO_2_ (5.549 kg, CF 1.00 kg SO_2_‐eq/kg) and NO_x_ (8.984 kg, CF 0.36 kg SO_2_‐eq/kg), and chemical disinfection a further 4.902 kg SO_2_ eq located almost entirely in upstream sodium hypochlorite manufacture. Although the elimination of landfilling in SC2 removed the diffuse NH_3_ pathway completely, the increased incineration throughput raised stack NO_x_ 7.6‐fold to 68.421 kg (CF 0.36 kg SO_2_‐eq/kg) and stack SO_2_ by 44% to 7.977 kg (CF 1.00 kg SO_2_‐eq/kg) despite downstream scrubbing, making incineration the dominant source at 41.124 kg SO_2_‐eq or 85% of the gross burden. The reduction was therefore achieved primarily through the recovery credits, where energy recovery avoided 36.019 kg SO_2_ eq by displacing grid electricity and plastic recycling avoided a further 10.746 kg SO_2_ eq through displaced virgin polymer production, together offsetting 97% of the gross burden. The result consequently remained sensitive to the assumed grid mix and recovery efficiency, as discussed in Section 3.7. Deepak et al. (2022) [12] reported the acidification potential of 5.87 kg SO_2_ eq in conventional incineration and 5.39 kg SO_2_ eq in incineration as a WtE technology for the LCA of biomedical waste in Uttarakhand, India. Meanwhile, Koo et al. (2015) [31] reduced TAP by 5.4 kg SO_2_ eq by integrating incineration with heat recovery for medical waste treatment in Korea, whereas in our study, 38.00 kg SO_2_ eq AP was reduced. These deviations arose from the substantially larger functional unit adopted in this study and, more importantly, from the composition of the baseline: neither study assessed a landfill‐dominated system, so the NH_3_ pathway that governs SC1 was absent from their reference cases, and the achievable reduction was correspondingly smaller. Khatun et al. (2025) [32] also reported reductions in acidification ranging from 88% to 98.5% when transitioning from conventional open systems to controlled biogas‐energy systems, a range consistent with the 96.1% obtained here. Nevertheless, the synergistic effects of eliminating landfilling and integrating WtE recovery in SC2 are reflected in the net reduced acidification potential. Although the gross burden of SC2 (48.321 kg SO_2_ eq) slightly exceeded that of SC1 (42.483 kg SO_2_ eq), it originated from a single point source rather than the diffused landfill NH_3_ pathway (25.480 kg SO_2_ eq) that dominated SC1, making it amenable to abatement through SCR or SNCR for NO_x_ control.

**FIGURE 9:**
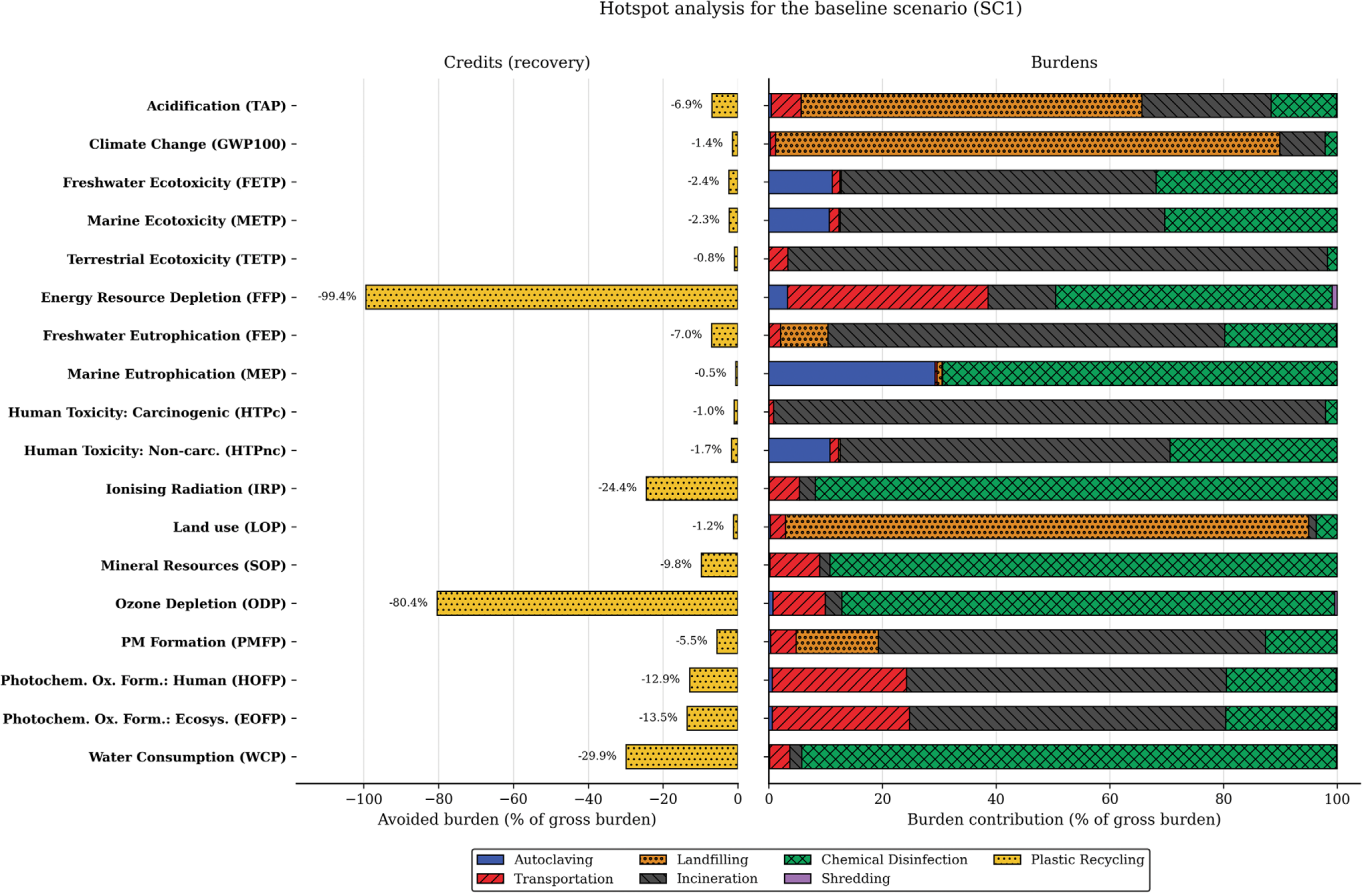
Hotspot analysis for the currently practiced management scenario (SC1).

**FIGURE 10:**
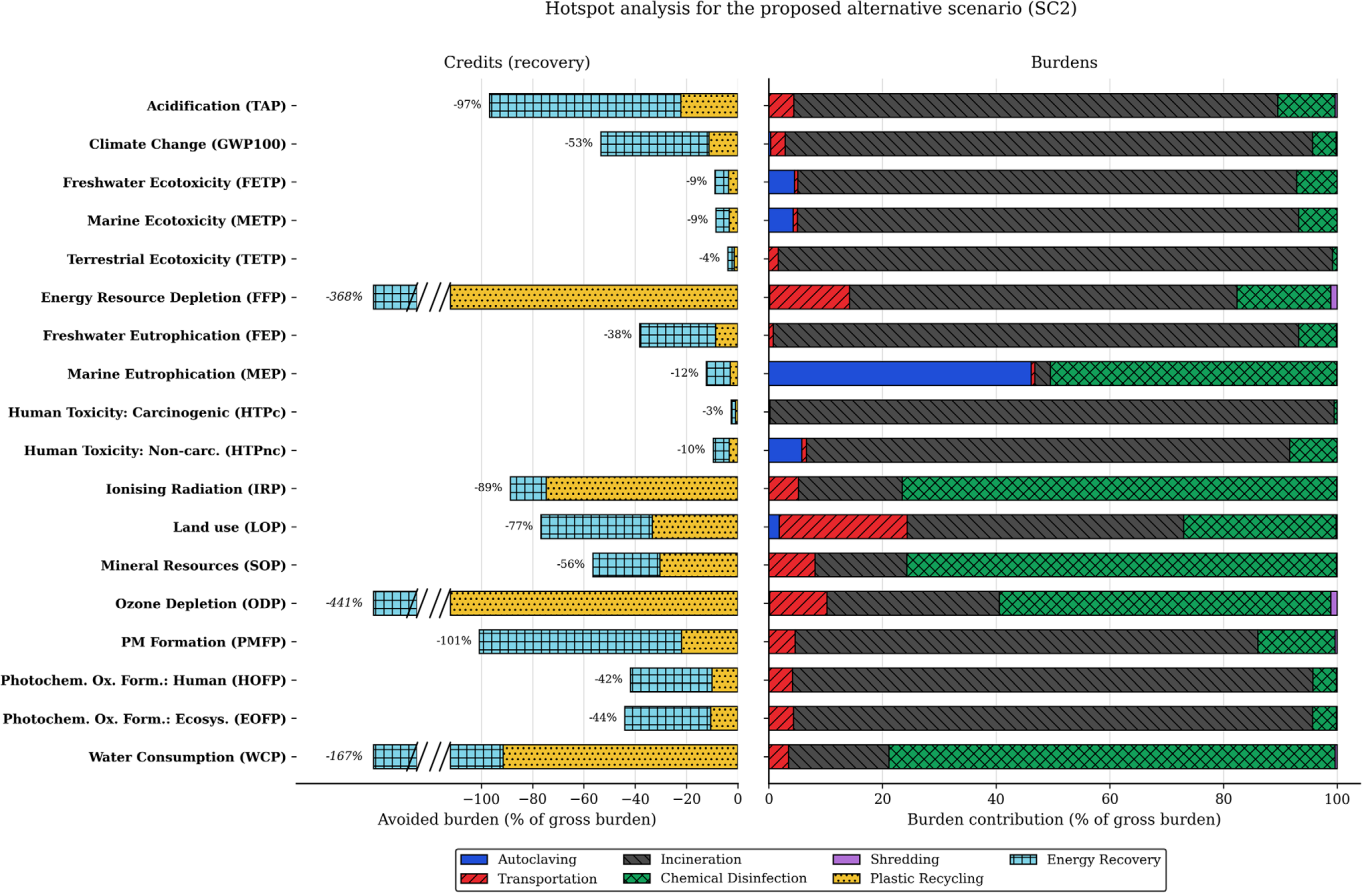
Hotspot analysis for the proposed alternative scenario (SC2).

#### 3.4.2 Climate Change (GWP100)

Global warming potential (GWP) is defined as the relative capacity of a greenhouse gas to retain heat in the atmosphere relative to an equivalent amount of kg CO_2_‐eq. It accounts for both gas's heat‐trapping ability and how long it remains in the atmosphere [33]. The climate change impact, as illustrated in Figure 7b, showed a significant reduction by 78.28% from 74 694.31 kg CO_2_‐eq in SC1 to 16 227.01 kg CO_2_‐eq in SC2, mainly attributable to the exclusion of methane emissions from landfilling. In SC1, the greenhouse gas contribution from landfilling was 67,173.80 kg CO_2_‐eq (≈89% of the gross burden), through the process of anaerobic decomposition (1,975.70 kg CH_4_, CF = 34 kg CO_2_‐eq/kg). This was mainly because, compared to CO_2_, CH_4_ has a very high characterization factor and thus contributed most to GWP in this case. However, the removal of the diffuse methane pathway and replacement of the climate burden by controlled incineration in SC2 meant that carbon emissions were dominated by fossil/nonfossil CO_2_ in incineration. Consequently, the gross burden of 34,808.40 kg CO_2_‐eq in SC2 was made up of mainly by direct fossil CO_2_ emission from incineration (26 704.6 kg CO_2_‐eq), where energy recovery and plastic recycling providing significant offsets of −14 675.00 kg CO_2_‐eq and −3906.36 kg CO_2_‐eq, respectively, resulting in the reported net GWP (Figure 10). A similar result was reported by Deepak et al. (2022) [12] where 1094.28 kg CO_2_ eq was reduced in an incineration‐based WtE system for biomedical waste management. Their calculated value was less than this paper because of their choice of functional unit of 2.547‐ton waste, and they did not consider to increase plastic recycling in their LCA scenario. However, the trend was same as controlled incineration with energy recovery decreased the global warming potential over their baseline scenario. Rahman et al. (2024) [34] eliminated methane emissions, which resulted in reduced climate change impacts by 7.7 × 10^6^ kg CO_2_ eq in the HTC‐based WtE model compared to landfilling. Comparable observations were also reported in Khatun et al. (2025) [32] by integrating controlled systems for improved gas management to achieve 24.74–53.32% reductions in GWP relative to conventional practices. The results suggest that the combined impacts of CH_4_ mitigation and controlled combustion lead to a radical decrease in climate change impacts in SC2, underscoring the effectiveness of integrated waste‐to‐energy configurations.

#### 3.4.3 Freshwater Ecotoxicity Potential (FETP)

Freshwater Ecotoxicity Potential (FETP) evaluates the potential adverse impacts of toxic substances released into freshwater environments and is commonly measured in kg 1,4‐dichlorobenzene equivalents (1,4‐DCB‐eq) [35]. Heavy metals such as zinc (Zn) and copper (Cu) are major contributors to freshwater ecotoxicity [35]. The FETP increased from 1305.53 kg 1,4‐DCB‐eq in SC1 to 3009.29 kg 1,4‐DCB‐eq in SC2, corresponding to an approximately 130.5% increase (Figure 7c) in SC2. This increase was consistent with the stronger influence of heavy‐metal‐related releases associated with incinerator bottom ash generated under higher incineration throughput. For SC1, the share of incineration was 55% for the gross burden, where the metal burden was contributed mainly by leaching of the incineration residues from landfill sites due to antimony (0.73 kg to groundwater; CF = 340 kg 1,4‐DCB‐eq/kg) and zinc (0.98 kg; CF = 211 kg 1,4‐DCB‐eq/kg). For SC2, again, this route scaled along the throughput, with 88% being the share of the incineration for the burden, antimony being 2.818 kg and zinc being 3.771 kg, emphasizing the role played by partitioning of trace metals into solid residues in enhancing the freshwater ecotoxicity despite improving impacts in other categories. Recovery helped very little in both scenarios, as plastic recycling could counteract only about 2% of the gross burden in SC1, and the combination of recycling and energy recovery being under 9% for SC2. So, the metal‐leaching burden remained largely uncompensated in both cases as shown in Figures 9 and 10. This trend contrasts with previous LCA studies on WtE systems, where thermochemical treatment has reduced ecotoxicity while improving other environmental indicators. For instance, the study conducted by Rahman et al. (2024) [34] on LCA of hydrothermal carbonization of MSW for WtE systems reported a 46.77% improvement in FETP compared to landfilling. Conversely, the 2.3‐fold increase observed in our study for SC2 suggests that the higher incineration throughput and the associated residue‐related emission flows in the present model can offset the ecotoxicity benefits reported for other thermochemical WtE systems. Unlike typical MSW, medical waste contains lower biodegradable content but higher concentrations of trace toxic elements, such as Zn, Cu, Hg, Cd, Pb, Cr, Ni, and As, which can effectively reduce methane production and land‐use impacts when shifting from landfill disposal to incineration. However, these metals simultaneously intensified the freshwater ecotoxicity potential by concentrating in residual ash streams instead of being diluted in waste [36]. Therefore, the elevated FETP in SC2 was primarily associated with the larger metal‐related emission flows modelled for incineration residue management under the higher waste throughput.

#### 3.4.4 Marine Ecotoxicity Potential (METP)

Marine Ecotoxicity Potential (METP) reflects the potential adverse effects of toxic substance releases on marine ecosystems and is typically expressed in kg 1,4‐dichlorobenzene equivalents (1,4‐DCB‐eq) [37]. As seen from Figure 7d, the outcomes showed that the METP rose from 1844.74 kg 1,4‐DCB‐eq in SC1 to 4258.51 kg 1,4‐DCB‐eq in SC2, thus showing a nearly 130.8% rising. In SC1 the highest emitter was incineration at 1077.90 kg 1,4‐DCB‐eq, roughly 57% of the gross inventory, and most of it came from upstream operations rather than from the plant, generated through the disposal of incineration residue and leakage of antimony, zinc, copper and chromium (VI) to ground and surface waters through landfilling, with the minor contribution of mercury coming through the flue gas emissions; chemical disinfection and autoclaving came next in line, which were emitting metals directly to ground water through open‐dumping of residual plastics (Figure 9). Similarly in SC2 the same process took place at much higher levels, with incineration accounting for 4103.20 kg 1,4‐DCB‐eq, representing around 88% of the inventory and 3.8 times the baseline, out of which landfilling of residue accounted for 3913.62 kg 1,4‐DCB‐eq, primarily due to antimony ion (2.818 kg, groundwater, CF 460 kg 1,4‐DCB‐eq/kg), zinc II (3.771 kg, CF 299) and copper ion (1.595 kg, CF 193) and doubling of stack mercury to 0.587 kg (CF 240); chemical disinfection and autoclaving contributed next, while energy recovery and recycling offset less than 9% of the load (Figure 10). Marine ecotoxicity therefore remained a residue‐leaching problem that SC2 made considerably worse. The study done by Deepak et al. (2022) [12] showed opposite findings as they obtained 13.3% reduction in marine aquatic ecotoxicity by simulating an alternative scenario of incineration with electricity recovery. The region of contrast with this study, however, lies in the fact that the study by Deepak et al. (2022) [12] considered equal incineration throughput for both of their cases while this paper considers a complete incineration of HCW for enhanced energy recovery. Moreover, the impact of bottom ash was not considered in their study and after considering this, this paper reached to a higher METP value in SC2.

#### 3.4.5 Terrestrial Ecotoxicity Potential (TETP)

Terrestrial Ecotoxicity Potential (TETP) represents the potential adverse impacts of toxic substance releases on terrestrial ecosystems and is typically expressed in kg 1,4‐dichlorobenzene equivalents (1,4‐DCB‐eq) [38]. Figure 7e shows that TETP increased from 396 125.79 kg 1,4‐DCB‐eq in SC1 to 807 173.89 kg 1,4‐DCB‐eq in SC2, corresponding to an approximately 103.8% increase. In both scenarios, the terrestrial ecotoxicity profile was strongly governed by trace toxic metals in incinerator flue gas, where small emitted masses can translate into extremely large characterized impacts due to high toxicity factors. In particular, the increase in SC2 was consistent with the higher mercury release (0.587 kg Hg(II) in SC2 versus 0.269 kg Hg(II) in SC1; CF = 1.38 × 10^6^ kg 1,4‐DCB‐eq/kg), which alone was sufficient to amplify TETP substantially. A study on achieving the sustainable medical plastic management using LCA by Cho et al. (2024) [14] also mentioned the rise of ecosystem toxicity due to incinerator generated pollutants. Further contributions arose from metal‐related emissions associated with transportation and chemical disinfection, including copper emissions from brake wear and emissions from sodium hypochlorite production. However, the dominant contribution was driven by the high sensitivity of TETP to metal emissions, particularly from incinerator flue gas, which was associated with high characterization factors (Figures 9 and 10). Terrestrial ecotoxicity was thus dominated by mercury emission in both systems, and stack‐side mercury control is the only intervention that would materially change either result.

#### 3.4.6 Fossil Fuel Potential (FFP)

Fossil Fuel Potential (FFP) represents the potential depletion of fossil energy resources and is commonly expressed in kg oil‐eq, reflecting upstream extraction of crude oil, natural gas, coal, and related fossil feedstocks rather than direct on‐site emissions [39]. In Figure 7f, SC1 showed a near‐neutral fossil resource demand, finishing at 4.085 kg oil‐eq, which arose from 664.850 kg oil‐eq of burdens almost entirely counterbalanced by −660.765 kg oil‐eq of credits (≈ 99.4% offset) (Figure 9). By contrast, SC2 turned decisively net‐negative, reaching −5239.708 kg oil‐eq, as credits of −7197.056 kg oil‐eq exceeded burdens of 1957.349 kg oil‐eq, indicating an overall fossil resource saving (Figure 10). The small net value in SC1 reflected the close balance between fossil‐intensive upstream inputs, dominated by sodium hypochlorite production (322.442 kg oil‐eq) and diesel supply for transportation (234.741 kg oil‐eq), and the substantial credit from plastic recycling (−660.765 kg oil‐eq). In SC2, the gross fossil resource demand increased relative to SC1, with major upstream burdens associated with residue handling and auxiliary material/fuel requirements (1 334.403 kg oil‐eq) alongside sodium hypochlorite production (323.397 kg oil‐eq) and transportation diesel supply (275.285 kg oil‐eq). However, incorporating WtE technology recovered a total of 20572.28 kWh of electricity produced through incineration flue gases using Rankine cycle in the alternative scenario SC2 (Section S4.2). Therefore, SC2 achieved a much larger total offset through combined recovery credits, led by energy recovery (−4,773.089 kg oil‐eq) and supplemented by enhanced plastic recycling (−2423.967 kg oil‐eq, notably polypropylene −1197.851 kg oil‐eq, HDPE −536.315 kg oil‐eq, PVC −431.187 kg oil‐eq, and PET −258.614 kg oil‐eq). Nematollahi et al. (2024) [22] also reported a significant reduction in fossil fuel depletion in their comparative LCA study in Iran, by transforming their process from 100% landfilling to 70% incineration and obtained 52.3% less fossil fuel depletion which is consistent with this paper's finding. The great fossil resource saving effect obtained in SC2 was due to the joint contribution made by energy and material recovery, whereby heat integration avoided the need of the auxiliary boiler in autoclaving process. On the other hand, systems without any technology to integrate energy consumption, as in SC1, are bound to use fossils in order to support operations, hence making them contribute toward greater fossil depletion footprint. The transition to negative FFP in SC2 from SC1 further indicates that integrating WtE recovery transforms medical waste management into a net energy‐contributing system, substantially improving resource efficiency and effectively decoupling waste management from fossil fuel dependency.

#### 3.4.7 Freshwater Eutrophication Potential (FEP)

Freshwater Eutrophication Potential (FEP) quantifies the potential enrichment of freshwater systems by nutrient releases and is commonly expressed in kg phosphorus equivalents (kg P‐eq) [40]. The results indicate that FEP increased from 2.748 kg P‐eq in SC1 to 5.419 kg P‐eq in SC2, representing an approximately 97.2% increase (Figure 7g). In SC1, the net impact comprised 2.955 kg P‐eq of burdens and a small offset of −0.207 kg P‐eq from recovery. In SC2, burdens rose to 8.772 kg P‐eq, while credits increased to 3.352 kg P‐eq (Figures 9 and 10). The higher FEP in SC2 was primarily associated with increased phosphate releases linked to residue streams, reflected by the substantially larger phosphate leaching inventory (22.895 kg phosphate in SC2 versus 5.92 kg phosphate in SC1; CF = 0.33 kg P‐eq/kg). Although recovery credits were more pronounced in SC2, particularly the energy recovery credit (−2.593 kg P‐eq) along with the recycling credit (−0.759 kg P‐eq, led by polypropylene −0.272 kg P‐eq and polyvinylchloride −0.256 kg P‐eq), these credits remained insufficient to offset the additional phosphate‐related burden from residue disposal. Çetin et al. (2025) [23] also reported a slightly higher eutrophication potential for larger proportion of incineration in their study on LCA of medical waste management. As phosphate leaching from landfilled incineration residues was the dominant contributor to FEP in SC2, further reductions in this category would depend primarily on improved residue stabilization and containment rather than on increasing the existing recovery credits. This indicates that, for FEP, the environmental benefit of the integrated SC2 system is constrained by the management of incineration residues.

#### 3.4.8 Marine Eutrophication Potential (MEP)

Marine eutrophication (MEP) is driven by emissions of nitrogen‐based compounds such as ammonium (NH_4_^+^), nitrate (NO_3_^−^), and nitrogen gas (N_2_), which leads to algal blooms in the marine environment [41]. Marine eutrophication potential (MEP) decreased from 5.311 kg N‐eq in the base case scenario (SC1) to 2.968 kg N‐eq in SC2, indicating an overall 44.1% reduction (Figure 7h). The marine eutrophication profile was primarily associated with nitrogen leaching from residual plastic that is open‐dumped, releasing bioavailable nitrogen species to groundwater and surface water [41]. In SC1, the net MEP (5.311 kg N‐eq) was largely driven by leaching of organically bound nitrogen (11.49 kg; CF = 0.297 kg N‐eq/kg) and ammonium (7.03 kg; CF = 0.23 kg N‐eq/kg). These compounds were mobilized in landfill systems during anaerobic degradation and subsequently leached into groundwater, promoting algal proliferation [41]. The elimination of landfilling in SC2 suppressed this nitrogen leaching pathway, consequently minimizing nutrient transport and enrichment into marine systems. In SC2, the lower net MEP (2.968 kg N‐eq) corresponded to reduced nitrogen loads, including organically bound nitrogen (7.066 kg; CF = 0.297 kg N‐eq/kg), ammonium (4.323 kg; CF = 0.230 kg N‐eq/kg), with smaller contributions from nitrate (0.455 kg; CF = 0.0671 kg N‐eq/kg) and nitrogen reaching surface water (0.169 kg; CF = 0.297 kg N‐eq/kg). Although SC2 achieved higher recovery credits (−0.414 kg N‐eq) than SC1 (−0.027 kg N‐eq) (Figures 9 and 10), the results suggest that minimizing marine eutrophication depends strongly on controlling nitrogen‐bearing leachate pathways from residual plastic streams. This analysis is consistent with findings from Khatun et al. (2025) [32], where improved containment configurations of nutrient emissions in the system reduced eutrophication potential by 41.91–92.22% when compared to conventional practices. Similarly, Deepak et al. (2022) [12] reported 11.38% decrease in eutrophication for incineration as a WtE system. The conversion of organic nitrogen into N_2_, NO_x_, and N_2_O during incineration in SC2 and its subsequent management with controlled emission systems substantially limit the transfer of bioavailable nitrogenous compounds into aqueous environments. The results suggest that landfill avoidance, integrated with a waste‐to‐energy system, can effectively reduce nitrogen flux from landfills in SC1 and minimize nitrogen‐driven impacts on marine ecosystems.

#### 3.4.9 Human Toxicity Potential: Carcinogenic (HTPc)

Carcinogenic Human Toxicity Potential (HTPc), expressed in kg 1,4‐dichlorobenzene (1,4‐DCB) equivalent, measures the potential carcinogenic impacts of heavy metals such as chromium (Cr VI), mercury (Hg), arsenic (As), lead (Pb), and nickel (Ni) [42]. Human Toxicity Potential, carcinogenic (HTPc) increased markedly from 4739.15 kg 1,4‐DCB‐eq in SC1 to 17 796.27 kg 1,4‐DCB‐eq in SC2 (Figure 7i), corresponding to an approximately 275.5% increase. This increase reflects a strong shift toward carcinogenic toxicity burdens associated with heavy‐metal and persistent pollutant from both incinerator bottom ash and stack emission, where relatively small mass flows can translate into large impacts due to high characterization factors. In both scenarios, carcinogenic toxicity was governed primarily by hexavalent chromium (Cr(VI)) leaching, which carried an exceptionally high characterization factor (7440 kg 1,4‐DCB‐eq/kg) and therefore dominated the category even at sub‐kilogram releases. Accordingly, Cr(VI) leaching to groundwater and surface water in SC1 (0.464 kg and 0.147 kg, respectively) produced over 4540 kg 1,4‐DCB‐eq, while the higher Cr(VI) released in SC2 (1.795 kg to groundwater and 0.568 kg to surface water) generated more than 17 500 kg 1,4‐DCB‐eq, explaining most of the increase in HTPc. Additional contributions in SC2 arose from carcinogenic metal species such as arsenic ion (0.088 kg, CF = 345 kg 1,4‐DCB‐eq/kg) and nickel(II) (0.613 kg, CF = 22.8 kg 1,4‐DCB‐eq/kg), along with smaller direct contributions from mercury (e.g., Hg(II) to air) and trace dioxins, which carried very high toxicity factors even at extremely low masses. Although recovery credits were larger in SC2 (−466.20 kg 1,4‐DCB‐eq) than in SC1 (−46.91 kg 1,4‐DCB‐eq) (Figures 9 and 10), they remained small relative to the dominant Cr(VI)‐driven burdens and therefore did not offset the higher carcinogenic toxicity potential in SC2. Comparable observations were noted in Rahman et al. (2024) [34] HTC‐LCA study, where waste‐to‐energy pathways exhibited elevated toxicity‐related impacts due to metal mobilization during thermal conversion. The results therefore indicate that, despite the recovery benefits of the integrated system, effective control and stabilization of metal‐bearing incineration residues remain essential to minimize the associated carcinogenic toxicity burden in SC2.

#### 3.4.10 Human Toxicity Potential: Non‐Carcinogenic (HTPnc)

Human Toxicity Potential, non‐carcinogenic (HTPnc) represents the potential human‐health impacts associated with exposure to substances that cause adverse health effects other than cancer, and is also expressed in kg 1,4‐DCB‐eq [43]). In this study, HTPnc increased from 38 246.93 kg 1,4‐DCB‐eq in SC1 to 65 554.65 kg 1,4‐DCB‐eq in SC2 (Figure 8a), representing an approximately 71.4% increase. Nematollahi et al. (2024) [22] reported that for hospital waste management, 70% incineration along with 20% recycling and 10% landfilling performed better than 100% landfilling in case of human non‐carcinogenic health impact, opposing to the finding of this paper. This behavior can be primarily explained by the strong influence of toxic metal emissions and leaching pathways from stack emission and bottom ash on non‐carcinogenic toxicity characterization, where relatively small releases of specific metals yield large contributions due to high characterization factors. In SC1, HTPnc was driven by a combination of (i) metal emissions to air, dominated by mercury (6661.28 kg 1,4‐DCB‐eq) and lead (4074.21 kg 1,4‐DCB‐eq), and (ii) metal leaching, particularly Zn(II) (0.975 kg, CF = 8240 kg 1,4‐DCB‐eq/kg) together with leaching‐related contributions associated with arsenic and antimony, alongside substantial toxicity burdens linked to leaching from residual plastic disposal (e.g., Zn, Pb, As). In SC2, the increase was consistent with higher metal loads in both major non‐carcinogenic pathways: the inventory indicated a larger mercury release (0.587 kg Hg(II), CF = 2.48×10^4^ kg 1,4‐DCB‐eq/kg) and additional lead contributions (CF = 2.23×10^4^ kg 1,4‐DCB‐eq/kg), while residue‐associated leaching increased sharply, notably zinc(II) (3.771 kg, CF = 8,240 kg 1,4‐DCB‐eq/kg) and arsenic ion to surface water (0.088 kg, CF = 8.74×10^4^ kg 1,4‐DCB‐eq/kg), with further contributions from antimony ion (CF = 805 kg 1,4‐DCB‐eq/kg) and mercury(II) in aqueous pathways (CF = 5.62×10^4^ kg 1,4‐DCB‐eq/kg). Although SC2 achieved larger recovery credits (−6968.63 kg 1,4‐DCB‐eq) than SC1 (−663.25 kg 1,4‐DCB‐eq), these offsets removed less than 10% of the total load and therefore did not compensate for the higher toxicity burden arising from metal‐related emissions and leaching (Figure 10). Overall, the higher HTPnc in SC2 highlights the importance of controlling both atmospheric metal emissions and releases from incineration residues. Although SC2 provides greater recovery benefits, these benefits are accompanied by a substantially higher toxicity burden under the current residue management assumptions.

#### 3.4.11 Land Occupation Potential (LOP)

Land occupation is measured in square meters years of agricultural crop equivalent (m^2^×a crop‐Eq) and represents the area occupied for waste disposal over a specific time [44]. Agricultural land occupation potential (LOP) decreased markedly from 965.91 m^2^·a crop‐eq in SC1 to 28.84 m^2^·a crop‐eq in SC2, as evidenced in Figure 8c. This represented an approximately 97.0% reduction, primarily associated with the substantial reduction in land‐demanding disposal requirements when shifting away from the landfill‐dependent baseline configuration. In SC1, the high LOP was dominated by long‐term land commitment for disposal, resulting in 977.19 m^2^·a crop‐eq of burdens with only a minor offset (−11.28 m^2^·a crop‐eq) (Figure 9). By contrast, SC2 substantially lowered the net land occupation to 28.84 m^2^·a crop‐eq, despite 124.02 m^2^·a crop‐eq of burdens, because recovery credits were much larger (−95.18 m^2^·a crop‐eq, offsetting ∼77% of the total land demand) (Figure 10). The substantial reduction in LOP shows that SC2 can significantly reduce the long‐term land burden by diverting waste from landfilling and increasing material and energy recovery. This makes reduced land occupation a clear environmental advantage of the integrated SC2 system.

#### 3.4.12 Particulate Matter Formation Potential (PMFP)

Particulate matter (PM) formation, expressed in kg PM_2.5_ eq, measures the release of fine particles into the air, which can potentially form airborne particles smaller than 2.5 micrometers [45]. Particulate matter formation potential (PMFP) showed a pronounced improvement, decreasing from 20.364 kg PM_2.5_‐eq in SC1 to a marginally negative −0.135 kg PM_2.5_‐eq in SC2 (Figure 8f). The large reduction reflects the system transitioning away from landfill‐driven precursor formation pathways, particularly NH_3_, which promotes secondary particulate formation through reactions with acidic species, and toward a configuration where particulate‐related burdens are largely counterbalanced by recovery benefits. In SC2, PMFP became a near break‐even result because burdens (19.894 kg PM_2.5_‐eq) were totally counterbalanced by credits (−20.029 kg PM_2.5_‐eq), yielding the small net negative value. The burdens in SC2 were strongly associated with combustion‐related precursors, especially NO_x_ (68.421 kg; CF = 0.11 kg PM_2.5_‐eq/kg), alongside direct PM_2.5_ (3.115 kg; CF = 1.0 kg PM_2.5_‐eq/kg) and SO_2_ (7.977 kg; CF = 0.29 kg PM_2.5_‐eq/kg). However, the transition to incineration introduced NO_x_ formation, making it a dominant source of particulate matter through secondary reactions. Furthermore, the combination of pollution control technology, enhanced plastic recycling rates, and energy recovery in SC2 have reduced upstream emissions from material extraction and energy production, thereby decreasing overall PM formation.

#### 3.4.13 Photochemical Oxidant Formation Potential: Terrestrial Ecosystem (EOFP)

Photochemical oxidant formation measures the potential of emitted gases (NO_x_ and VOCs) to react in the atmosphere and form harmful smog and ozone in kg NO_x_‐Eq [46]. Photochemical oxidant formation potential (EOFP) increased from 15.64 kg NO_x_‐eq in SC1 to 47.30 kg NO_x_‐eq in SC2 (Figure 8h), primarily driven by the ∼8‐fold intensification in the incineration process from 5.54 tons/day in SC1 to 42.13 tons/day in SC2. Consequently, the contribution of incineration increases from 10.06 kg NO_x_‐Eq to 77.22 kg NO_x_‐Eq, indicating that large‐scale incineration in the WtE model without proper emission control technology can significantly increase the oxidant formation potential. In both scenarios the category was governed by a single elementary flow, direct stack emissions of nitrogen oxides, which rose from 8.98 kg in SC1 to 68.42 kg in SC2. With a characterization factor of 1.00 kg NO_x_‐Eq/kg, this emission alone accounts for 8.98 of the 10.06 kg NO_x_‐Eq attributed to incineration in SC1 and 68.42 of the 77.22 kg NO_x_‐Eq in SC2. Transportation and chemical disinfection together contributed about 8 kg NO_x_‐Eq in SC1 and 7 kg NO_x_‐Eq in SC2, remaining minor in both cases. Plastic recycling offset only 14% of the gross burden in SC1, whereas plastic recycling and energy recovery together cleared roughly 44% in SC2, though still not enough to counterbalance the stack emissions (Figures 9 and 10). The trend was not consistent with results from Deepak et al. (2022) [12], where integrating incineration as a WtE system demonstrated lower photochemical oxidation potential by 12.81%. It is because, Deepak et al. (2022) [12] incinerated same amount of waste in both scenarios while generating electricity in SC2 which offset EOFP to some level. A study by Khatun et al. (2025) [32] reported 49.77–57.19% increase in photochemical oxidant formation in controlled systems, despite overall environmental improvements. However, these SC2 emissions originate from point sources, enabling the scope to implement advanced mitigation strategies. The integrated implementation of advanced flue gas treatment systems, such as Selective Catalytic Reduction (SCR) or Selective Non‐Catalytic Reduction (SNCR), with optimized combustion conditions and low‐NO_x_ burners can efficiently mitigate NO_x_ emissions from incineration in the alternative scenario.

From the above discussion, it is evident that the alternative scenario (SC2) offered clear environmental benefits in impact categories associated with landfill diversion and resource and energy recovery. On the other hand, the baseline system (SC1) demonstrated lower effects on various indicators of toxicity and photochemical oxidant formation.

The advantage of SC1 regarding the results of toxicity, however, must be treated with some cautions due to the fact that it is largely influenced by a discrepancy in the modelling of the incineration residue leaching. The ecoinvent process that is used to model residue treatment, considered cement‐stabilization of bottom ash before being landfilled. Such a presumption is relevant for SC2 as incineration residue stabilization is considered but not applicable for SC1 when the residue is normally disposed of directly. Thus, the usage of stabilized‐residue data set in both cases led to the decreased burden of SC1 associated with leaching. In case SC1 was evaluated using the dataset of the untreated incineration residue, the toxicity impacts were likely to increase and the difference between the scenarios would have been reduced. Nevertheless, the overall conclusion holds true: toxicity effects are very sensitive to the way incineration residue is being treated and disposed of.

### 3.5 Hotspot Analysis: Dominant Process Contributors and Scope for Improvement

Hotspot analysis is a systematic method for identifying the specific processes, emissions, material flows, or life‐cycle stages that contribute most significantly to the overall environmental impacts of a system within the life‐cycle assessment (LCA) [47]. It disaggregates results to show where the dominant burdens originate, allowing researchers to identify the components primarily responsible for various environmental impacts [48]. Hotspot analysis in healthcare waste management is particularly important, as it involves multiple interconnected processes, including segregation, transport, disinfection, autoclaving, incineration, recycling, and landfill disposal. Therefore, it is very crucial in our study, as it can explain why one scenario outperforms the other by identifying the unit operations. This provides practical analyses of areas for improvement in SC2, and ultimately helping decision‐makers strengthen policy relevance. The hotspot analysis was performed across all eighteen impact categories using the process‐level contribution trees generated in openLCA. The processes examined were transportation, landfilling, autoclaving, incineration, chemical disinfection and shredding on the burden side, together with plastic recycling in both scenarios and energy recovery in SC2 on the credit side. Results were expressed as percentage contributions to the gross burden, so that the two scenarios could be compared on process structure rather than on absolute magnitude alone (Figures 9 and 10; Tables S17 and S18).

In SC1 (Figure 9), the burden was distributed across six processes. Landfilling dominated acidification (60%), climate change (89%) and land use (92%), and all of this was released directly at the site through ammonia and methane generation and the physical occupation of the dump area. Incineration governed the toxicity categories, accounting for 95% of terrestrial ecotoxicity and 97% of carcinogenic human toxicity, together with 55–58% of freshwater and marine ecotoxicity and non‐carcinogenic human toxicity, and it further led particulate formation (68%) and photochemical oxidant formation (56%). Chemical disinfection controlled the categories driven by background supply chains rather than by waste treatment itself, contributing 92% of ionising radiation, 89% of mineral resource depletion and 94% of water consumption, almost entirely through sodium hypochlorite manufacture. Autoclaving and chemical disinfection jointly governed marine eutrophication (98%), not through their treatment operations but through the open dumping of the residual plastic they generated, while transportation was significant only for photochemical oxidant formation (24%) and fossil resource depletion (35%). Shredding was negligible throughout, contributing under 1% of the gross burden in every category, as its only input was a small quantity of grid electricity. Recovery in SC1 was limited to plastic recycling, which offset 99.4% of fossil resource depletion and 80.4% of ozone depletion but less than 10% of the burden in eleven of the eighteen categories.

In the proposed scenario (Figure 10), the burden became concentrated rather than distributed. With landfilling eliminated, incineration emerged as the single dominant process in thirteen of the eighteen categories, contributing over 99% of carcinogenic human toxicity, 97.5% of terrestrial ecotoxicity, 92–93% of climate change, freshwater eutrophication and photochemical oxidant formation, and 85–88% of acidification, ecotoxicity and non‐carcinogenic human toxicity. Its contribution was of two distinct types: direct stack releases of NO_x_, SO_2_, fossil CO_2_ and mercury, which drove acidification, climate change, oxidant formation and terrestrial ecotoxicity; and upstream burdens from landfilling the incineration residue, which drove the ecotoxicity, eutrophication and human toxicity categories through heavy‐metal and phosphate leaching. Chemical disinfection retained its role in the same supply‐chain‐driven categories as in SC1, contributing 76% of ionising radiation, 76% of mineral resource depletion and 79% of water consumption, while transportation became prominent only in land use (23%) through allocated road construction, and shredding again remained negligible. The credit structure also changed fundamentally, with energy recovery and plastic recycling together offsetting 97% of acidification, 101% of particulate formation, 167% of water consumption, 368% of fossil resource depletion and 441% of ozone depletion, reversing the sign of four categories. Critically, however, these credits cleared only 3–10% of the toxicity and residue‐related burdens (HTPc, TETP, FETP, METP and HTPnc), and about 38% of FEP, which are precisely the categories in which SC2 performed worst.

The hotspot analysis revealed that SC2 merely moved the environmental burden rather than removing it by switching from the diffuse landfill‐based burden to the concentrated incineration‐based one. Although the switch is a favorable move in theory as point source pollution can be engineered against but not landfill‐based pollution, the advantage is achieved only when the corresponding controls are put in place [49]. The three actions are needed: first, treatment of flue gas: injection of activated carbon with mercury‐specific sorbents can take care of the mercury alone responsible for TETP, whereas SCR or SNCR and acid gas scrubbing can reduce the NO_x_ and SO_2_ responsible for TAP, EOFP and PMFP. Second, residue control: stabilization or vitrification of bottom ash and fly ash before landfilling can reduce the leaching of chromium(VI), antimony and zinc responsible for HTPc, FETP, METP and HTPnc, and phosphate responsible for FEP. Third, residual plastics control: preventing the plastic fraction from being dumped in an open dump can deal with MEP, the single burden category where incineration is not responsible. This combination would take care of the problems left unresolved by the recovery credits, and help SC2 maintain its advantages related to the environment and land use, without shifting the burden to the toxic effects.

### 3.6 Normalized Impact Assessment

The normalization of the characterized results into ReCiPe 2016 World (2010) H reference set ensures that all the categories are expressed in person‐equivalents (Pe), making them comparable [50] (Figure 11; Table S19). In this way the assessment is viewed very differently compared to the results of the characterization. The toxicity categories dominated the entire result, contributing approximately 97% of the normalized burden in SC1 and 99% in SC2, with carcinogenic human toxicity being the largest component in each scenario at 460 Pe and 1,728 Pe correspondingly. Climate change is an example of the impact that was seen the most different in terms of normalized results. While being the dominating one in characterized assessment and improving 78% in SC2, the climate change burden constituted only 1.6% in SC1 and 0.1% in SC2. The overall positive burden thus increased from approximately 600 Pe in SC1 to 2,017 Pe in SC2, with the major contribution coming from the 3.8‐fold increase in carcinogenic human toxicity, similar to Section 3.4, where the leaching of hexavalent chromium from incineration ash landfilling was established as a dominant pathway.

**FIGURE 11:**
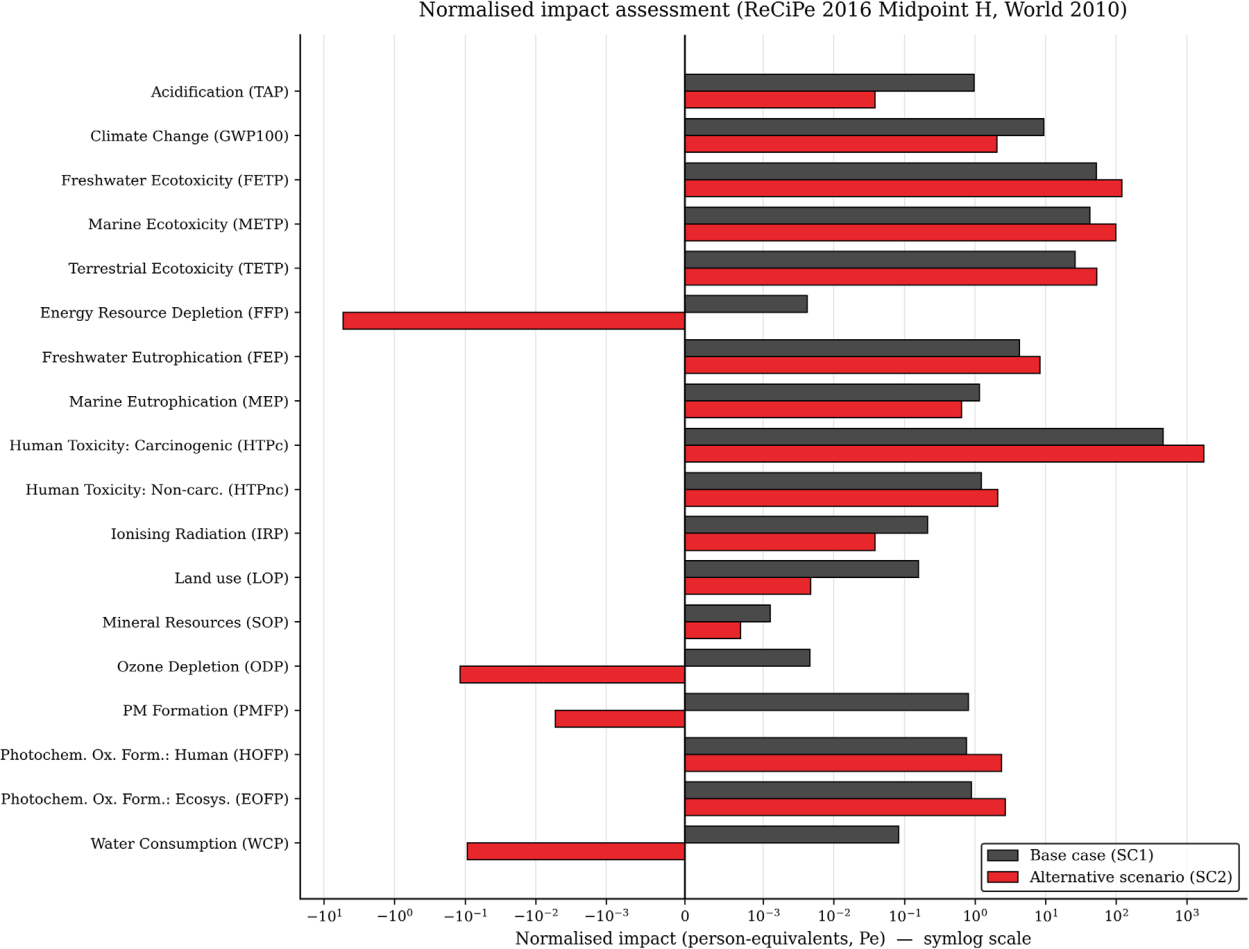
Normalized life cycle impact assessment results for the base case (SC1) and the proposed alternative scenario (SC2). A symmetric logarithmic scale is used on the horizontal axis to accommodate values spanning more than six orders of magnitude, including negative results arising from avoided burdens in the alternative scenario.

Some cautions are required to interpret the above discussion. The toxicity characterization factors in the ReCiPe 2016 methodology have more uncertainty due to modelling than standard midpoint indicators; hence the size of the difference between the scenarios is approximate rather than precise [50, 51]. Also, the World (2010) dataset is representative of the global average conditions of 2010, not of the contemporary situation in the study area [50]. This applies to the accuracy, not the direction, of the estimate; the toxicity burden of SC2 was definitely much higher than that of SC1 by an amount that could not possibly be explained away by uncertainty in the factors, and the source was clearly defined – hexavalent chromium leaching from the landfill containing incineration ash, and mercury emissions with the flue gas. The normalized figures therefore confirm the outcome of the hotspot analysis – residue management and stack emissions control are the key factors in this system. Controlling ash leaching and mercury emissions at the stack would have a bigger impact on the overall footprint than energy production and transportation measures alone.

### 3.7 Sensitivity Analysis

Five parameters identified as the main drivers in the hotspot analysis were tested individually: the gross electricity recovery rate (P1), the quantity of incineration residues (P2), the NaOCl dose in chemical disinfection (P3), the quantity of plastics recycled (P4), and the transport distance (P5). Each was varied by ±20% in openLCA, except plastic recycling, which was varied only downward by 20% and 40% because SC2 already assumed the maximum realistic recycling potential. Results for all eighteen categories are shown in Figures 12 and 13, and the corresponding values are given in Tables S20–S24.

**FIGURE 12:**
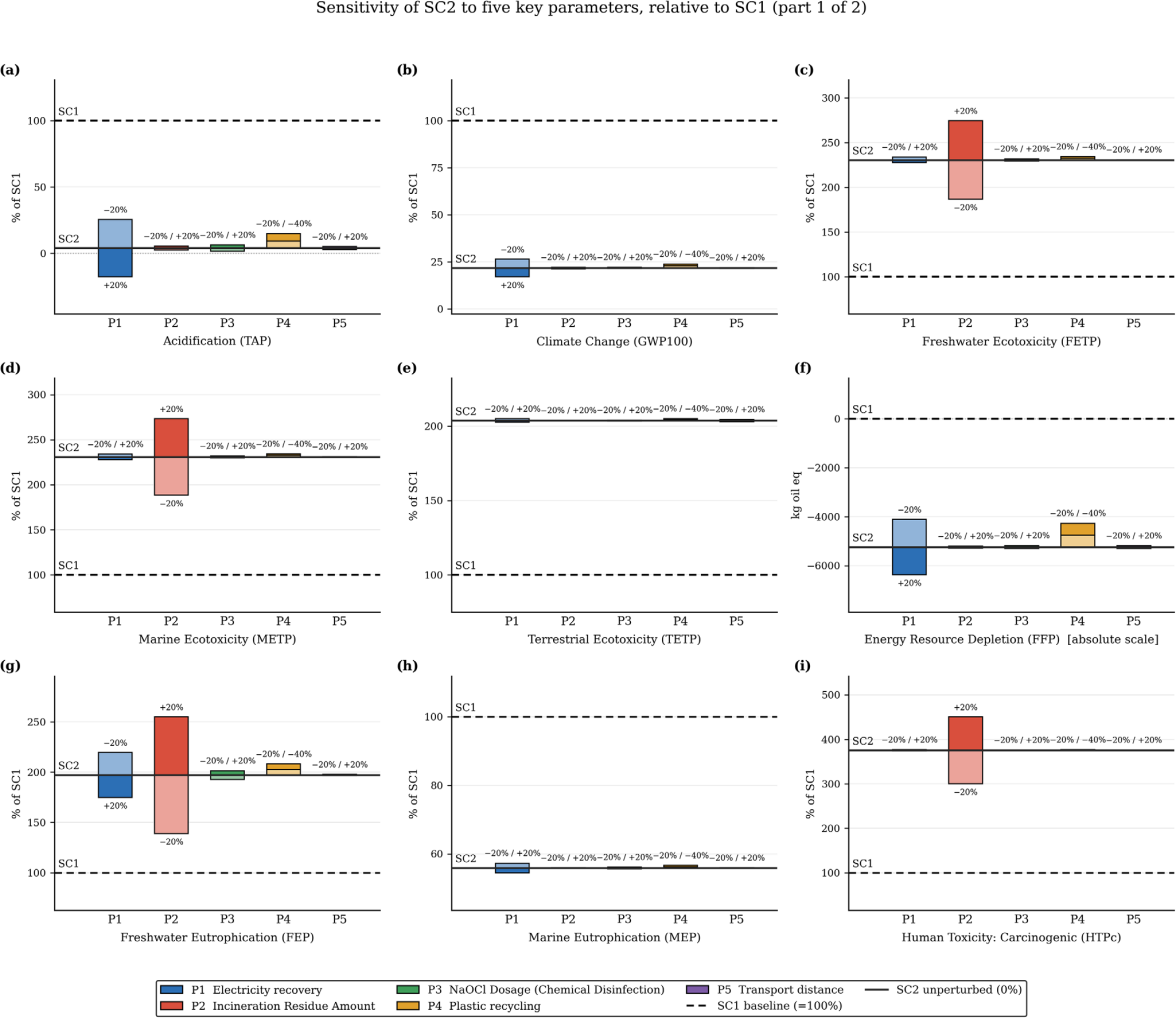
Sensitivity analysis for SC2 to five key parameters with respect to SC1 (Part 1 of 2).

**FIGURE 13:**
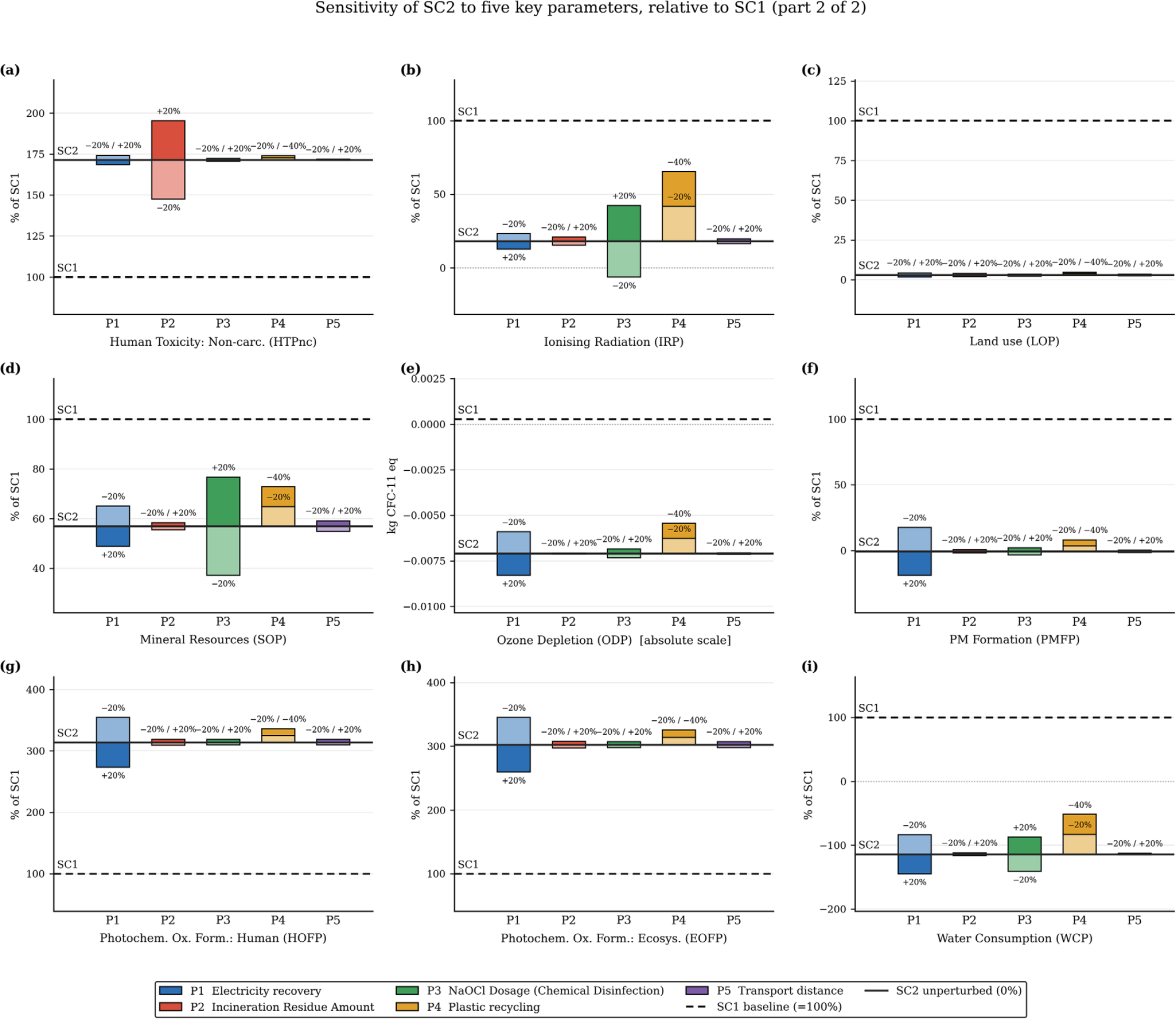
Sensitivity analysis for SC2 to five key parameters with respect to SC1 (Part 2 of 2).

The residue quantity (P2) exhibited the largest range for the toxicity and eutrophication categories. For HTPc it varied from 300% to 451% compared to the baseline 375%, for FEP from 139% to 255% compared to 197%, for FETP from 187% to 274%, for METP from 188% to 273%, and for HTPnc from 148% to 195%. TETP and MEP were insensitive to the changes, since neither of them depended on the residue leaching. The electricity recovery rate (P1) had the largest impact on the credit‐based categories. EOFP varied from 260% to 345%, HOFP from 273% to 355%, while WCP varied from −84% to −145%, TAP from −18% to 26%, and PMFP from −19% to 18%. The GWP100 and toxicity categories showed stability for this parameter, varying by no more than 10 per cent. The impact of P3 and P4 on the performance of the respective categories was more constrained, but very intense among them. Changing the NaOCl dosage had an effect on IRP ranging between −6% and 42% of SC1, and SOP ranging between 37% and 77%, and made WCP shift between −141% and −87%. A decrease in recycling by 40% increased IRP to 65% and SOP to 73%, and improved WCP by shifting it from −114% to −52%. Increasing transport distance (P5) had an effect of less than 5 percentage points on every category. Across all perturbations, SC2 never crossed the SC1 line in any category. Categories in which SC2 performed better remained below 100% even under the least favourable parameter value, and those in which it performed worse remained above it. The comparison between the two scenarios is therefore robust to the ranges tested.

## 4 Sustainability Assessment and SDG Mapping

The comparative LCA analysis demonstrated that transitioning from SC1 to the proposed integrated model SC2 based on improved segregation, advanced plastic recycling, and WtE integration allows achieving multidimensional sustainability gains that go beyond merely environmental indicators. Environmentally, SC2 lowers several systemic burdens, especially in terms of terrestrial acidification (96.06%), climate change (78.28%), land occupation (97.01%), ionizing radiation (81.96%), marine eutrophication (44.12%), and surplus ore potential (43.08%). Moreover, SC2 transforms several categories into avoided burdens by combining energy and material recovery (e.g., fossil fuel potential, ozone depletion, particulate matter formation, and water consumption). In total, these changes allow achieving a transformation from the long‐term reliance on land‐based solutions to a circularity and recovery approach, which is especially relevant for a rapidly urbanizing and land‐limited city like Dhaka. Nevertheless, sustainability should be viewed as a trade‐off space, and not just a unidirectional improvement, as SC2 increases toxicity and photochemical smog‐related categories (freshwater/marine/terrestrial ecotoxicity, carcinogenic and non‐carcinogenic human toxicity, and photochemical oxidant formation), which means that the sustainability gains of SC2 depend on the implementation of fly ash/bottom ash management and NO_x_ mitigation strategies. However, the recovery of 20572.28kWh/day of electricity from the process is highly relevant in the context of Bangladesh. Recent national energy statistics indicate that gas, coal, furnace oil, and energy imports dominate the power mix, whereas the usage of renewable energy remains extremely limited [11]. Therefore, an integrated WtE recovery system from medical waste can support a diversified energy system while reducing environmental impacts and fossil fuel dependency. In the meantime, the outcomes of this study clearly demonstrate that sustainable healthcare waste management can directly support several United Nations Sustainable Development Goals (SDGs). The SDGs are a series of universal frameworks consisting of 17 interconnected global goals adopted by the United Nations (UN) to promote balanced progress across environmental protection, economic development, and social well‐being [52]. The SDGs aim to address pressing global issues, such as poverty, climate change, resource depletion, health risks, pollution, and unsustainable urbanization, through integrated, measurable targets. Each goal of the SDGs is further supported by specific targets to guide responsible authorities for implementing policies and practices. In the context of waste management research, the SDGs provide essential frameworks for linking technical improvements with environmental impacts, including public health, climate action, clean energy, and water protection.

SC2 contributes toward achieving SDG 3 (Good Health and Well‐being) through improved control and treatment as well as reduction in pathways related to air pollution and exposure to adverse environments. The significant decrease in the burden of terrestrial acidification and change in the nature of the burden for particulate matter from positive in SC1 (20.36 kg PM_2.5_‐eq) to negative in SC2 (−0.14 kg PM_2.5_‐eq) are clearly linked to Target 3.9 (deaths and illness caused by hazardous chemicals and air pollution). However, SDG 3 cannot be automatically achieved because of the increased burden from both carcinogenic (+275.52%) and non‐carcinogenic (+71.40%) human toxicity indicators. Thus, the achievement of SDG 3 can be described as conditional: although SC2 addresses a number of air‐ and exposure‐relevant burdens, additional management of residues generated through incineration is needed to address health risks from heavy metal‐related pathways. Moreover, the significant decrease in the marine eutrophication of 44.12% contributes to SDG 6 (Clean Water and Sanitation), especially Target 6.3, through the decreased nutrients‐driven water pollution [53]. In terms of water consumption potential, its value goes down from 21.91 m^3^ in SC1 to −25.00 m^3^ in SC2, thus indicating that the recovered credits outweigh water needs within the established system boundaries. Nevertheless, the increased potential of freshwater ecotoxicity by 130.50% suggests that improvements should be made regarding residue and leachate handling. The decreased marine eutrophication potential by 44.12% also leads to SDG 14 (Life Below Water), Target 14.1, through the decreased marine pollution caused by nutrients; however, the increased potential of marine ecotoxicity by 130.85% implies this contribution to be conditional. Similarly, SC2 highly contributes to SDG 7 (Affordable and Clean Energy) via energy recovery (20572.28 kWh/day) and fossil‐resource substitution. The potential fossil fuel value drops from 4.09 kg oil‐eq in SC1 to −5239.71 kg oil‐eq in SC2 due to significant fossil resource savings linked to energy recovery and material recycling. Such a contribution should be understood as fossil‐resource substitution and energy efficiency improvement, not renewable energy production. As regards SDG 11 (Sustainable Cities and Communities), the reduction of land occupation by 97.01% and reductions in particulate burden help meet Target 11.6 regarding reduction of environmental impacts of cities. Nonetheless, the rises in photochemical oxidant formation burdens for human health (+213.91%) and ecosystems (+202.46%) suggest that such sustainability contributions of cities are conditioned by proper flue gas cleaning, especially NO_x_ removal. The most compelling climate change advantage can be observed in SDG 13 (Climate Action), where SC2 reduces global warming potential by 78.28% compared to SC1 due to reduced landfill methane emissions and fossil fuel use resulting from energy recovery. Generally speaking, SC2 is a high potential transition strategy that contributes to climate change mitigation, fossil resource conservation, efficient land use, and nutrient pollution prevention, in support of SDGs 3, 6, 7, 11, 13, and 14 which is shown in Figure 14. Yet, the outcomes of the life cycle assessment show that such advantages cannot be taken for granted, since the increase in ecotoxicity and photochemical oxidant formation imply the necessity of more effective residue, containment, leachate, and flue gas control measures. In other words, the contribution of SC2 to the SDGs can be viewed as a promising but conditional sustainability improvement, in which environmental controls play an integral role in preventing possible adverse consequences. The SDGs associated with this study, along with their specific targets, are listed in the supporting information (Table S25).

**FIGURE 14:**
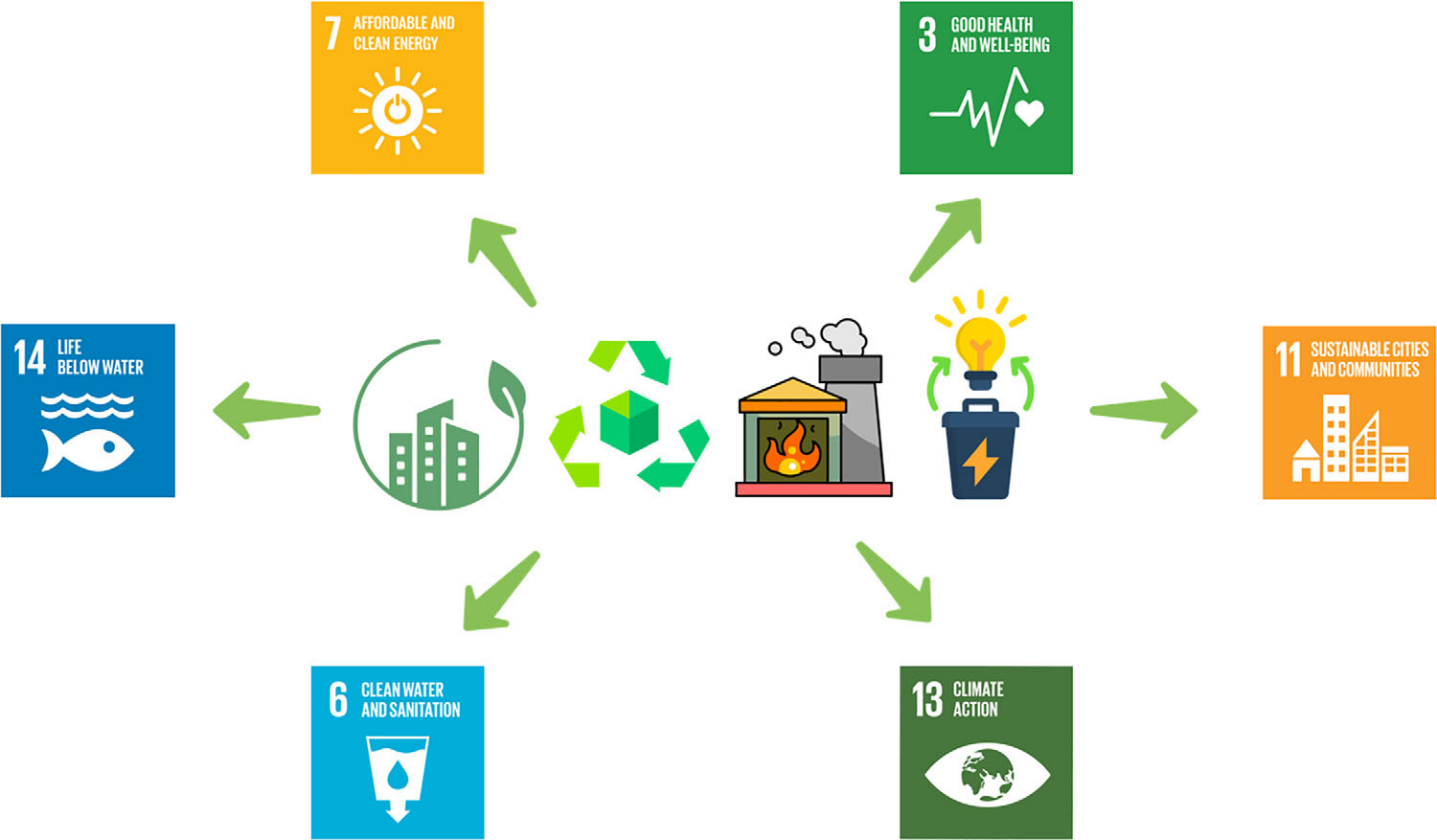
The Sustainable Development Goals (SDGs) associated with the proposed SC2 scenario.

## 5 Concluding Remarks, Study Limitations, and Way Forward

The study establishes an integrated technical framework for LCA and MFA to optimize treatment strategies in medical waste management. The results from the proposed alternative scenario (SC2) demonstrated that waste‐to‐energy recovery, plastic recycling, and improved segregation methods can significantly outperform the baseline scenario (SC1) across multiple environmental impacts. Reductions of 78.28% in climate change potential, 96.06% in acidification potential, 97.01% in land occupation, and approximately 44% in marine eutrophication were achieved through eliminating landfill disposal and implementing improved material and energy recovery strategies. Even so, a 130.50% increase in freshwater ecotoxicity potential, a 275.52% increase in carcinogenic human toxicity potential, a 71.40% increase in non‐carcinogenic human toxicity potential, and a 202.46% increase in photochemical oxidant formation potential due to incineration in SC2 highlight the importance of advanced flue gas treatment, optimized incineration conditions, and residue management systems. Hotspot analysis identified incineration as the dominant contributor in thirteen of eighteen categories in SC2, replacing the landfill‐driven profile of SC1, while normalization showed that toxicity accounts for about 97% and 99% of the total burden respectively. The study also supports the UN SDGs 3, 6, 7, 11, 13, and 14 by improving the safety of healthcare waste, recovering energy, reducing water pollution, lowering reliance on urban landfills, mitigating greenhouse gas emissions, and protecting aquatic ecosystems, although the contributions to SDGs 3, 6, 11, and 14 remain conditional on residue and flue gas control.

However, the reduction percentage of environmental impacts and the possible energy recovery and recycling technologies imply that the suggested strategic framework could become a useful guide in defining policy directions in future research. Nevertheless, despite its strengths, the current study has many limitations. The composition of the medical waste has been obtained partly by field sampling, while the rest of the data has been based on the literature values, which do not necessarily show the complexity of the situation, as the characteristics of waste stream may vary depending on the location in Dhaka. Thus, future studies should include field waste characterization methods, considering different hospitals, scales, and seasons. Furthermore, the material flow rate has been considered constant during the whole day, while the generation of the medical waste is variable depending on the patient number and outbreak of disease. In addition, emission factor data for air emissions from incineration and the boiler were derived from literature sources (Tables S26–S28). Moreover, a further limitation concerns the incineration residues, as the ecoinvent process assumes cement stabilization of bottom ash before landfilling, which suits SC2 but not SC1, where residues are disposed of directly, so the toxicity gap reported here is an upper bound. Reliance on Ecoinvent datasets for modelling incineration residue treatment means that the results represent a generalized theoretical scenario for Dhaka rather than actual site‐specific conditions. Process optimization and pilot scale validation should be integrated to identify the optimal operational conditions. Region‐specific life‐cycle inventories, combined with direct stack monitoring and field measurement systems, can improve the authenticity of future assessments. Finally, the study focused on environmental indicators, whereas occupational safety, economic feasibility, and social acceptance were not considered, leaving scope for future investigations into techno‐economic analysis, quantitative risk assessment, and social life‐cycle assessment.

This research further highlights the requirement for a collaboration between various stakeholders, which includes health care establishments, waste processing facilities, and regulatory bodies, towards sustainable medical waste management under the circular economy approach. Health care establishments must focus on source segregation of waste as per national regulations, as this is one area that helps in improving the treatment process and also decreases the environmental footprint. It is necessary to transport the waste legally and promptly to the proper disposal site in order to avoid infections. Waste processing facilities must implement a system for emission monitoring, shift towards renewable sources of energy and fuels, while treated wastewater from ETPs can be used for reprocessing water to minimize freshwater consumption. As the contribution of recovery credits is negligible in the burden of toxicity, the use of mercury sorbent, NO_x_ reduction, and ash stabilization is crucial in this regard. Energy recovery (ER) from incineration and other advanced technologies, including plasma pyrolysis and microwave sterilization, must be advocated by the government agencies in light of increased waste volumes owing to the pandemic situations. Policies must be formulated with emphasis on recycling, energy recovery, and private sector involvement. Moreover, green purchasing policies must be encouraged for medical products, while the institutions' strategies for managing their waste must be aligned with the UN Sustainable Development Goals to ensure not only environmental sustainability but also overall sustainability. In terms of future research direction, it is recommended that long‐term field surveys should be conducted to gather field‐level information about the nature of the medical waste. Also, the viability of recycling plastics can be assessed through a structured roadmap regarding collection, segregation, and processing.

## Author Contributions

**Mahmudul Hasan Sajid**: writing – original draft, methodology, investigation. **S. M. Akimus Salehin**: Writing – original draft, data curation, formal analysis. **Musfekur Rahman Dihan**: writing – review & editing, visualization, software. **Rayhan Bin Masud**: writing – original draft, data curation. **Rumana Afrin**: writing – original draft, data curation. **Md. Shahinoor Islam**: writing – review & editing, supervision, formal analysis, conceptualization. **Md. Mujibur Rahman**: writing – review & editing, supervision.

## Funding

This research was funded by the Institute for Advanced Research (IAR) of United International University (UIU) under the Grant Reference: UIU‐IAR‐02‐2024‐SE‐40.

## Ethics Statement

A part of the manuscript has been grammatically corrected using Grammarly, Quillbot, Grammar check, etc.

## Conflicts of Interest

The authors declare no conflicts of interest.

## Supporting information




**Supporting File**: gch270172‐sup‐0001‐SuppMat.docx.

## Data Availability

The data that support the findings of this study are available from the corresponding author upon reasonable request.
