# Supplementary material for: Sustainability and Integrated Energy Recovery Scheme for Health Care Waste Management for Megacity Dhaka: Through Material Flow and Life Cycle Assessment
Source: Glob Chall. 2026 Oct 31;10(11):e70172. doi: 10.1002/gch2.70172 (PMC13723702; doi:10.1002/gch2.70172)
Supplement: Supplementary file 1 [file GCH2-10-e70172-s001.docx]

**Sustainability and Integrated Energy Recovery Scheme for Health Care Waste Management for Megacity Dhaka: Through Material Flow and Life Cycle Assessment**

Mahmudul Hasan Sajid^1,2,¥^, S. M. Akimus Salehin^1,3,¥^, Musfekur Rahman Dihan^1^, Rayhan Bin Masud^1^, Rumana Afrin^4^, Md. Shahinoor Islam^1,#^ and Md. Mujibur Rahman^4,*^

*^1^Department of Chemical Engineering, Bangladesh University of Engineering and Technology, Dhaka 1000, Bangladesh*

*^2^Department of Dyes and Chemical Engineering, Bangladesh University of Textiles, Dhaka, Bangladesh*

*^3^Department of Chemical Engineering, Dhaka University of Engineering and Technology, Gazipur, Bangladesh.*

*^4^Department of Civil Engineering, United International University (UIU), Madani Avenue, Badda, Dhaka, 1212, Bangladesh*

¥: Equal Contributions

Corresponding authors:

#Md. Shahinoor Islam (shahinoorislam@che.buet.ac.bd)

*Md. Mujibur Rahman (mujibur@ce.uiu.ac.bd)

**List of Content**

**S1.** **Health Care Waste Treatment Scenario in PRISM and Literature Review**

Figure S1: PRISM approach for medical waste treatment…………………………………………….01

Table S1: Summary of previous studies on healthcare waste life cycle assessment (LCA) and material flow analysis (MFA), with identified research gaps addressed by the present study………………….01

**S2. Life Cycle Inventory Analysis**

Table S2: Background life cycle inventory datasets used for both scenarios, taken from the ecoinvent 3.9.1 database as implemented in openLCA 2.6.2……………………………………………………05

Table S3*:* Inventory analysis for the incinerator…………………………………………….………..07

Table S3A: SEM–EDX elemental composition of medical-waste incinerator bottom ash at selected spots and over an analyzed area………………………………………………………………………08

Figure S2: SEM–EDX characterization of the medical-waste incinerator bottom ash………...……..09

Table S4: Inventory analysis data for transportation………………………………………………….09

Table S5: Inventory data for the boiler and autoclave……………………………….………………..10

Table S6: Emissions from landfill and open dumping of plastic waste ……………..………………..10

Table S7: Electricity consumption of different equipment for the base and alternative scenarios….....11

**S3. Field Survey and literature data**

Table S8: Survey Data and Questionnaire……………………………………………………………..12

Table S9: PRISM-provided plastic waste sample (Source: Field visit) ………………………………..13

Figure S3: Different noninfectious plastic compositions found in medical waste …………………...13

Table S10: Maximum recyclable quantity for each type of plastic. Reprocessing potential is obtained from the literature …………………………………………………………………….………………14

**S4. Detailed Calculation**

**S4.1 Calculation of MWGR (kg/day/bed) from Empirical Correlation**

Table S11: Human Development Index (HDI), life expectancy at birth (LE), mean years of schooling (MYS), and CO2 emissions per capita (CE) for Bangladesh from 2010 to 2019 (UNDP, 2022, 2020). MWGR calculated by the three models is also presented. Values from 2020 to 2025 are extrapolated……………………………………………………………………………………………14

Table S12: Total Bed No. Count Under DNCC and DSCC (DGHS Facility Registry, 2025)………………………………………………………………………………………….……….15

**S4.2 Estimation of Power Generation in the Alternative Scenario (SC2) by Energy Balance**

Table S13: Parameters used in the estimation of power generation in the alternative scenario………16

**S4.3 Estimation of Methane Emissions from Landfill**

Table S14: Definition of different terms used in the equation………………………………………..17

Table S15: Estimation of methane emission by FODM for PRISM Landfill………………….……..18

Figure S4: Graph of methane emission (ton/day) using the IPCC FODM method…………………...19

**S5. Comparative Life-Cycle Impact Assessment Results**

**S5.1 Impact Assessment results**

Table S16: Comparative life cycle impact assessment results for the base and alternative scenarios, per functional unit of 46.32 tonnes of waste managed per day. ……………………….…………………..19

**S5.2	Hotspot Analysis**

Table S17: Process contributions (direct + upstream) to impact categories, base scenario (SC1)…....20

Table S18: Process contributions (direct + upstream) to impact categories, alternative scenario (SC2)…………………………………………………………………………………………………..21

**S5.3 Normalized Results**

Table S19: Normalised life cycle impact assessment results, expressed in person equivalents using ReCiPe 2016 global normalisation factors…………………………………………………………….23

**S5.4 Sensitivity Analysis**

Table S20: Sensitivity of SC2 to gross electricity recovery from incineration……………………….24

Table S21: Sensitivity of SC2 to incineration residue generation…………………………………….25

Table S22: Sensitivity of SC2 to sodium hypochlorite dosage……………………………………….25

Table S23: Sensitivity of SC2 to recovered plastic quantity…………………………………………26

Table S24: Sensitivity of SC2 to transport distance………………………………………………….27

**S6. Material Flow Analysis (Process block diagram)**

Figure S5: Process Block Diagram for the base case scenario (SC1)………………………………..29

Figure S6: Process Block Diagram for the alternative scenario (SC2)……………………………....30

**S7. SDG Mapping**

Table S25: The SDGs and specific targets that are associated with the proposed WtE strategy………………………………………………………………………………………………..31

**S8. Emission Factors**

Table S26: Emission factor for incinerator without scrubber………………………………………...33

Table S27: Emission factor for incinerator with medium energy scrubber…………………………..33

Table S28: Emission factor for diesel fired boiler……………………………………………………34

References…………………………………………………………………………………………….34

**S1. Healthcare Waste Treatment Scenario in PRISM and Literature Review**

Figure S1: PRISM approach for medical waste treatment.

Table S1: Summary of previous studies on healthcare waste life cycle assessment (LCA) and material flow analysis (MFA), with identified research gaps addressed by the present study.

| **Study (Author, Year)** | **Location / Context** | **Method & Tool** | **Scenarios / Scope Compared** | **Key Findings** | **Gap Addressed by This Study** |
| --- | --- | --- | --- | --- | --- |
| Zhao et al., 2009[1] | China | Comparative LCA (ISO 14040), CML1999 baseline; contribution & perturbation analysis | Hazardous waste incineration (HWI, 0/15/30% energy recovery) vs. autoclave + sanitary landfill (AL, 0/10% energy recovery-5 alternatives | HWI, 30% has lowest impact in nearly all categories except freshwater ecotoxicity; incineration dominates GW/FAET, landfill dominates eutrophication (non-incineration ~7× higher; dioxins contribute only ~10% of human toxicity | Compares only two treatment routes (no pyrolysis/microwave); no city-scale MFA, no cost analysis, no plastic-recycling stream |
| Soares et al., 2013[2] | Brazil, small HCW generators | Lab disinfection-efficacy testing + LCA (ReCiPe Endpoint H/A, SimaPro) + LCC + eco-efficiency analysis | Microwave vs. autoclave vs. lime disinfection, all followed by landfill | Microwave disinfection most eco-efficient for small-volume generators. | Limited to a single disinfection unit, not a full gate-to-gate facility. |
| Hong et al., 2018[3] | Southeastern China | Cost-coupled LCA (SDU method: ReCiPe + IMPACTWorld+ and USEtox) + LCC + Monte Carlo uncertainty analysis | Pyrolysis vs. chemical disinfection vs. steam sterilization | Steam sterilization had highest environmental & economic burden; chemical disinfection lowest on both; pyrolysis intermediate; but adding energy recovery (355 kWh/t) would make pyrolysis comparable to chemical disinfection | Disinfection-only technologies studied in isolation; no integration of WtE/material recovery into a single system boundary; no district/city-scale MFA |
| Ahmad et al., 2019[4] | Swat District, Pakistan (12 government + private hospitals) | Field data collection (surveys/interviews) + consequential LCA (CML baseline, OpenLCA/Ecoinvent) | Scenario A (incineration + landfill) vs. Scenario B (direct landfill) vs. Scenario C (pyrolysis + chemical disinfection + recycling) | Current practices (A, B) far worse across all 8 impact categories; incineration's human toxicity impact is largest of all; Scenario C recovers 78% of sharps and 41% of plastic/paper | District-level scope only; no formal MFA–LCA integration; recycling/plastic valorization only roughly estimated, not quantitatively modeled |
| Deepak et al., 2022[5] | Garhwal, Uttarakhand, India | LCA (SimaPro 9.1.0.4, CML-IA, baseline v3.05) | Baseline integrated system (incineration + autoclave and shredder + chemical disinfection + ETP) vs. same system with energy recovery (ER) from incineration | Scenario 2 (with ER) outperformed baseline in all 11 impact categories: GWP decreased by 9.72%, human toxicity decreased by 10.85%, marine aquatic ecotoxicity decreased by 13.31%, photochemical oxidation by 12.81%, eutrophication by 11.38%; FAE and MAE together contributed ~96% of overall impact. | 2.5-ton functional unit; no enhanced plastics recycling modelling, only added electricity generation for the alternative scenario without considering incineration bottom ash impact, no heat recovery scheme, Ecoinvent background data not representative of actual Indian conditions; no valorization of sterilized/recycled waste materials modelled |
| Alam & Mosharraf, 2020[6] | Chittagong, Bangladesh | Preliminary LCA (SimaPro 7) | Four scenarios, three existing HCWM systems (by HCE, private initiative, City Corporation) vs. one proposed standard system (disinfection + incineration with ER + sanitary landfill) | First LCA-based study on Bangladeshi HCW; open burning/incineration drove GW and HT, dumping/landfilling drove FWAET and TET; proposed scenario showed markedly lower impacts across all four categories | Only 4 impact categories assessed; no country-specific LCA database; poor data record-keeping; excludes drugs, radioactive/chemical waste and pathogen transmission; no material flow analysis or quantified WtE modelling |
| Dihan et al., 2023[7] | Bangladesh (national) | Narrative review | HCW generation, COVID-19 impact, circular-economy framework | Established the case for quantitative LCA-based sustainability assessment. | Review only —lack of an actual LCA/MFA study. |
| Allesch & Brunner, 2017[8] | Austria | Material Flow Analysis (methodological) | General MSW management system | Demonstrated MFA as a decision-support tool for waste-system optimization. | Not HCW-specific; not paired with LCA. |
| Çetin et al. (2025)[9] | Istanbul, Türkiye | LCA: GaBi Education v7.3, CML 2001 (6 impact categories) | Baseline vs. 4 scenarios varying incineration/sterilization/landfill ratios ± waste segregation/minimization | Scenario 4 (segregation + incineration + sterilization + landfill) had lowest overall impact (14.21%); higher incineration share worsened acidification/toxicity; segregation reduced medical waste volume needing treatment but increased landfill-bound MSW | Comparable megacity/developing-context motivation, but only 6 impact categories, no plastic recycling/WtE energy recovery scheme, no MFA integration, no sensitivity analysis, no hotspot analysis, no explicit SDG framework. |
| Cho et al. (2024)[10] | Seoul, South Korea | Gate-to-grave LCA — SimaPro, Ecoinvent 3.8, ReCiPe endpoint method (6 impact categories) | Hazardous waste incineration (HW) vs. general waste landfill with segregation (GW) vs. plastic recycling with segregation (PR); Functional unit = 1 kg medical device plastic packaging waste (MDPW) | GW reduced GWP by 79.7% and PR to 11.8% relative to HW baseline; GHG emissions were 5.26, 4.19, and 0.62 kg CO_2_-eq for HW, GW, and PR respectively; PR gave net-positive credits (fossil resource scarcity -6%) by offsetting virgin plastic production; PET/PS/PETG identified as most impactful polymer types; segregation alone (before recycling) cut ozone depletion 5–78% and PM formation 26–60% | Narrow waste-stream focus (single-use plastic packaging only, one department/hospital), no MFA, no city-wide generation scale, no WtE/energy-recovery scheme, no integration with infectious-waste treatment technologies (autoclaving, chemical disinfection, incineration mix), only 6 impact categories, no sensitivity analysis, no explicit circular-economy/SDG-linked scenario modeling at city scale |
| Zlaugotne et al. (2025)[11] | Latvia | Gate-to-gate LCA, SimaPro v9.5, ReCiPe 2016 (Midpoint, Hierarchist), 18 midpoint indicators | BAU (landfill) vs. 6 valorization scenarios (asphalt, cement, RDF, syngas, acoustic panels, hydroponic plastics) + combined VS7 | Energy-recovery scenarios (RDF, syngas) gave largest environmental credits; material-recovery scenarios (hydroponic plastics, asphalt) best among recycling options; landfilling universally worst; transport/treatment impacts similar across scenarios | Valorization-product focus (not integrated with treatment technology selection), no hotspot analysis of upstream treatment process, no developing-country infrastructure constraints, no sensitivity analysis on treatment parameters, no SDG linkage |
| Nematollahi et al., 2024[12] | Isfahan, Iran | LCA: IWM-2 model + Ecoinvent, SimaPro 9, TRACI 2014 (10 impact categories) | 4 non-infectious scenarios (landfill, composting, incineration, recycling combinations) + 2 infectious scenarios (pyrolysis vs. chemical disinfection) | Chemical disinfection had lower GWP than pyrolysis (859.9 vs 1483.2 kg CO_2_ eq/ton); composting + material recovery minimized ecotoxicity; incineration + recycling best for acidification/eutrophication/ozone depletion. | Single-division/city focus, only 10 impact categories, no sensitivity analysis, functional unit of 1 t (not full city generation), no WtE heat recovery or plastic recycling integration, no SDG linkage |
| Duane et al., 2026[13] | Modena, Italy | Process-based LCA: OpenLCA v2.0, Ecoinvent v3.9.1, EF 3.1/PEF method, Monte Carlo + extensive sensitivity analysis (17 parameters) | Full incineration vs. 90% pyrolysis + 10% residual incineration | Pyrolysis pathway net −230 kg CO_2_-eq vs. +1996 kg CO_2_-eq for incineration; credits in 7/16 categories (non-renewable energy, GWP, freshwater eutrophication, photochemical oxidants); results highly sensitive to ethylene yield and electricity source | Waste-stream-specific (dialysis plastics only), single clinical site, no MFA, no city-scale generation data, no integration with autoclaving/chemical disinfection/landfill mix typical of developing-country HCW systems |

**S2.** **Life Cycle Inventory Analysis**

Table S2: Background life cycle inventory datasets used for both scenarios, taken from the ecoinvent 3.9.1 database as implemented in openLCA 2.6.2. All datasets are unit process (U) datasets of the cut-off system model.

| **System boundary process** | **Provider name in the ecoinvent database** |
| --- | --- |
| Diesel | market for diesel \| diesel \| Cutoff, U - RoW |
| Transport, HDT | market for transport, freight, lorry 16-32 metric ton, EURO3 \| transport, freight, lorry 16-32 metric ton, EURO3 \| Cutoff, U - RoW |
| Transport, LDT | market for transport, freight, lorry 7.5-16 metric ton, EURO4 \| transport, freight, lorry 7.5-16 metric ton, EURO4 \| Cutoff, U - RoW |
| Electricity | market for electricity, medium voltage \| electricity, medium voltage \| Cutoff, U - BD |
| Tap water | market for tap water \| tap water \| Cutoff, U - RoW |
| Sodium hypochlorite | market for sodium hypochlorite, without water, in 15% solution state \| sodium hypochlorite, without water, in 15% solution state \| Cutoff, U - RoW |
| Deionised water | market for water, deionised \| water, deionised \| Cutoff, U - RoW |
| Hydrated lime, scrubber reagent | market for lime, hydrated, loose weight \| lime, hydrated, loose weight \| Cutoff, U - RoW |
| Fly ash and scrubber sludge disposal | treatment of fly ash and scrubber sludge, hazardous waste incineration \| fly ash and scrubber sludge \| Cutoff, U - RoW |
| Mixed plastic disposal | treatment of waste plastic, mixture, open dump, wet infiltration class (500 mm) \| waste plastic, mixture \| Cutoff, U - GLO |
| Avoided virgin PET | market for polyethylene terephthalate, granulate, amorphous \| polyethylene terephthalate, granulate, amorphous \| Cutoff, U - GLO |
| Avoided virgin HDPE | market for polyethylene, high density, granulate \| polyethylene, high density, granulate \| Cutoff, U - GLO |
| Avoided virgin PP | market for polypropylene, granulate \| polypropylene, granulate \| Cutoff, U - GLO |
| Avoided virgin PVC | market for polyvinylchloride, bulk polymerized \| polyvinylchloride, bulk polymerized \| Cutoff, U - GLO |
| Incineration residue disposal^[1]^ | treatment of average incineration residue, residual material landfill \| average incineration residue \| Cutoff, U - RoW |

Table S3*:* Inventory analysis for the incinerator

| **Unit** | **Item** | | **Unit** | **Base scenario** | **Alternative scenario** |
| --- | --- | --- | --- | --- | --- |
| Incinerator | Input | Diesel Consumed | gallon (US) | 5.28 | 41.93 |
|  |  | Electricity Consumed^[2]^ | kWh | 16.44 | 3232.24 |
|  |  | Hydrated lime, Ca(OH)_2_^[3]^ | kg | - | 960.0 |
|  |  | Waste incinerated | ton | 5.54 | 42.13 |
|  | Output | Electricity Recovered (Gross)^[4]^ | kWh | - | 20572.28 |
|  | Air Emission | CO_2_ , total^[5]^ | ton | 9.71 | 53.73 |
|  |  | CO_2_, fossil^[6]^ | ton | 5.59 | 26.70 |
|  |  | NO_x_ | kg | 8.98 | 68.42 |
|  |  | CO | kg | 7.42 | 56.47 |
|  |  | SO_2_ | kg | 5.56 | 7.98 |
|  |  | SO_3_ | g | 1.44 | 11.41 |
|  |  | Pb | kg | 0.18 | 0.03 |
|  |  | Hg | kg | 0.27 | 0.59 |
|  |  | CH_4_ | kg | 0.0022 | 0.017 |
|  |  | Hydrogen Chloride (HCl) | kg | 84.08 | 53.87 |
|  |  | Total Particulate Matter (PM) | kg | 11.72 | 3.12 |
|  |  | Polychlorinated dibenzo-p-dioxins, PCDD | mg | 0.054 | 0.04 |
|  |  | Polychlorinated dibenzofurans, PCDF | mg | 0.18 | 0.09 |
|  | Solid Waste | Bottom ash to landfill | ton | 1.11 | 4.28 |
|  |  | Fly ash and scrubber sludge^[7]^ | kg | - | 1314.00 |

Table S3A: SEM–EDX elemental composition of medical-waste incinerator bottom ash at selected spots and over an analyzed area.

| **Element** | **Spot 1 (wt.%)^[8]^** | **Spot 2 (wt.%)^8^** | **Area analysis (wt.%)^[9]^** | **Error, area (%)** |
| --- | --- | --- | --- | --- |
| Al | — | 14.76 | n.q. | — |
| Si | — | 14.86 | n.q. | — |
| Ca | 67.00 | 46.17 | n.q. | — |
| Cr | 2.73 | 1.94 | 3.68 | 31.26 |
| Ni | 4.20 | 2.51 | 4.22 | 40.63 |
| Cu | 3.09 | 2.52 | 4.68 | 40.95 |
| Zn | 2.80 | 3.60 | 6.73 | 25.03 |
| As | 0.14 | 0.12 | 0.02 | 99.99 |
| Cd | 1.17 | 1.82 | 12.06 | 6.82 |
| Hg | 14.64 | 10.33 | 20.43 | 51.62 |
| Pb | 4.21 | 1.36 | 48.16 | 7.51 |
| Mo | 0.02 | — | n.q. | — |

*Note:* Values represent localized measurements and should not be interpreted as bulk concentrations of the bottom ash. Elements with error exceeding approximately 25 per cent are semi-quantitative and are reported for identification rather than quantification. Conditions: 20 kV, take-off angle 36.5°, live time 358.4 s, detector resolution 129.8 eV.

**Figure S2:** SEM–EDX characterization of the medical-waste incinerator bottom ash. **(a)** SEM image showing the ash particles and the location of **Spot 1** on a darker particle. **(b)** The same area showing the location of **Spot 2** on a brighter particle. **(c)** Elemental map of the analyzed area, showing the distribution of the detected elements. **(d)** Lead (Pb) map showing that Pb is concentrated mainly around the brighter particles. The scale bars represent 100 µm. EDX analysis was performed at 20 kV with a 36.5° take-off angle and 587× magnification.

Table S4: Inventory analysis data for transportation.

| **Unit** | **Item** | | **Unit** | **Base scenario** | **Alternative scenario** |  |
| --- | --- | --- | --- | --- | --- | --- |
| Transportation by PRISM Van, Light Duty Truck | Input | Diesel | litres | 151.25 | 720.24 | |
|  |  | Total no. of trucks | no. | 11.00 | 52.00 | |
|  |  | Distance traveled by all trucks | km | 849.97 | 4018.04 | |
|  |  | Average load per truck | ton | 0.89 | 0.89 | |
|  |  | Transport activity | t.km | 751.6 | 3576.4 | |
| Transportation by DNCC/DNSC, Heavy Duty Truck | Input | Diesel | litres | 685.50 | - | |
|  |  | Total No. of Trucks | no. | 30.00 | - | |
|  |  | Distance Traveled by all trucks | km | 2318.10 | - | |
|  |  | Average load per truck | ton | 1.22 | - | |
|  |  | Transport activity | t.km | 2827.40 | - | |

*Note:* In the base scenario (SC1), general medical waste is collected by the city corporation using HDTs. In the alternative scenario (SC2), the entire waste stream is collected by PRISM using LDTs, eliminating the need for HDT collection. However, the LDT-based system has higher emissions per t.km, resulting in a transport penalty.

Table S5: Inventory data for the boiler and autoclave.

| **Unit operation** | **Item** | | **Unit** | **Base case** | **Alternative case** |
| --- | --- | --- | --- | --- | --- |
| Boiler and Autoclave | Input | BFW/Makeup water | liters | 400.00 | 400.00 |
|  |  | Electricity Consumed | kWh | 9.23 | 9.23 |
|  |  | Diesel | gallon (US) | 5.02 | - |
|  | Air Emission | SO_2_ | g | 96.92 | - |
|  |  | SO_3_ |  | 1.36 | - |
|  |  | NO_x_ |  | 45.50 | - |
|  |  | CO |  | 11.38 | - |
|  |  | PM |  | 4.55 | - |
|  |  | CO_2_ |  | 51279.99 | - |
|  |  | CH_4_ |  | 2.05 | - |
|  |  | N_2_O |  | 0.40 | - |

*Note:* In the base scenario (SC1), autoclave steam is supplied by a dedicated diesel-fired boiler. In the alternative scenario (SC2), **heat integration** is applied by recovering low-pressure exhaust steam from the waste-to-energy plant to meet the autoclave steam demand, eliminating fuel combustion and associated emissions at the autoclave.

Table S6: Emissions from landfill and open dumping of plastic waste.

| **Waste Disposal Technology/ Unit Processes** | **Item** | | **Unit/day** | **Base case** | **Alternative case** |
| --- | --- | --- | --- | --- | --- |
| Landfill Disposal | Input | General Waste Disposed | ton | 36.59 | - |
|  | Gas Emission^[10]^ | CH_4_, biogenic | ton | 1.98 | - |
|  |  | CO_2_, biogenic | ton | 4.88 | - |
|  |  | N_2_ | ton | 0.21 | - |
|  |  | O_2_ | ton | 0.08 | - |
|  |  | Sulfides | ton | 0.04 | - |
|  |  | NH_3_ | ton | 0.0130 | - |
|  |  | H_2_ | ton | 0.0005 | - |
|  |  | CO | ton | 0.0072 | - |
|  | Water Emission | COD^[11],^^[12]^ | kg | 59.10 | - |
|  |  | T-N^11,12^ | g | 155.74 | - |
|  |  | T-P^11,12^ | g | 245.90 | - |
|  |  | Cu^[13]^ | g | 2.20 | - |
|  |  | Cr^13^ | g | 5.12 | - |
|  |  | Zn^13^ | g | 12.44 | - |
|  |  | Pb^13^ | g | 1.50 | - |
|  |  | Cd^13^ | g | 0.44 | - |
|  |  | Ni^13^ | g | 5.42 | - |
| Open dumping | Solid Waste | Mixed Plastic | ton | 2.96 | 1.86 |

Table S7: Electricity consumption of different equipment for the base and alternative scenarios

| **Unit** | **Equipment** | **Power (kW)** | | **Time of operation (h)** | | **Energy consumption (kWh)** | |
| --- | --- | --- | --- | --- | --- | --- | --- |
|  |  | **Base** | **Alternative** | **Base** | **Alternative** | **Base** | **Alternative** |
| Chemical Disinfection | Pump | 0.746 | 0.746 | 1.0 | 1.0 | 0.746 | 0.746 |
|  | Mixer | 1.5 | 1.5 | 1.0 | 1.0 | 1.5 | 1.5 |
|  | Ancillary^[14]^ | 0.5 | 0.5 | 2.0 | 2.0 | 1.0 | 1.0 |
|  | Subtotal | | | | | 3.2 | 3.2 |
| Shredding | Shredder | 3.0 | 7.5 | 6.0 | 9.0 | 18.0 | 67.5 |
|  | Feed conveyor^[15]^ | 0.0 | 0.75 | 0.0 | 9.0 | 0.0 | 6.75 |
|  | Ancillary | 0.5 | 0.5 | 6.0 | 9.0 | 3.0 | 4.5 |
|  | Subtotal | | | | | 21.0 | 78.75 |
| Autoclave | Pump | 0.746 | 0.746 | 5.0 | 5.0 | 3.73 | 3.73 |
|  | Vacuum pump | 1.5 | 1.5 | 2.0 | 2.0 | 3.0 | 3.0 |
|  | Ancillary | 0.5 | 0.5 | 5.0 | 5.0 | 2.5 | 2.5 |
|  | Subtotal | | | | | 9.2 | 9.2 |
| Incinerator | Slurry Pump | 0.0 | 6.0 | 0.0 | 12.0 | 0.0 | 72.0 |
|  | Blower | 0.37 | 3.7 | 12.0 | 12.0 | 4.4 | 44.4 |
|  | Charging and ash conveyor^15^ | 0.0 | 1.5 | 0.0 | 12.0 | 0.0 | 18.0 |
|  | Ancillary | 1.0 | 1.0 | 12.0 | 12.0 | 12.0 | 12.0 |
|  | Parasitic load, WtE^[16]^ | - | - | - | - | 0.0 | 3,085.8 |
|  | Subtotal | | | | | 16.440 | 3,232.2 |
| Total | | | | | | 49.92 | 3323.5 |

**S3. Field Survey and Literature Data**

Table S8: Survey Data and Questionnaire

| **Section** | **Parameter** | **Data Entry** |
| --- | --- | --- |
| **1. Collection** | Total amount of waste generated from HCFs | 45-50 ton/day |
|  | Amount of General Waste collected by DSCC and DNCC | 35-42 ton/day |
|  | Type and quantity of packaging materials used for collection | N/A |
|  | Packaging waste generated after collection | N/A |
| **2. Transportation** | Number of vehicles used by prism | 11 |
|  | Amount of waste carried | 1-1.5 ton |
|  | Engine fuel requirement | 12.5-15 L/day/truck |
|  | Emissions generated | N/A |
| **3. Segregation** | Waste composition | YES |
|  | Conveyor belt electricity requirement (if any) | No |
| **4. Autoclave** | Total amount of waste sent to autoclaving | 800-1000 kg |
|  | Steam flow rate | 150 L/day/ton |
|  | Steam temperature and pressure | 121^o^C, 1.1bar |
|  | Source of water | Deep Tubewell |
|  | Boiler fuel consumption | 18-20 L diesel/hr |
|  | Boiler type | Horizontal Boiler |
| **5. Chemical Disinfection** | Amount of plastics to be disinfected | 2-2.5 ton/day |
|  | Water requirement | 2000 L |
|  | Category of the plastics | Noninfectious |
|  | Pumping and mixing energy requirement | 3-5 HP |
|  | Recycling amount | 400-420 kg/day |
|  | Chemical (Sodium Hypochlorite) | 50 kg/day |
| **6. Shredding Process** | Amount of materials to be shredded | 400-450 kg |
|  | Electricity requirement for shredding | 3kW |
| **7. Recycling** | Total amount recycled (plastics) | 400-420 kg/day |
|  | Amount according to type of materials | N/A |
| **8. Incineration** | Amount of waste incinerated | 5.54 ton/day |
|  | Size of the incinerator | N/A |
|  | Capacity of the incinerator | 320 kg/batch |
|  | Fuel requirement | 18-20 L/hr |
|  | Electricity requirement | 17 kWh |
|  | Emission Data | N/A |
|  | Energy recovery (if any) | No |
|  | Temperature range of incineration | 600-900 °C |
| **9. Deep Burial** | Amount and composition of waste disposed | 0.8 ton/day |
|  | Disposing area | - |
|  | Type of waste disposed | Organic (Pathological) |

Table S9: PRISM-provided plastic waste sample (Source: Field visit)

.

| **Item no.** | **Medical kit name** | **Material** | **Weight in sample (g)** |
| --- | --- | --- | --- |
| **1** | Syringe barrel and plunger | Polypropylene | 63.66 |
| **2** | Syringe rubber | Misc | 15.52 |
| **3** | IV bag and tubing | PVC | 116.96 |
| **4** | Blood collection tube | PET | 21.97 |
| **5** | Disposable specimen cup | Polypropylene | 15.23 |
| **6** | Hexanols type bottle | HDPE | 95.12 |
| **7** | Saline bag | Polypropylene | 43.66 |
| **8** | Soft saline bag | LDPE | 59.14 |
| **9** | Cap of blood sample tube | LDPE | 7.84 |
| **10** | Pregnancy kit | Polypropylene | 23.00 |
| **11** | Plastic test tube | Polypropylene | 18.2 |
| **12** | Nasal drop type bottle | HDPE | 11.45 |
| **13** | PET bottle | PET | 31.48 |
| **14** | Multiple mixed PP | Polypropylene | 69.45 |

Figure S3: Different noninfectious plastic compositions found in medical waste

Table S10: Maximum recyclable quantity for each type of plastic. Reprocessing potential is obtained from the literature. [18].

| **Plastic** | **Non-infectious Quantity (ton/day)** | **Reprocessing potential** | **Recyclable quantity (ton/day)** |
| --- | --- | --- | --- |
| PET | 0.22 | 80% | 0.18 |
| HDPE | 0.44 | 73% | 0.32 |
| PP | 0.96 | 79% | 0.76 |
| PVC | 0.48 | 56% | 0.27 |
| LDPE | 0.28 | 0% | 0.00 |
| Misc | 0.06 | 0% | 0.00 |
| Total | 2.44 | - | 1.53 |

**S4. Detailed Calculation**

**S4.1 Calculation of MWGR (kg/day/bed) from Empirical Correlation**

Table S11: Human development index (HDI), life expectancy at birth (LE), mean years of schooling (MYS) and CO_2_ emissions per capita (CE) of Bangladesh from 2010 to 2019[19], [20]. MWGR calculated by the three models is also presented. Values from 2020 to 2025 are extrapolated.

| **Year** | **HDI** | **LE at birth (Years)** | **MYS (Years)** | **CE**  **(Ton/capita)** | **MWGR (kg/bed/day)** | | |
| --- | --- | --- | --- | --- | --- | --- | --- |
|  |  |  |  |  | **Model-1** | **Model-2** | **Model-3** |
| 2010 | 0.557 | 69.9 | 5.3 | 0.343 | 1.085 | 0.935 | 0.784 |
| 2011 | 0.566 | 70.26 | 5.7 | 0.364 | 1.096 | 0.955 | 0.842 |
| 2012 | 0.575 | 70.61 | 5.8 | 0.384 | 1.108 | 0.974 | 0.861 |
| 2013 | 0.579 | 70.93 | 5.5 | 0.397 | 1.116 | 0.984 | 0.825 |
| 2014 | 0.579 | 71.23 | 5.2 | 0.413 | 1.125 | 0.988 | 0.791 |
| 2015 | 0.595 | 71.51 | 5.8 | 0.462 | 1.144 | 1.026 | 0.882 |
| 2016 | 0.606 | 71.79 | 5.9 | 0.471 | 1.151 | 1.046 | 0.898 |
| 2017 | 0.616 | 72.05 | 6.1 | 0.497 | 1.163 | 1.068 | 0.931 |
| 2018 | 0.625 | 72.32 | 6.1 | 0.517 | 1.173 | 1.087 | 0.937 |
| 2019 | 0.632 | 72.59 | 6.2 | 0.556 | 1.189 | 1.109 | 0.961 |
| 2020 | 0.639 | 72.94 | 6.2 | 0.568 | 1.197 | 1.123 | 0.964 |
| 2021 | 0.648 | 73.24 | 6.3 | 0.591 | 1.209 | 1.143 | 0.983 |
| 2022 | 0.656 | 73.53 | 6.4 | 0.614 | 1.220 | 1.162 | 1.003 |
| 2023 | 0.665 | 73.83 | 6.5 | 0.637 | 1.231 | 1.182 | 1.022 |
| 2024 | 0.673 | 74.12 | 6.6 | 0.661 | 1.243 | 1.201 | 1.042 |
| 2025 | 0.681 | 74.42 | 6.7 | 0.684 | 1.254 | 1.220 | 1.061 |

Table S12: Total Bed No. Count Under DNCC and DSCC[21].

| **Category** | **Bed No** |
| --- | --- |
| DNCC Public | 7200 |
| DNCC Private | 15467 |
| DSCC Public | 4800 |
| DSCC Private | 9466 |
| Total | 36933 |

**S4.2 Estimation of Power Generation in the Alternative Scenario (SC2) by Energy Balance**

Electricity generation in the alternative scenario was estimated by an energy balance on the incinerator and steam cycle, so that each assumption is stated explicitly and can be tested. The net calorific value available for conversion is the lower heating value of the waste less the energy consumed in evaporating its moisture content. Gross electrical output follows as the product of the mass flow rate, the net calorific value, and the overall thermal-to-electrical conversion efficiency.

$$\text{P}\text{=G×}\left( \text{LHV-}\text{m}_{\text{c}}\text{×2.26} \right)\text{×}\text{η}_{\text{E}}$$

Where, P is electrical power, G is the mass flow rate of waste in kilograms per second, LHV is the lower heating value in megajoules per kilogram, m is the moisture mass fraction, 2.26 megajoules per kilogram is the latent heat of vaporisation of water, and $\eta_{E}$ is the overall conversion efficiency, comprising boiler, turbine cycle, and generator efficiencies combined.

Table S13: Parameters used in the estimation of power generation in the alternative scenario.

| **Parameter** | **Value** | **Basis** |
| --- | --- | --- |
| Mass flow rate of waste, G | 0.975 kg/s | 42.134 t/day over 12 hours of operation |
| Lower heating value, LHV | 10.00 MJ/kg | Weighted from waste composition analysis |
| Moisture mass fraction, m | 0.54 | Weighted from waste composition analysis |
| Latent heat of vaporisation | 2.26 MJ/kg | Physical constant at atmospheric pressure |
| Net calorific value | 8.78 MJ/kg | LHV less moisture evaporation penalty |
| Overall conversion efficiency, $\text{η}$ | 20% | Within the range of 14 to 28 per cent reported for conventional incineration plants[22] |
| Hours of operation | 12 h/day | Batch operation schedule of the facility |
| Gross electricity generated | 20,572.28 kWh/day | Calculated |
| Parasitic load | 3,085.84 kWh/day | 15 per cent of gross generation |

**S4.3 Estimation of Methane Emissions from Landfill**

Estimating greenhouse gas (GHG) emissions from landfills is challenging, especially in regions with limited data, like the medical waste landfills managed by PRISM Bangladesh. The IPCC provides methods such as the Zero-Order Decay Model (ZODM) and the First-Order Decay Model (FODM). While ZODM is simple and requires less data, it assumes all methane is generated in the same year the waste is dumped, leading to potential overestimations. Moreover, it doesn't consider the surface oxidation and diffusion of the generated methane[23], [24], [25]. FODM is more accurate because it accounts for the gradual breakdown of waste over time. This is important for medical waste in PRISM-managed landfills, where waste decomposes progressively. It provides a more realistic methane emission profile from the landfills, considering the time dependency of the waste degradation, and breaks down the emissions rate over time[23], [26], [27]. This model requires adequate information about waste properties, historical data, and management practices[28].

The model equation for the FODM is[24] :

$$\text{Q}_{\text{T,x}}\text{=A×k×}\text{MSW}_{\text{T(x)}}\text{×}\text{MSW}_{\text{F(x)}}\text{×}\text{L}_{\text{o}}\text{×}\text{e}^{\text{-k(T-x)}}$$

Table S14: Definition of different terms used in the equation

| **Symbol** | **Definition** | **Formula / Taken Value** |
| --- | --- | --- |
| $\text{Q}_{\text{T,}\text{ }\text{x}}$ | The amount of methane generated in the current year from waste disposed of in the year x. (Gg CH_4_/year) | Range of values of Q_T,x_ using the above equation from 2015 to 2025 |
| T | The current year (year of the emission estimate | A range of values of T will be taken to analyze the methane generation scenario from 2015 to 2025 |
| x | The year of waste disposal. | x= 2015 |
| $\text{MSW}_{\text{T(x)}}$ | Total general MW generated in the year x (Gg) | The total general MW generated in 2015 is taken to be 12,187.01 tonnes/year, estimated from the Model 1 generation rate for 2015 (Table S11) and the hospital bed count (Table S12), assuming 79% of the total HCW to be general waste |
| $\text{MSW}_{\text{F(x)}}$ | Fraction of general MW disposed of at solid waste disposal sites | $\text{MSW}_{\text{F(x)}}$ is taken as 1 for PRISM. |
| $\text{t}_{\frac{\text{1}}{\text{2}}}$ | Half-life of the waste (yr)[29] | $\text{t}_{\frac{\text{1}}{\text{2}}}$ is taken as 3 years since waste is highly degradable in the PRISM landfill. |
| k | Methane generation constant (1/yr) | k=$\frac{\ln\left( \text{2} \right)}{\text{t}_{\frac{\text{1}}{\text{2}}}}$ = 0.231 yr^-1^ |
| A | Equation correction factor. | A=$\frac{\text{(1-e}^{\text{-K}}\text{)}}{\text{k}}$ |
| MCF | Methane correction factor | MCF = 0.8; for unmanaged, deep sites (>5m)[24] |
| DOC | degradable organic carbon (fraction) (Gg C/ Gg SW) | DOC = 0.4A + 0.17B + 0.15C + 0.30D[24]; (Where, A = paper + rags = 12.93 %, B = leaves + hay + straw = 2.50 %, C = fruits and vegetables = 67.65 %, D = wood = 4.20 %)[27] Therefore, DOC= 0.17 |
| $\text{DOC}_{\text{f}}$ | Fraction of DOC dissimilated. Tabasaran’s[30] theoretical equation, $\text{DOC}_{\text{f}}$ = 0.014T + 0.28, where T = temperature, is used to determine the value. The IPCC default value is 0.77 at a temperature of 35 °C, as suggested by Bingemer and Crutzen [31] | $\text{DOC}_{\text{f}}$ = 0.014T + 0.28  $\text{DOC}_{\text{f}}$ is taken to be 0.77, considering the temperature of the landfill pile is 35 °C. |
| F | fraction of CH_4_ in landfill gas | 0.5 [IPCC default] |
| 16/12 | conversion of C to CH_4_ | 1.3333 |
| $\text{L}_{\text{o}}$ | Methane generation potential (Gg CH_4_/Gg waste) [24] | $\text{L}_{\text{o}}\text{=}\text{MCF×DOC×}\text{DOC}_{\text{f}}\text{×F×}\frac{\text{16}}{\text{12}}$ =0.0698 |

Table S15: Estimation of methane emissions by FODM for PRISM Landfill.

| **Year** | **Methane Emission (ton/year)** | **Final Methane Emission (ton/year)** | **Final Methane Emission (ton/day)** |
| --- | --- | --- | --- |
| 2015 | 157.36 | 157.35 | 0.43 |
| 2016 | 125.63 | 282.99 | 0.78 |
| 2017 | 100.73 | 383.72 | 1.05 |
| 2018 | 80.64 | 464.36 | 1.27 |
| 2019 | 64.88 | 529.24 | 1.45 |
| 2020 | 51.87 | 581.11 | 1.59 |
| 2021 | 41.56 | 622.67 | 1.71 |
| 2022 | 33.29 | 655.96 | 1.80 |
| 2023 | 26.67 | 682.63 | 1.87 |
| 2024 | 21.37 | 704.00 | 1.93 |
| 2025 | 17.11 | 721.11 | 1.98 |

| Year | Methane Emission (tonnes/day) |
| --- | --- |
| 2015 | 0.431118 |
| 2016 | 0.775319 |
| 2017 | 1.0513 |
| 2018 | 1.27224 |
| 2019 | 1.44998 |
| 2020 | 1.59209 |
| 2021 | 1.70595 |
| 2022 | 1.79716 |
| 2023 | 1.87023 |
| 2024 | 1.92877 |
| 2025 | 1.97566 |

Figure S4: Graph of methane emissions (ton/day) using the IPCC FODM method

**S5. Comparative Life Cycle Assessment (LCA) Results**

**S5.1 Impact Assessment results**

Table S16: Comparative life cycle impact assessment results for the base and alternative scenarios, per functional unit of 46.32 tonnes of waste managed per day. ReCiPe 2016 v1.03, midpoint, hierarchist perspective.

| **Impact category** | **Unit** | **SC1** | **SC2** | **Change** |
| --- | --- | --- | --- | --- |
| Terrestrial acidification (TAP) | kg SO_2_-Eq | 39.55 | 1.56 | -96.1% |
| Climate change (GWP100) | kg CO_2_-Eq | 74,694.31 | 16,227.05 | -78.3% |
| Freshwater ecotoxicity (FETP) | kg 1,4-DCB-Eq | 1,305.53 | 3,009.29 | +130.5% |
| Marine ecotoxicity (METP) | kg 1,4-DCB-Eq | 1,844.74 | 4,258.51 | +130.8% |
| Terrestrial ecotoxicity (TETP) | kg 1,4-DCB-Eq | 396,125.71 | 807,173.55 | +103.8% |
| Fossil fuel potential (FFP) | kg oil-Eq | 4.085 | -5,239.71 | net credit |
| Freshwater eutrophication (FEP) | kg P-Eq | 2.75 | 5.42 | +97.2% |
| Marine eutrophication (MEP) | kg N-Eq | 5.31 | 2.97 | -44.1% |
| Human toxicity, carcinogenic (HTPc) | kg 1,4-DCB-Eq | 4,739.15 | 17,796.27 | +275.5% |
| Human toxicity, non-carcinogenic (HTPnc) | kg 1,4-DCB-Eq | 38,246.94 | 65,554.59 | +71.4% |
| Ionising radiation (IRP) | kBq Co-60-Eq | 101.515 | 18.310 | -82.0% |
| Agricultural land occupation (LOP) | m^2^a crop-Eq | 965.91 | 28.84 | -97.0% |
| Surplus ore potential (SOP) | kg Cu-Eq | 149.90 | 85.325 | -43.1% |
| Ozone depletion (ODP) | kg CFC-11-Eq | 0.000274 | -0.007 | net credit |
| Particulate matter formation (PMFP) | kg PM_2.5_-Eq | 20.36 | -0.135 | net credit |
| Photochemical oxidant, humans (HOFP) | kg NO_x_-Eq | 15.52 | 48.72 | +213.9% |
| Photochemical oxidant, ecosystems (EOFP) | kg NO_x_-Eq | 15.64 | 47.30 | +202.5% |
| Water consumption (WCP) | m^3^ | 21.914 | -24.997 | net credit |

*Note:* Categories marked as a net credit are those in which the alternative scenario yields a negative net result, meaning that the avoided burdens from energy recovery and material recovery exceed the burdens of treatment. In the base scenario the corresponding values are positive burdens.

**S5.2	Hotspot Analysis**

Table S17: Process contributions (direct + upstream) to impact categories, base scenario (SC1)

| **Impact category** | **Unit** | **Transport** | **Landfill** | **Autoclave** | **Incineration** | **Chem. disinfection** | **Shredding** | **Recycling** |
| --- | --- | --- | --- | --- | --- | --- | --- | --- |
| Terrestrial acidification (TAP) | kg SO_2_-eq | 2.22 | 25.48 | 0.19 | 9.65 | 4.90 | 0.04 | −2.93 |
| Climate change (GWP100) | kg CO_2_-eq | 739.07 | 67,173.80 | 186.50 | 6,058.68 | 1,583.35 | 17.77 | −1,064.85 |
| Freshwater ecotoxicity (FETP) | kg 1,4-DCB-eq | 18.03 | 3.25 | 149.18 | 741.20 | 425.89 | 0.21 | −32.24 |
| Marine ecotoxicity (METP) | kg 1,4-DCB-eq | 31.53 | 4.48 | 201.37 | 1,077.90 | 572.02 | 0.29 | −42.85 |
| Terrestrial ecotoxicity (TETP) | kg 1,4-DCB-eq | 13,295.45 | 0.00 | 45.28 | 379,292.59 | 6,832.19 | 24.44 | −3,364.24 |
| Fossil fuel potential (FFP) | kg oil-eq | 234.74 | 0.00 | 21.82 | 79.11 | 323.40 | 5.78 | −660.77 |
| Freshwater eutrophication (FEP) | kg P-eq | 0.06 | 0.25 | 0.002 | 2.06 | 0.58 | 0.003 | −0.21 |
| Marine eutrophication (MEP) | kg N-eq | 0.02 | 0.05 | 1.56 | 0.007 | 3.70 | 0.000 | −0.03 |
| Human toxicity, carcinogenic (HTPc) | kg 1,4-DCB-eq | 37.07 | 0.12 | 2.63 | 4,649.31 | 96.56 | 0.36 | −46.90 |
| Human toxicity, non-carcinogenic (HTPnc) | kg 1,4-DCB-eq | 588.85 | 103.67 | 4,199.88 | 22,583.40 | 11,428.91 | 5.49 | −663.25 |
| Ionising radiation (IRP) | kBq Co-60-eq | 7.07 | 0.00 | 0.16 | 3.76 | 123.29 | 0.03 | −32.80 |
| Agricultural land occupation (LOP) | m^2^a crop-eq | 26.39 | 899.36 | 2.43 | 12.76 | 36.18 | 0.07 | −11.28 |
| Surplus ore potential (SOP) | kg Cu-eq | 14.53 | 0.00 | 0.34 | 3.02 | 148.16 | 0.06 | −16.21 |
| Ozone depletion (ODP) | mg CFC-11-eq | 128.02 | 0.00 | 10.62 | 40.90 | 1,211.07 | 6.10 | −1,122.41 |
| Particulate matter formation (PMFP) | kg PM_2.5_-eq | 0.97 | 3.12 | 0.07 | 14.68 | 2.70 | 0.02 | −1.19 |
| Photochemical oxidant formation, human health (HOFP) | kg NO_x_-eq | 4.21 | 0.00 | 0.11 | 10.03 | 3.44 | 0.03 | −2.29 |
| Photochemical oxidant formation, ecosystems (EOFP) | kg NO_x_-eq | 4.36 | 0.00 | 0.12 | 10.06 | 3.51 | 0.03 | −2.44 |
| Water consumption (WCP) | m^3^ | 1.11 | 0.00 | 0.05 | 0.65 | 29.40 | 0.03 | −9.34 |

Table S18: Process contributions (direct + upstream) to impact categories, alternative scenario (SC2)

| **Impact category** | **Unit** | **Transport** | **Autoclave** | **Incineration** | **Chem. disinfection** | **Shredding** | **Recycling** | **Energy recovery** |
| --- | --- | --- | --- | --- | --- | --- | --- | --- |
| Terrestrial acidification (TAP) | kg SO_2_-eq | 2.11 | 0.02 | 41.12 | 4.90 | 0.16 | −10.75 | −36.02 |
| Climate change (GWP100) | kg CO_2_-eq | 878.81 | 120.93 | 32,303.44 | 1,438.58 | 66.64 | −3,906.36 | −14,675.00 |
| Freshwater ecotoxicity (FETP) | kg 1,4-DCB-eq | 19.88 | 149.05 | 2,898.05 | 234.97 | 0.80 | −118.27 | −175.20 |
| Marine ecotoxicity (METP) | kg 1,4-DCB-eq | 34.31 | 201.14 | 4,103.20 | 314.38 | 1.08 | −157.19 | −238.42 |
| Terrestrial ecotoxicity (TETP) | kg 1,4-DCB-eq | 13,780.80 | 14.32 | 818,981.97 | 6,831.36 | 91.67 | −12,341.67 | −20,184.90 |
| Fossil fuel potential (FFP) | kg oil-eq | 275.29 | 2.59 | 1,334.40 | 323.40 | 21.68 | −2,423.97 | −4,773.09 |
| Freshwater eutrophication (FEP) | kg P-eq | 0.07 | 0.001 | 8.11 | 0.58 | 0.01 | −0.76 | −2.59 |
| Marine eutrophication (MEP) | kg N-eq | 0.02 | 1.56 | 0.09 | 1.70 | 0.001 | −0.10 | −0.32 |
| Human toxicity, carcinogenic (HTPc) | kg 1,4-DCB-eq | 41.89 | 2.11 | 18,123.06 | 94.08 | 1.34 | −172.07 | −294.13 |
| Human toxicity, non-carcinogenic (HTPnc) | kg 1,4-DCB-eq | 630.31 | 4,195.83 | 61,622.66 | 6,053.82 | 20.60 | −2,433.10 | −4,535.53 |
| Ionising radiation (IRP) | kBq Co-60-eq | 8.38 | 0.02 | 29.50 | 123.29 | 0.10 | −120.33 | −22.66 |
| Agricultural land occupation (LOP) | m^2^a crop-eq | 27.89 | 2.30 | 60.31 | 33.28 | 0.24 | −41.39 | −53.79 |
| Surplus ore potential (SOP) | kg Cu-eq | 15.96 | 0.05 | 31.68 | 148.16 | 0.23 | −59.46 | −51.29 |
| Ozone depletion (ODP) | mg CFC-11-eq | 210.00 | 2.86 | 629.67 | 1,211.07 | 22.87 | −4,130.00 | −5,030.00 |
| Particulate matter formation (PMFP) | kg PM_2.5_-eq | 0.91 | 0.009 | 16.20 | 2.70 | 0.07 | −4.38 | −15.65 |
| Photochemical oxidant formation, human health (HOFP) | kg NO_x_-eq | 3.50 | 0.01 | 76.60 | 3.44 | 0.12 | −8.41 | −26.55 |
| Photochemical oxidant formation, ecosystems (EOFP) | kg NO_x_-eq | 3.66 | 0.02 | 77.22 | 3.51 | 0.13 | −8.96 | −28.27 |
| Water consumption (WCP) | m^3^ | 1.27 | 0.02 | 6.63 | 29.40 | 0.13 | −34.26 | −28.19 |

**S5.3 Normalized Results**

Table S19: Normalised life cycle impact assessment results, expressed in person equivalents using ReCiPe 2016 global normalisation factors.

| **Impact category** | **Normalisation factor** | **SC1 (Pe)** | **SC2 (Pe)** |
| --- | --- | --- | --- |
| Terrestrial acidification (TAP) | 40.981 | 0.965 | 0.038 |
| Climate change (GWP100) | 7,990.408 | 9.348 | 2.031 |
| Freshwater ecotoxicity (FETP) | 25.175 | 51.859 | 119.536 |
| Marine ecotoxicity (METP) | 43.443 | 42.464 | 98.026 |
| Terrestrial ecotoxicity (TETP) | 15,200.311 | 26.060 | 53.102 |
| Fossil fuel potential (FFP) | 980.392 | 0.004 | −5.345 |
| Freshwater eutrophication (FEP) | 0.650 | 4.228 | 8.339 |
| Marine eutrophication (MEP) | 4.618 | 1.150 | 0.643 |
| Human toxicity, carcinogenic (HTPc) | 10.298 | 460.187 | 1,728.078 |
| Human toxicity, non-carcinogenic (HTPnc) | 31,251.842 | 1.224 | 2.098 |
| Ionising radiation (IRP) | 479.917 | 0.212 | 0.038 |
| Agricultural land occupation (LOP) | 6,167.482 | 0.157 | 0.005 |
| Surplus ore potential (SOP) | 120,051.210 | 0.00125 | 0.00071 |
| Ozone depletion (ODP) | 0.060 | 0.005 | −0.118 |
| Particulate matter formation (PMFP) | 25.570 | 0.796 | −0.005 |
| Photochemical oxidant formation, human health (HOFP) | 20.567 | 0.755 | 2.369 |
| Photochemical oxidant formation, ecosystems (EOFP) | 17.749 | 0.881 | 2.665 |
| Water consumption (WCP) | 266.639 | 0.082 | −0.094 |

*Note:* Characterised results are those reported in Table S16 (ecoinvent – ReCiPe 2016 v1.03, midpoint, hierarchist perspective). Normalisation factors are taken from the World (2010) H set supplied with the openLCA LCIA Methods package.

**S5.4 Sensitivity Analysis**

Table S20: Sensitivity of SC2 to gross electricity recovery from incineration.

| **Impact category** | **Unit** | **SC1** | **SC2 (0%)** | **SC2 (−20%)** | **SC2 (+20%)** |
| --- | --- | --- | --- | --- | --- |
| TAP | kg SO_2_-eq | 39.55 | 1.56 | 10.10 | −6.99 |
| GWP100 | kg CO_2_-eq | 74,694.31 | 16,226.60 | 19,708.80 | 12,744.50 |
| FETP | kg 1,4-DCB-eq | 1,305.53 | 3,009.36 | 3,050.93 | 2,967.79 |
| METP | kg 1,4-DCB-eq | 1,844.74 | 4,258.61 | 4,315.19 | 4,202.04 |
| TETP | kg 1,4-DCB-eq | 396,125.71 | 807,173.00 | 811,963.00 | 802,384.00 |
| FFP | kg oil-eq | 4.09 | −5,239.84 | −4,107.24 | −6,372.42 |
| FEP | kg P-eq | 2.75 | 5.42 | 6.03 | 4.80 |
| MEP | kg N-eq | 5.31 | 2.97 | 3.04 | 2.89 |
| HTPc | kg 1,4-DCB-eq | 4,739.15 | 17,796.80 | 17,866.60 | 17,727.00 |
| HTPnc | kg 1,4-DCB-eq | 38,246.94 | 65,555.80 | 66,632.00 | 64,479.60 |
| IRP | kBq Co-60-eq | 101.52 | 18.31 | 23.69 | 12.93 |
| LOP | m^2^a crop-eq | 965.91 | 28.84 | 41.60 | 16.07 |
| SOP | kg Cu-eq | 149.90 | 85.32 | 97.50 | 73.15 |
| ODP | mg CFC-11-eq | 274.30 | −7,090.00 | −5,900.00 | −8,290.00 |
| PMFP | kg PM_2.5_-eq | 20.36 | −0.14 | 3.58 | −3.85 |
| HOFP | kg NO_x_-eq | 15.52 | 48.72 | 55.02 | 42.42 |
| EOFP | kg NO_x_-eq | 15.64 | 47.30 | 54.01 | 40.59 |
| WCP | m^3^ | 21.91 | −25.00 | −18.31 | −31.69 |

Table S21: Sensitivity of SC2 to incineration residue generation.

| **Impact category** | **Unit** | **SC1** | **SC2 (0%)** | **SC2 (−20%)** | **SC2 (+20%)** |
| --- | --- | --- | --- | --- | --- |
| TAP | kg SO_2_-eq | 39.55 | 1.56 | 0.95 | 2.16 |
| GWP100 | kg CO_2_-eq | 74,694.31 | 16,226.60 | 15,919.50 | 16,533.70 |
| FETP | kg 1,4-DCB-eq | 1,305.53 | 3,009.36 | 2,436.76 | 3,581.97 |
| METP | kg 1,4-DCB-eq | 1,844.74 | 4,258.61 | 3,475.87 | 5,041.35 |
| TETP | kg 1,4-DCB-eq | 396,125.71 | 807,173.00 | 806,647.00 | 807,700.00 |
| FFP | kg oil-eq | 4.09 | −5,239.84 | −5,282.04 | −5,197.65 |
| FEP | kg P-eq | 2.75 | 5.42 | 3.83 | 7.01 |
| MEP | kg N-eq | 5.31 | 2.97 | 2.96 | 2.97 |
| HTPc | kg 1,4-DCB-eq | 4,739.15 | 17,796.80 | 14,227.00 | 21,366.50 |
| HTPnc | kg 1,4-DCB-eq | 38,246.94 | 65,555.80 | 56,402.20 | 74,709.40 |
| IRP | kBq Co-60-eq | 101.52 | 18.31 | 15.53 | 21.09 |
| LOP | m^2^a crop-eq | 965.91 | 28.84 | 19.12 | 38.55 |
| SOP | kg Cu-eq | 149.90 | 85.32 | 83.25 | 87.39 |
| ODP | mg CFC-11-eq | 274.30 | −7,090.00 | −7,120.00 | −7,070.00 |
| PMFP | kg PM_2.5_-eq | 20.36 | −0.14 | −0.39 | 0.11 |
| HOFP | kg NO_x_-eq | 15.52 | 48.72 | 47.97 | 49.47 |
| EOFP | kg NO_x_-eq | 15.64 | 47.30 | 46.53 | 48.07 |
| WCP | m^3^ | 21.91 | −25.00 | −25.46 | −24.53 |

Table S22: Sensitivity of SC2 to sodium hypochlorite dosage.

| **Impact category** | **Unit** | **SC1** | **SC2 (0%)** | **SC2 (−20%)** | **SC2 (+20%)** |
| --- | --- | --- | --- | --- | --- |
| TAP | kg SO_2_-eq | 39.55 | 1.56 | 0.58 | 2.53 |
| GWP100 | kg CO_2_-eq | 74,694.31 | 16,226.60 | 15,963.20 | 16,490.00 |
| FETP | kg 1,4-DCB-eq | 1,305.53 | 3,009.36 | 2,993.63 | 3,025.09 |
| METP | kg 1,4-DCB-eq | 1,844.74 | 4,258.61 | 4,237.93 | 4,279.30 |
| TETP | kg 1,4-DCB-eq | 396,125.71 | 807,173.00 | 805,808.00 | 808,539.00 |
| FFP | kg oil-eq | 4.09 | −5,239.84 | −5,304.33 | −5,175.36 |
| FEP | kg P-eq | 2.75 | 5.42 | 5.30 | 5.54 |
| MEP | kg N-eq | 5.31 | 2.97 | 2.95 | 2.98 |
| HTPc | kg 1,4-DCB-eq | 4,739.15 | 17,796.80 | 17,778.40 | 17,815.10 |
| HTPnc | kg 1,4-DCB-eq | 38,246.94 | 65,555.80 | 65,225.20 | 65,886.40 |
| IRP | kBq Co-60-eq | 101.52 | 18.31 | −6.34 | 42.96 |
| LOP | m^2^a crop-eq | 965.91 | 28.84 | 22.66 | 35.01 |
| SOP | kg Cu-eq | 149.90 | 85.32 | 55.70 | 114.95 |
| ODP | mg CFC-11-eq | 274.30 | −7,090.00 | −7,330.00 | −6,850.00 |
| PMFP | kg PM_2.5_-eq | 20.36 | −0.14 | −0.68 | 0.40 |
| HOFP | kg NO_x_-eq | 15.52 | 48.72 | 48.03 | 49.40 |
| EOFP | kg NO_x_-eq | 15.64 | 47.30 | 46.60 | 48.00 |
| WCP | m^3^ | 21.91 | −25.00 | −30.88 | −19.12 |

Table S23: Sensitivity of SC2 to recovered plastic quantity.

| **Impact category** | **Unit** | **SC1** | **SC2 (0%)** | **SC2 (−20%)** | **SC2 (−40%)** |
| --- | --- | --- | --- | --- | --- |
| TAP | kg SO_2_-eq | 39.55 | 1.56 | 3.70 | 5.85 |
| GWP100 | kg CO_2_-eq | 74,694.31 | 16,226.60 | 17,007.90 | 17,789.20 |
| FETP | kg 1,4-DCB-eq | 1,305.53 | 3,009.36 | 3,033.02 | 3,056.67 |
| METP | kg 1,4-DCB-eq | 1,844.74 | 4,258.61 | 4,290.05 | 4,321.49 |
| TETP | kg 1,4-DCB-eq | 396,125.71 | 807,173.00 | 809,642.00 | 812,110.00 |
| FFP | kg oil-eq | 4.09 | −5,239.84 | −4,755.05 | −4,270.26 |
| FEP | kg P-eq | 2.75 | 5.42 | 5.57 | 5.72 |
| MEP | kg N-eq | 5.31 | 2.97 | 2.99 | 3.01 |
| HTPc | kg 1,4-DCB-eq | 4,739.15 | 17,796.80 | 17,831.20 | 17,865.60 |
| HTPnc | kg 1,4-DCB-eq | 38,246.94 | 65,555.80 | 66,042.40 | 66,529.00 |
| IRP | kBq Co-60-eq | 101.52 | 18.31 | 42.38 | 66.44 |
| LOP | m^2^a crop-eq | 965.91 | 28.84 | 37.11 | 45.39 |
| SOP | kg Cu-eq | 149.90 | 85.32 | 97.22 | 109.11 |
| ODP | mg CFC-11-eq | 274.30 | −7,090.00 | −6,270.00 | −5,440.00 |
| PMFP | kg PM_2.5_-eq | 20.36 | −0.14 | 0.74 | 1.62 |
| HOFP | kg NO_x_-eq | 15.52 | 48.72 | 50.40 | 52.08 |
| EOFP | kg NO_x_-eq | 15.64 | 47.30 | 49.09 | 50.89 |
| WCP | m^3^ | 21.91 | −25.00 | −18.15 | −11.30 |

Table S24: Sensitivity of SC2 to transport distance.

| **Impact category** | **Unit** | **SC1** | **SC2 (0%)** | **SC2 (−20%)** | **SC2 (+20%)** |
| --- | --- | --- | --- | --- | --- |
| TAP | kg SO_2_-eq | 39.55 | 1.56 | 1.13 | 1.98 |
| GWP100 | kg CO_2_-eq | 74,694.31 | 16,226.60 | 16,050.90 | 16,402.40 |
| FETP | kg 1,4-DCB-eq | 1,305.53 | 3,009.36 | 3,005.39 | 3,013.34 |
| METP | kg 1,4-DCB-eq | 1,844.74 | 4,258.61 | 4,251.75 | 4,265.47 |
| TETP | kg 1,4-DCB-eq | 396,125.71 | 807,173.00 | 804,417.00 | 809,930.00 |
| FFP | kg oil-eq | 4.09 | −5,239.84 | −5,294.90 | −5,184.79 |
| FEP | kg P-eq | 2.75 | 5.42 | 5.41 | 5.43 |
| MEP | kg N-eq | 5.31 | 2.97 | 2.96 | 2.97 |
| HTPc | kg 1,4-DCB-eq | 4,739.15 | 17,796.80 | 17,788.40 | 17,805.10 |
| HTPnc | kg 1,4-DCB-eq | 38,246.94 | 65,555.80 | 65,429.70 | 65,681.90 |
| IRP | kBq Co-60-eq | 101.52 | 18.31 | 16.63 | 19.99 |
| LOP | m^2^a crop-eq | 965.91 | 28.84 | 23.26 | 34.41 |
| SOP | kg Cu-eq | 149.90 | 85.32 | 82.13 | 88.52 |
| ODP | mg CFC-11-eq | 274.30 | −7,090.00 | −7,130.00 | −7,050.00 |
| PMFP | kg PM_2.5_-eq | 20.36 | −0.14 | −0.32 | 0.05 |
| HOFP | kg NO_x_-eq | 15.52 | 48.72 | 48.02 | 49.42 |
| EOFP | kg NO_x_-eq | 15.64 | 47.30 | 46.57 | 48.03 |
| WCP | m^3^ | 21.91 | −24.99 | −25.25 | −24.74 |

**S6. Material Flow Analysis (Process block diagram)**

Figure S5: Process Block Diagram for the base case scenario (SC1)

Figure S6: Process Block Diagram for the alternative scenario (SC2)

**S7. SDG Mapping**

Table S25: Sustainable Development Goals and specific targets associated with the proposed waste to energy strategy. Contributions flagged as “conditional” indicate that the underlying environmental gain is accompanied by a countervailing burden increase (principally toxicity, ecotoxicity, or photochemical oxidant formation) that must be managed through improved incineration residue treatment and flue gas (NO_x_) control for the SDG benefit to be fully and durably realized.

| **SDG Section** | **Specific Target** | **Connection with Current Study** | **Quantified Contribution of SC2** |
| --- | --- | --- | --- |
| SDG 3: Good Health and Well-being | Target 3.9: Substantially reduce deaths and illnesses from hazardous chemicals and air pollution | Improved segregation and controlled treatment reduce infection risk, uncontrolled disposal, and air-pollution exposure pathways. Contribution is conditional: increased human toxicity burden requires upgraded incineration residue (fly ash/bottom ash) management to fully realize the health benefit. | Terrestrial acidification reduced by 96.06%; particulate matter formation shifts from +20.36 kg PM_2.5_-Eq (SC1) to −0.14 kg PM_2.5_-Eq (SC2, net avoided). However, human toxicity increases: carcinogenic +275.52%, non-carcinogenic +71.40%, net benefit conditional on residue/heavy-metal management. |
| SDG 6: Clean Water and Sanitation | Target 6.3: Improve water quality by reducing pollution | Elimination of landfill leachate reduces nutrient-driven water contamination; recovered energy and recycling credits also reduce net water demand. Contribution is conditional: increased freshwater ecotoxicity indicates leachate/residue handling still needs improvement. | Marine eutrophication reduced by 44.12%; water consumption potential shifts from 21.91 m^3^ (SC1) to −25.00 m^3^ (SC2, net avoided). However, freshwater ecotoxicity increases by 130.50%, conditional on improved residue and leachate handling. |
| SDG 7: Affordable and Clean Energy | Target 7.2 / 7.3: Increase use of alternative energy sources and improve energy efficiency | Heat and electricity recovered from incineration substitute fossil-based grid supply, reducing fossil dependency. This should be understood as fossil-resource substitution and energy-efficiency improvement, not renewable energy generation. | 20,572.28 kWh/day gross electricity recovery (17,486 kWh/day net export). Fossil fuel potential shifts from 4.09 kg oil-Eq (SC1) to −5239.71 kg oil-Eq (SC2, net avoided). |
| SDG 11: Sustainable Cities and Communities | Target 11.6: Reduce the adverse per-capita environmental impact of cities | Reduced land occupation and elimination of the particulate matter burden support low-landfill-dependence urban waste systems, as required in a land-constrained city like Dhaka. Contribution is conditional: increased photochemical oxidant formation requires flue gas (NO_x_) control to protect urban air quality. | Land occupation reduced by 97.01%; particulate matter formation shifts to net avoided (−0.14 kg PM_2.5_-Eq). However, photochemical oxidant formation increases: human health +213.91%, ecosystems +202.46%, conditional on NO_x_ abatement in flue gas treatment. |
| SDG 13: Climate Change | Target 13.2: Integrate climate change measures into national policies and planning | Diversion from landfill avoids methane generation, and energy recovery displaces fossil-based grid electricity, together delivering the strongest and most unconditional sustainability gain among the assessed SDGs. | Global warming potential reduced by 78.28% relative to SC1. |
| SDG 14: Life Below Water | Target 14.1: Reduce marine pollution, particularly from land-based activities | Elimination of the landfill nutrient-leaching pathway reduces nutrient-driven marine pollution. Contribution is conditional: increased marine ecotoxicity indicates a toxicant-loading trade-off from incineration residue that must be managed for a net-positive outcome for marine ecosystems. | Marine eutrophication (nutrient-driven) reduced by 44.12%. However, marine ecotoxicity potential increases by 130.85%, conditional on incineration residue containment and treatment. |

**S8. Emission Factors**

Table S26: Emission factor for incinerator without scrubber [32].

| Pollutant | Emission Factor (lb./ton) |
| --- | --- |
| Nitrogen Oxides (NO_x_) | 3.56 |
| Carbon Monoxide (CO) | 2.95 |
| Sulfur Dioxide (SO_2_) | 2.17 |
| Sulfur Trioxide (SO_3_) | - |
| Lead (Pb) | 0.0728 |
| Mercury (Hg) | 0.107 |
| Hydrogen Chloride (HCl) | 33.5 |
| Total Particulate Matter (PM) | 4.67 |

Table S27: Emission factor for incinerator with medium energy scrubber [32].

| Pollutant | Emission Factor (lb./ton) |
| --- | --- |
| Nitrogen Oxides (NO_x_) | 3.56 |
| Carbon Monoxide (CO) | 2.95 |
| Sulfur Dioxide (SO_2_) | 0.375 |
| Sulfur Trioxide (SO_3_) | - |
| Lead (Pb) | 0.0016 |
| Mercury (Hg) | 0.0307 |
| Hydrogen Chloride (HCl) | 2.82 |
| Total Particulate Matter (PM) | 0.161 |

Table S28: Emission factor for diesel-fired boiler. [34], [33]

| **Emission Factors for Diesel (Distillate Oil) Fired Boilers (<100 MMBtu/hr)** | | |  |
| --- | --- | --- | --- |
| **Pollutant** | **Emission Factor (lb./1000-gal fuel burnt)** | **Notes** |  |
|  |  |  |  |
|  |  |  |  |
| SO_2_ | 142×S | S = sulfur content in % by weight |  |
| SO_3_ | 2 × S | S = sulfur content in % by weight |  |
| NO_x_ | 20 | **-** |  |
| CO | 5 | **-** |  |
| CO_2_^[17]^ | 22538.6 | **-** |  |
| CH_4_^17^ | 0.9 | **-** |  |
| N_2_O^17^ | 0.176 | **-** |  |
| Filterable PM | 2 | **-** |  |

**References**

[1]	W. Zhao, E. Van Der Voet, G. Huppes, and Y. Zhang, ‘Comparative life cycle assessments of incineration and non-incineration treatments for medical waste’, *Int. J. Life Cycle Assess.*, vol. 14, no. 2, pp. 114–121, Mar. 2009, doi: 10.1007/s11367-008-0049-1.

[2]	S. R. Soares, A. R. Finotti, V. Prudêncio Da Silva, and R. A. F. Alvarenga, ‘Applications of life cycle assessment and cost analysis in health care waste management’, *Waste Manag.*, vol. 33, no. 1, pp. 175–183, Jan. 2013, doi: 10.1016/j.wasman.2012.09.021.

[3]	J. Hong, S. Zhan, Z. Yu, J. Hong, and C. Qi, ‘Life-cycle environmental and economic assessment of medical waste treatment’, *J. Clean. Prod.*, vol. 174, pp. 65–73, Feb. 2018, doi: 10.1016/j.jclepro.2017.10.206.

[4]	R. Ahmad *et al.*, ‘LCA of Hospital Solid Waste Treatment Alternatives in a Developing Country: The Case of District Swat, Pakistan’, *Sustainability*, vol. 11, no. 13, p. 3501, Jun. 2019, doi: 10.3390/su11133501.

[5]	A. Deepak, V. Sharma, and D. Kumar, ‘Life cycle assessment of biomedical waste management for reduced environmental impacts’, *J. Clean. Prod.*, vol. 349, p. 131376, May 2022, doi: 10.1016/j.jclepro.2022.131376.

[6]	O. Alam and A. Mosharraf, ‘A preliminary life cycle assessment on healthcare waste management in Chittagong City, Bangladesh’, *Int. J. Environ. Sci. Technol.*, vol. 17, no. 3, pp. 1753–1764, Mar. 2020, doi: 10.1007/s13762-019-02585-z.

[7]	M. R. Dihan *et al.*, ‘Healthcare waste in Bangladesh: Current status, the impact of Covid-19 and sustainable management with life cycle and circular economy framework’, *Sci. Total Environ.*, vol. 871, p. 162083, May 2023, doi: 10.1016/j.scitotenv.2023.162083.

[8]	A. Allesch and P. H. Brunner, ‘Material Flow Analysis as a Tool to improve Waste Management Systems: The Case of Austria’, *Environ. Sci. Technol.*, vol. 51, no. 1, pp. 540–551, Jan. 2017, doi: 10.1021/acs.est.6b04204.

[9]	E. Çetin, İ. A. Esenlikçi Yıldız, Ç. Öz Yaşar, and A. Yulistyorini, ‘Life Cycle Assessment of Medical Waste Management: Case Study for Istanbul’, *Appl. Sci.*, vol. 15, no. 8, p. 4439, Apr. 2025, doi: 10.3390/app15084439.

[10]	Y. Cho *et al.*, ‘Achieving the sustainable waste management of medical plastic packaging using a life cycle assessment approach’, *Heliyon*, vol. 10, no. 19, p. e38185, Oct. 2024, doi: 10.1016/j.heliyon.2024.e38185.

[11]	B. Zlaugotne, A. Zandberga, J. Gusca, and S. N. Kalnins, ‘Environmental Life Cycle Assessment of Healthcare Waste Valorisation Alternatives’, *Environ. Clim. Technol.*, vol. 29, no. 1, pp. 51–67, Jan. 2025, doi: 10.2478/rtuect-2025-0004.

[12]	H. Nematollahi, R. Ghasemzadeh, M. Tuysserkani, M. Aziminezhad, and M. Pazoki, ‘Comparative life cycle assessment of hospital waste management scenarios in Isfahan, Iran: Evaluating environmental impacts and strategies for improved healthcare sustainability’, *Results Eng.*, vol. 24, p. 102912, Dec. 2024, doi: 10.1016/j.rineng.2024.102912.

[13]	B. Duane *et al.*, ‘Healthcare waste: from incineration to pyrolysis: a comparative life cycle assessment of two different healthcare plastic waste management processes’, *BMC Health Serv. Res.*, Jul. 2026, doi: 10.1186/s12913-026-15024-w.

[14]	G. Tchobanoglous, H. Theisen, and S. A. Vigil, *Integrated solid waste management: engineering principles and management issues*. in McGraw-Hill series in water resources and environmental engineering. New York: McGraw-Hill, 1993.

[15]	P. H. Nielsen and M. Hauschild, ‘Product specific emissions from municipal solid waste landfills’, *Int. J. Life Cycle Assess.*, vol. 3, no. 3, p. 158, May 1998, doi: 10.1007/BF02978824.

[16]	M. Jalili Ghazi Zadeh, ‘Feasibility of soil liners usage in sanitary municipal landfills, stipulating leachate leakage control case study: New Tehran landfill in Kahrizak’, Tehran Faculty of Environment, Tehran, Iran, 2006.

[17]	M. A. Abduli and E. Safari, ‘Preliminary Analysis Of Heavy Metals In The Kahrizak Landfill Leachate: A Conceptual Approach’, *Int. J. Environ. Stud.*, vol. 60, no. 5, pp. 491–499, Oct. 2003, doi: 10.1080/0020723032000050394.

[18]	G. Faraca and T. Astrup, ‘Plastic waste from recycling centres: Characterisation and evaluation of plastic recyclability’, *Waste Manag.*, vol. 95, pp. 388–398, Jul. 2019, doi: 10.1016/j.wasman.2019.06.038.

[19]	UNDP, Ed., *The next frontier: human development and the Anthropocene*. in Human development report, no. 2020. New York, NY: United Nations Development Programme, 2020.

[20]	UNDP, *Human Development Report 2021/2022: Uncertain Times, Unsettled Lives: Shaping Our Future in a Transforming World*, 1st ed. in Human Development Report. Bloomfield: United Nations Publications, 2022.

[21]	DGHS, ‘DGHS | Facility Registry’. Accessed: Aug. 15, 2026. [Online]. Available: https://hrm.dghs.gov.bd/public/facility-registry

[22]	M. S. Khan, Q. Huan, M. Yan, M. Ali, O. U. Noor, and M. Abid, ‘A novel configuration of solar integrated waste-to-energy incineration plant for multi-generational purpose: An effort for achieving maximum performance’, *Renew. Energy*, vol. 194, pp. 604–620, Jul. 2022, doi: 10.1016/j.renene.2022.05.127.

[23]	P. Ghosh *et al.*, ‘Assessment of methane emissions and energy recovery potential from the municipal solid waste landfills of Delhi, India’, *Bioresour. Technol.*, vol. 272, pp. 611–615, Jan. 2019, doi: 10.1016/j.biortech.2018.10.069.

[24]	IPCC, ‘Revised 1996 IPCC Guidelines for National Greenhouse Gas Inventories — IPCC’. Accessed: Aug. 15, 2026. [Online]. Available: https://www.ipcc.ch/report/revised-1996-ipcc-guidelines-for-national-greenhouse-gas-inventories/

[25]	C. K. Singh, A. Kumar, and S. S. Roy, ‘Quantitative analysis of the methane gas emissions from municipal solid waste in India’, *Sci. Rep.*, vol. 8, no. 1, p. 2913, Feb. 2018, doi: 10.1038/s41598-018-21326-9.

[26]	A. Kumar and M. P. Sharma, ‘Estimation of GHG emission and energy recovery potential from MSW landfill sites’, *Sustain. Energy Technol. Assess.*, vol. 5, pp. 50–61, Mar. 2014, doi: 10.1016/j.seta.2013.11.004.

[27]	M. Toha and M. M. Rahman, ‘Estimation and prediction of methane gas generation from landfill sites in Dhaka city, Bangladesh’, *Case Stud. Chem. Environ. Eng.*, vol. 7, p. 100302, Jun. 2023, doi: 10.1016/j.cscee.2023.100302.

[28]	M. R. Dihan, T. Naha, and K. Kirtania, ‘Comparative assessment of waste-to-energy scenarios to mitigate GHG emission from MSW in a developing mega city’, *Energy Sustain. Dev.*, vol. 82, p. 101540, Oct. 2024, doi: 10.1016/j.esd.2024.101540.

[29]	I. Sohoo, M. Ritzkowski, Z. A. Sohu, S. Ö. Cinar, Z. K. Chong, and K. Kuchta, ‘Estimation of Methane Production and Electrical Energy Generation from Municipal Solid Waste Disposal Sites in Pakistan’, *Energies*, vol. 14, no. 9, p. 2444, Apr. 2021, doi: 10.3390/en14092444.

[30]	O. Tabasaran, ‘Main research topics in the Department of Waste Management of the University of Stuttgart and a report on current investigations of practical interest concerning landfill gas’, Jan. 1979, Accessed: Aug. 15, 2026. [Online]. Available: https://www.osti.gov/etdeweb/biblio/8476121

[31]	H. G. Bingemer and P. J. Crutzen, ‘The production of methane from solid wastes’, *J. Geophys. Res. Atmos.*, vol. 92, no. D2, pp. 2181–2187, Feb. 1987, doi: 10.1029/JD092iD02p02181.

[32]	US EPA, ‘AP 42, Fifth Edition, Volume I, Chapter 2: Solid Waste Disposal, Section 2.3: Medical Waste Incineration’, U.S. Environmental Protection Agency, 1993 (reformatted 1995). Accessed: Oct. 20, 2026. [Online]. Available: <https://www.epa.gov/air-emissions-factors-and-quantification/ap-42-fifth-edition-volume-i-chapter-2-solid-waste>

[33]	A. Honest, S. Manyele, J. Saria, and J. Mbuna, ‘Assessment of air pollutant emissions from healthcare waste incinerators of different design features’, *Afr. J. Environ. Sci. Technol.*, vol. 14, no. 10, pp. 311–328, Oct. 2020, doi: 10.5897/AJEST2020.2882.

[34]	US EPA, ‘AP 42, Fifth Edition, Volume I, Chapter 1: External Combustion Sources, Section 1.3: Fuel Oil Combustion’, U.S. Environmental Protection Agency, May 2010. Accessed: Oct. 20, 2026. [Online]. Available: <https://www.epa.gov/sites/default/files/2020-09/documents/1.3_fuel_oil_combustion.pdf>

1. For disposal of incineration residue, both scenarios were modelled using the ecoinvent 3.9.1 dataset *treatment of average incineration residue, residual material landfill*, as no site-specific dataset for medical-waste incinerator ash was available. This choice introduces two important limitations:

   First, the ash composition represented in the ecoinvent dataset corresponds to average municipal solid-waste incineration (MSWI) residue and may not reflect the composition of ash generated at the study facility. SEM–EDX analysis of the actual bottom ash (Table S3A, Figure S2) shows a calcium-rich mineral matrix along with localized enrichment of Pb, Hg, Cd, Zn, Cu, Ni, and Cr. The substantial variation between the analyzed regions also indicates that the ash is heterogeneous. Therefore, these measurements represent selected particle locations rather than the bulk ash and were used only as supporting characterization of the actual residue, rather than as a direct replacement for the ecoinvent inventory.

   Second, the selected ecoinvent dataset assumes cement stabilization of the ash before landfilling. This assumption is appropriate for the alternative scenario, in which cement stabilization is part of the proposed ash-management strategy, but it does not reflect the base case, where the ash is landfilled without stabilization. Applying the same dataset to both scenarios therefore gives SC1 a benefit from cement stabilization that is not actually achieved in the existing practice. As a result, the toxicity impacts of SC1 in **FETP, METP, HTPc and HTPnc** are likely underestimated. If SC1 were modelled without cement stabilization, its impacts in these categories would be expected to increase, thereby reducing the difference between the two scenarios reported in this study.
2. For equipment-level electricity consumption, see Table S7.
3. Reagent demand was calculated stoichiometrically from the mass of HCl and SO₂ removed, taken as the difference between the uncontrolled and scrubbed emission factors applied to the throughput, and multiplied by a dosing factor of 1.5 to allow for practical excess.
4. For the energy balance used to estimate gross electricity generation, see Table S13.
5. Calculated by carbon balance from the mass of waste incinerated.
6. The total was apportioned between fossil and biogenic carbon using the waste composition, with plastics, sharps and the synthetic textile fraction treated as fossil and paper, natural textiles, food waste and other organics treated as biogenic. CO₂ from auxiliary diesel combustion is entirely fossil.
7. Calculated as the sum of reaction products from acid gas neutralisation, namely CaCl₂ and CaSO₄·2H₂O formed stoichiometrically from the HCl and SO₂ removed, plus unreacted excess reagent.
8. Spot analyses are normalized over all detected elements, including the mineral matrix. Spot 1 was located on a dark matrix particle and Spot 2 on a bright, heavy-metal-enriched grain (Figure S2a, S2b).
9. The area analysis was normalized over the eight heavy metals quantified and excludes light and matrix elements (O, Si, Ca, Al, Fe); n.q. denotes not quantified. The two normalisation bases are not directly comparable, and the higher heavy-metal percentages in the area column partly reflect the exclusion of matrix elements rather than genuine spatial enrichment.
10. Methane from IPCC first-order decay (Section S4.3); remaining gas constituents derived from the typical landfill gas composition of Tchobanoglous et al. (1993).[14]
11. Nielsen and Hauschild (1998), critical-condition assumption[15]
12. Jalili Ghazi Zadeh (2006)[16]
13. MA Abduli, E Safari (2003)[17]
14. allowance for local controls, instrumentation, valves, and compressed air, estimated in the absence of equipment-level data.
15. mechanised conveying is introduced in the alternative scenario only; waste is handled manually in the base scenario, where throughput is lower
16. boiler feedwater pump, condenser and cooling water pumps, cooling tower fans, induced draft fan and ash handling, estimated at 15 per cent of gross generation of 20,572.28 kilowatt-hours (Table S13). Not applicable to the base scenario, which has no energy recovery.
17. Emission Factors for Greenhouse Gas Inventories [33]
